# PathwayKO: An integrated platform for deciphering the systems-level signaling pathways

**DOI:** 10.3389/fimmu.2023.1103392

**Published:** 2023-03-23

**Authors:** Hannan Ai, Fanmei Meng, Yuncan Ai

**Affiliations:** ^1^ State Key Laboratory for Biocontrol, School of Life Sciences, Sun Yat-sen University, Guangzhou, China; ^2^ Department of Electrical and Computer Engineering, The Grainger College of Engineering, University of Illinois at Urbana-Champaign, Urbana, IL, United States; ^3^ National Center for Quality Supervision and Inspection of Automatic Equipment, National Center for Testing and Evaluation of Robots (Guangzhou), CRAT, SINOMACH-IT, Guangzhou, China; ^4^ The Second Affiliated Hospital, Guangdong Provincial Key Laboratory of Allergy & Clinical Immunology, Center for Inflammation, Immunity & Immune-mediated Disease, Sino-French Hoffmann Institute, Guangzhou Medical University, Guangzhou, Guangdong, China

**Keywords:** systems immunology, signaling pathways, network analysis, inflammation, innate immunity, microbial immunity, bioinformatics

## Abstract

Systems characterization of immune landscapes in health, disease and clinical intervention cases is a priority in modern medicine. High-throughput transcriptomes accumulated from gene-knockout (KO) experiments are crucial for deciphering target KO signaling pathways that are impaired by KO genes at the systems-level. There is a demand for integrative platforms. This article describes the PathwayKO platform, which has integrated state-of-the-art methods of pathway enrichment analysis, statistics analysis, and visualizing analysis to conduct cutting-edge integrative pathway analysis in a pipeline fashion and decipher target KO signaling pathways at the systems-level. We focus on describing the methodology, principles and application features of PathwayKO. First, we demonstrate that the PathwayKO platform can be utilized to comprehensively analyze real-world mouse KO transcriptomes (GSE22873 and GSE24327), which reveal systemic mechanisms underlying the innate immune responses triggered by non-infectious extensive hepatectomy (2 hours after 85% liver resection surgery) and infectious CASP-model sepsis (12 hours after CASP-model surgery). Strikingly, our results indicate that both cases hit the same core set of 21 KO MyD88-associated signaling pathways, including the Toll-like receptor signaling pathway, the NFκB signaling pathway, the MAPK signaling pathway, and the PD-L1 expression and PD-1 checkpoint pathway in cancer, alongside the pathways of bacterial, viral and parasitic infections. These findings suggest common fundamental mechanisms between these immune responses and offer informative cues that warrant future experimental validation. Such mechanisms in mice may serve as models for humans and ultimately guide formulating the research paradigms and composite strategies to reduce the high mortality rates of patients in intensive care units who have undergone successful traumatic surgical treatments. Second, we demonstrate that the PathwayKO platform model-based assessments can effectively evaluate the performance difference of pathway analysis methods when benchmarked with a collection of proper transcriptomes. Together, such advances in methods for deciphering biological insights at the systems-level may benefit the fields of bioinformatics, systems immunology and beyond.

## 1 Introduction

Systems characterization of immune landscapes in health, disease, and clinical intervention cases has become a priority in modern medicine. For instance, to reduce the high mortality rates of patients in the intensive care unit (ICU), insights into the complex mechanisms underlying systems immunology triggered by infectious or non-infectious traumatic surgical treatments must be further investigated through both bench-experimental and computational analyses (1–4). High-throughput transcriptomes accumulated in gene-knockout (KO) experiments are crucial for deciphering target KO signaling pathways that are impaired by KO genes at the systems-level (1, 5). KEGG pathways, as dominant examples, are curated manually with literature and experimental evidence, thus intuitively visualizing the signaling pathways that are supported by the literature and/or experimental evidence (6). However, existing methods of pathway enrichment analysis may not produce consistent results, or identify true target signaling pathways owing to intrinsic defects, as discussed in the literature (5, 7, 8).

Pathway enrichment analysis methods have evolved over decades from non-topology-based to topology-based approaches; the latter category generally performs better than the former category (5). The eminent non-topology-based methods include SAFE (9), GSEA (10), GSA (11) and PADOG (12). These methods employ entire gene sets. Unlike over-representation methods (e.g., the hypergeometric test) where a hypergeometric or binomial distribution is normally assumed (13, 14), advanced methods (e.g., SAFE and GSEA) are built on empirical distribution functions (9, 10). These statistical methods may not fit real data, thus preventing accurate predictions, as discussed in the literature (5, 14). The pioneering topology-based methods include ROntoTools_PE (15), SPIA (16) and ROntoTools_pDIS (5). These methods deploy differentially expressed genes (DEGs) with topology information on gene-gene interactions (5). Nonetheless, the criteria for selecting DEGs remain debatable (5). For instance, by a traditional approach, an arbitrary number of genes (e.g., top 5% or 10% of total genes present in the available KEGG pathways) are selected by using an arbitrary cutoff *p*-value (e.g., *p*<0.05) or fold-change (e.g., absolute log2FC>1.5) or their conjunction (5, 7, 8). These arbitrary cutoffs to select DEGs hinder a fair and reasonable comparisons under the same context, as discussed in the literature (5, 7). Meanwhile, approaches to evaluating performance differences among pathway analysis methods have recently evolved from traditional disease-target-pathways (7) toward known-KO pathways (5, 17). High-throughput transcriptomes accumulated in gene-KO experiments have offered unique opportunities to decipher target KO signaling pathways that are truly impaired by KO genes at the systems-level (1, 5). Thus, developing integrative platforms to conduct cutting-edge integrative pathway analysis and to evaluate the performance difference of methods has become an imminent frontier of research in the field.

We recently developed an in-house PathwayKO package and used it to analyze a transcriptome (GSE24327) that was recovered from mouse spleens 12 hours after a colon ascendens stent peritonitis (CASP)-model surgery coupled with *Myd88* gene-KO experiments (1, 2). The original bench-experiments suggested that the MyD88-deficient phenotype attenuated proinflammatory responses and thus reduced the mortality rate after CASP-model surgery (2). To elucidate the mechanisms underlying the observed phenomena, we designed the following three subtypes of GSE24327 data according to the original bench-experiments (2): GSE24327_A (septic KO MyD88 vs. septic WT) for comparing septic null (*Myd88* ^–/–^) with septic wild-type mice, GSE24327_B (septic KO MyD88 vs. untreated WT) for comparing septic null (*Myd88* ^–/–^) with untreated wild-type mice, and GSE24327_C (septic WT vs. untreated WT) for comparing septic wild-type with untreated wild-type mice (1). With the PathwayKO package, we successfully identified 21 KO MyD88-associated signaling pathways from each subtype and illustrated numerous key regulators (including ligands, receptors, adapters, transducers, transcription factors and cytokines) that were coordinately, significantly and differentially expressed at the systems-level, and were precisely marked on those target KO signaling pathways (1). Our results revealed the mechanisms underlying systems immunology triggered by the infectious CASP-model sepsis from the bioinformatics analysis perspective (1). We discussed the observed phenomena, including the “systemic syndrome”, “cytokine storm”, and “KO MyD88 attenuation”, as well as the proposed hypothesis of “spleen-mediated immune-cell infiltration” (1). We thereby anticipated that these mechanisms may serve as models for humans, and ultimately facilitate formulating research paradigms and composite strategies for the early diagnosis and prevention of sepsis (1). This case study appeals that the PathwayKO package has the potential to be constantly updated and widely used by the community.

The present article aims to describe the PathwayKO platform, focusing on its methodology, principles and application features. We applied the platform to analyze a real-world transcriptome (GSE22873) as a case study to elucidate the mechanisms underlying systems immunology triggered by non-infectious extensive hepatectomy in single- and double-null mice (3). We illustrate that the platform incorporates state-of-the-art methods of pathway enrichment analysis, statistics analysis, and visualizing analysis to conduct the cutting-edge integrative pathway enrichment analysis, which allows excavating target KO signaling pathways at the systems-level. We highlight that this integrated platform possesses but is not limited to the following advantageous features:

- The differentially expressed genes (DEGs) are statistically determined from data and are ready for pathway analysis under the same context;
- The ROC curves and key metrics are simultaneously calculated, both across methods and across data, in a pipeline fashion;
- The target KO signaling pathways are significantly identified and differentially marked by key regulators, which are coordinately, significantly and differentially expressed at the systems-level;
- The ROC curve-based statistics analysis is conducted under the same conditions to evaluate the performance difference of methods;
- Both interactive and pipeline modes can be chosen for desired computations.

Moreover, we demonstrate the application of the PathwayKO platform model-based assessments, and the results suggest that the PathwayKO platform can effectively evaluate the performance difference of pathway analysis methods when benchmarked on a collection of proper transcriptomes.

## 2 Framework overview of the PathwayKO platform

**Figure 1:**
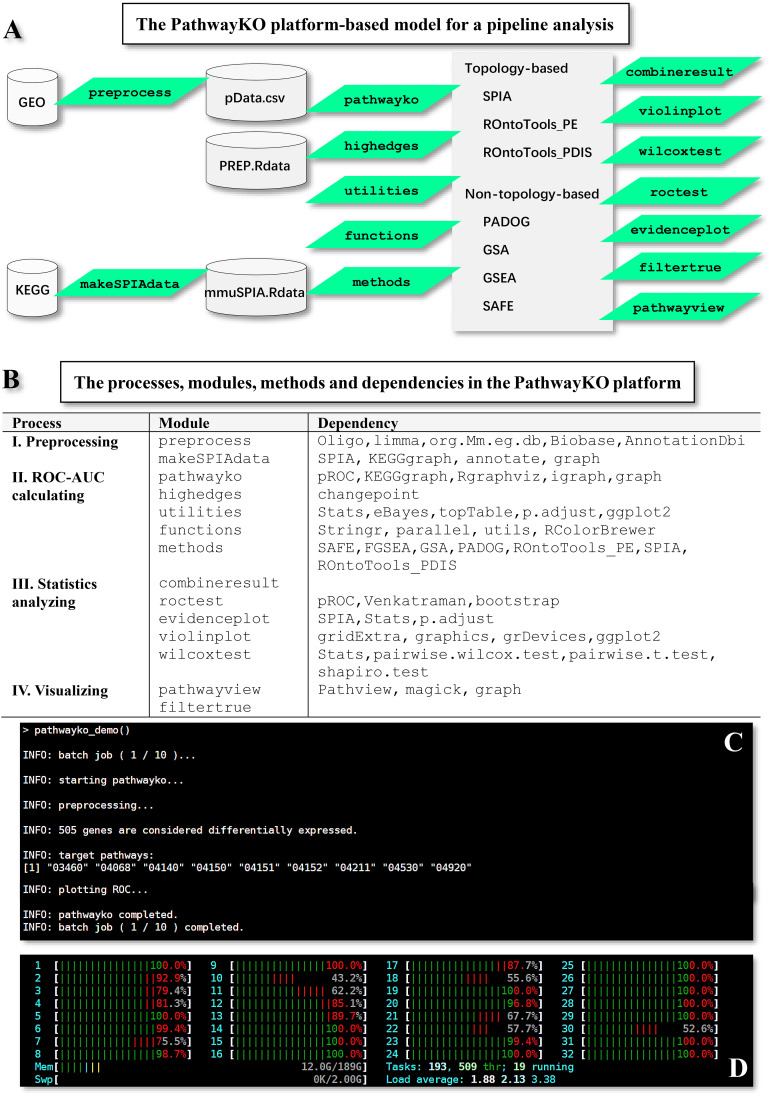
Framework overview of the PathwayKO platform. **(A)** Framework of the PathwayKO platform as systems software. **(B)** Processes, modules, methods and dependencies integrated in the PathwayKO platform. **(C)** Pipeline analysis with a collection of data, both across methods and across data. **(D)** Parallel computation.

The PathwayKO platform (**Figure 1**) currently incorporates and drives a set of internal and external packages (**Figure 1A**) coupled with diverse dependencies (**Figure 1B**) to pursue the integrative (I) preprocessing, (II) ROC-AUC calculating, (III) statistics analyzing, and (IV) visualizing processes. All external packages are adapted from the literature (9–20). The PathwayKO platform as systems software has integrated state-of-the-art methods of pathway enrichment analysis, statistics analysis, and visualizing analysis (**Figures 1A, B**), which work in a pipeline fashion (**Figures 1C, D**). The pathway enrichment analysis methods (**Figure 1A**) include the packages of non-topology- and topology-based methods, such as SAFE (9), GSEA (10), GSA (11), PADOG (12), ROntoTools_PE (15), ROntoTools_pDIS (5) and SPIA (16), which are widely used by the community. The statistics analysis methods (**Figure 1B**) include the packages of changepoint (18) and pROC (19). The visualizing analysis methods (**Figure 1B**) include the packages of pROC (19) and Pathview (20).

## 3 Methodology, principles and application features of the PathwayKO platform

This article aims to describe the PathwayKO platform from the perspectives of methodology, principles and application features with results from some of its modules (**Supplemental Figures S1**-**S4**), as exemplified by real-world case studies of GSE24327 (1, 2) and GSE22873 (3); these data were downloaded (as of March 19, 2021) from https://ncbi.nlm.nih.gov/geo/. The 333 mouse KEGG signaling pathways were downloaded (as of March 19, 2021) from https://www.kegg.jp/. Users should install the PathwayKO platform and utilize this platform to complete tasks following the tutorials step-by-step, provided as online supplemental materials (**Supplemental Figures S1**-**S4**). Users may also follow instructions in the user’s manual offered at https://github.com/allenaigit/pathwayko/tree/main/inst/docs/Users_manual.pdf.

### 3.1 Preprocessing process

#### 3.1.1 Feature 1: Automatic generation of intermediate data from one data

The preprocess module can preprocess the given GEO data (GSEXXX_RAW.tar and GSEXXX_series_matrix.txt.gz) stored in the assigned working directory (e.g., PathwayKO_platform) by utilizing the oligo (21) and limma (22, 23) packages for RMA normalization, which will generate intermediate data (pData.csv and PREP.RData). The preprocess module can handle most types of GEO data produced by the major types of machines thus far. Meanwhile, the makeSPIAdata module adapted from the SPIA package (16) can parse KEGG pathways to generate intermediate data (mmuSPIA.RData) ready for pathway enrichment analysis by topology-based methods (SPIA, ROntoTools_PE, and ROntoTools_pDIS). Only topology-based methods can utilize the topology information of gene-gene interactions (5, 16). All resulting output files are stored in the new directories automatically created and named after the given data (**Supplemental Figure S2**).

### 3.2 ROC-AUC calculating process

#### 3.2.1 Feature 2: Automatic selection of DEGs from one data

**Figure 2:**
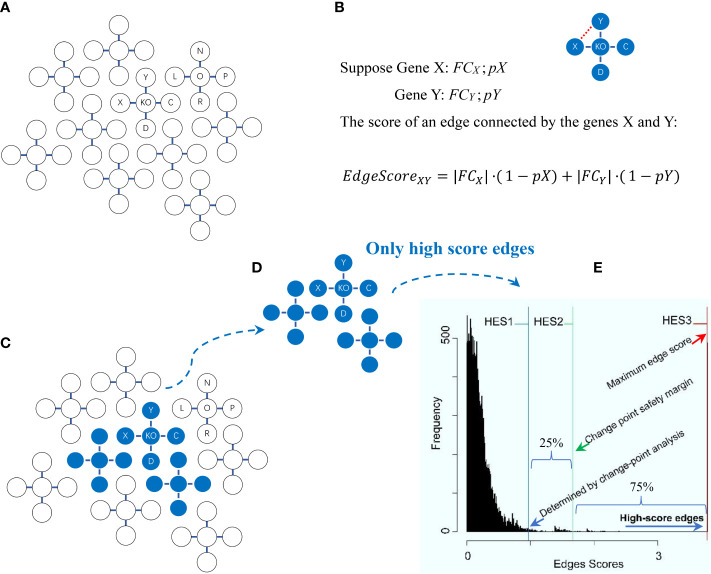
Schema of the high-edge-score (HES) approach with the change-point analysis method for statistically selecting differentially expressed genes. Explanations are presented in the main text. **(A)** A global graph is constructed. **(B)** An edge score for two genes (X, Y) in the global graph is calculated. **(C)** A change-point is statistically determined. **(D)** High score edges are statistically determined. **(E)** The distribution of edge scores is generated with each of three optional HES thresholds (HES1, HES2 and HES3).

The main module pathwayko can automatically select DEGs through enabling the HES (high-edge-score) approach (17), in addition to classical approaches (5, 7, 8). The main module pathwayko drives the external changepoint package (18), by which the change-point analysis method (see **Figure 1**) makes a statistical decision on choosing the differentially expressed genes (DEGs) based on the distribution of edge scores (**Figure 2**) that are constructed from data (17). The resulting output directory will be automatically created and named after the given data; the directory contains a set of output files including the list of DEGs, the list of knockout (KO) KEGG pathways, and the KO gene-associated subnetwork.

The principles behind the high-edge-score (HES) approach and the change-point analysis method for statistically selecting differentially expressed genes (DEGs) were modified from the literature (6, 17, 18, 23, 24), as briefly depicted below (**Figure 2**). First, a global graph is constructed (**Figure 2A**) by the external KEGGgraph package (24) based on available KEGG pathways (6); this graph comprises all known interactions among the entire gene sets present in the KEGG pathways. Second, the edge scores for all edges in the global graph are calculated (**Figure 2B**). Given two genes (X and Y) connected by an edge in the global graph, suppose *FC_X_* and *FC_Y_* are the values of the expression fold-change (FC) of X and Y, respectively, while *pX* and *pY* are the probability of observing *FC_X_* and *FC_Y_* just by chance, then the edge score between X and Y is calculated by the following formula (1):


(1) $$EdgeScore_{XY}=\left|FC_{X}\right|\cdot (1-pX)+\left|FC_{Y}\right|\cdot (1-pY)$$

where the FC value for gene X (or Y), *FC_X_* (or *FC_Y_*), is calculated by comparing the expression values of KO samples versus normal samples in each data. The *p*-value for gene X (or Y), *pX* (or *pY*), is calculated between the same groups (i.e., KO samples *vs*. normal samples) by using a moderated *t*-test (17). The FC values and *p*-values for all of these genes are calculated by using the functions eBayes and topTable from the external limma package (23). Third, a change-point is statistically determined (**Figure 2C**) by using the change-point analysis method from the external changepoint package (18), where a change-point is defined to be an inflection point after which the distribution curve becomes flat (18). Fourth, important edges are statistically determined (**Figure 2D**). They are connected by DEGs that are determined by a chosen HES threshold once initialized. Such DEGs connecting important edges are statistically selected, and ready for subsequent pathway enrichment analysis. Fifth, the distribution of edge scores is generated with three optional HES thresholds defined (**Figure 2E**). HES1 is the least HES (the lower boundary of the change point), the beginning of the flat area of a curve, recommended as default; HES2 is the extra 25% HES (25% safety margin of the change point), top 75% of the remaining scores to avoid selecting an overwhelming number of DEGs that may introduce false positives; and HES3 is the maximal HES (the upper boundary of the change point), the last edge connected by last two genes with the highest score. Finally, the differentially expressed genes (DEGs) are statistically selected by HES1, HES2 or HES3.

#### 3.2.2 Feature 3: Building ROC curves and calculating key metrics in a pipeline fashion from one data

The main module pathwayko can conduct the desired computations in a pipeline fashion, i.e., across methods over one data (see **Figure 1**). These computations include (i) building an ROC curve, (ii) computing the key metrics (**Table 1**), (iii) computing the AUC (with 95% CI, confidence interval) for the full area under the entire ROC curve, (iv) computing the partial AUCs (pAUC_SP and pAUC_SE) for the specific regions focusing on 90–100% specificity and sensitivity in both original and corrected formats (25), and (v) computing the 95% CIs for specificity and sensitivity. The entire set of key metrics (**Table 1**) for one data are then formatted as a summary output file (SUM.RData). The resulting output files are stored in a new directory automatically generated and named after the data. Such batch-computations intensively consume computing resources (CPUs, memory and storage). This is a time-limiting step.

**Table 1:** Key metrics used for the ROC curve-based statistics analysis.

Metrics	Definition	
False discovery rate (FDR)	$FDR=\frac{FPKO}{FPKO+TPKO}$	(2)
False positive rate (FPR)	$FPR=\frac{FPKO}{FPKO+TNKO}$	(3)
False negative rate (FNR)	$FNR=\frac{FNKO}{FNKO+TPKO}$	(4)
True positive rate (TPR)	$TPR\left(Sensitivity\right)=\frac{TPKO}{TPKO+FNKO}$	(5)
True negative rate (TNR)	$TNR\left(Specificity\right)=\frac{TNKO}{TNKO+FPKO}$	(6)
Accuracy	$Accuracy=\frac{TPKO+TNKO}{TPKO+FPKO+TNKO+FNKO}$	(7)
Precision	$Precision=\frac{TPKO}{TPKO+FPKO}$	(8)
Recall	$Recall=\frac{TPKO}{TPKO+FNKO}$	(9)
*p*-Threshold	*Youden’s_Threshold* = max(*Specificity*+*Sensitivity*)	(10)
AUC	Full area under the entire ROC curve	(11)
pAUC_SP	Partial area for a region of 90–100% specificity	(12)
pAUC_SE	Partial area for a region of 90–100% sensitivity	(13)

The principles behind the main module pathwayko (see **Figure 1**) are modified from the literature (5, 19, 26), as briefly depicted below. (i) By our definition, a true positive KO (TPKO) signaling pathway is a pathway that contains the knockout (KO) gene and was correctly identified to be a significantly impacted by that KO gene (e.g., at the pathway-level *p*-value < 0.001). (ii) A false positive KO (FPKO) signaling pathway is a pathway that does not contain the KO gene, and was not significantly impacted by that KO gene, but was still identified to be significantly impacted. A true negative KO (TNKO) signaling pathway is a pathway that does not contain the KO gene and was not significantly impacted by that KO gene; thus, it was not reported to be significantly impacted. A false negative KO (FNKO) signaling pathway is a pathway that contains the KO gene and was significantly impacted by that KO gene, but was not reported to be significantly impacted. (iii) For such a true-false case, the response versus prediction with a probability allows us to employ the external pROC package (19) to compute a set of key metrics defined by the above terms (**Table 1**). (iv) An ROC curve represents the tradeoff between specificity and sensitivity for every possible *p*-value (19). The Youden’s best *p*-value threshold (denoted as *p*-Threshold) is a *p*-value that defines an optimal point (specificity, sensitivity) on an ROC curve (26), where the sum of specificity and sensitivity is maximal (19, 25). Each point on an ROC curve represents a true KO (both true positive and false negative) signaling pathway in our cases. (v) Some key metrics with local properties (FDR, FPR, FNR, specificity, sensitivity, accuracy, precision and recall) collected at the *p*-threshold (**Table 1**) can be used to conduct a local comparison; others with global properties (AUC, pAUC_SP and pAUC_SE) can be applied to perform a global comparison. And these key metrics are appropriate for evaluating the performance difference of pathway analysis methods and for assessing the quality of data, both in terms of the ROC curve-based statistics analysis.

### 3.3 Statistics analyzing process

#### 3.3.1 Feature 4: The two-dimensional probability evidence plots

The internal evidenceplot module adapted from the SPIA package (16) can construct the two-dimensional probability evidence plots. The Adj.*p*-value is used to control the false discovery rate (FDR) for multiple testing (27, 28). Pathways above the oblique blue (or red) line are significant at 5% after BH-FDR (or Bonferroni) correction of the global *p*-values (*pG*), i.e., an adjusted Fisher’s product of *pPERT* and *pNDE* (16). Such plots can differentiate each data under comparison after having been analyzed by the SPIA method in view of the resolution along the X- and Y-axes (16). The highest resolution along the Y-axis, as indicated by the fine distribution, suggests that the most likely target pathways are identified, thus coinciding with its superiority among top-ranked TPKO signaling pathways (e.g., a list of top-30 ranked).

#### 3.3.2 Feature 5: The ROC curve-based statistical hypothesis testing

The internal roctest module adapted from the pROC package (19) can conduct the ROC curve-based statistical hypothesis testing both on the two ROC curves themselves (*via* the venkatraman method) and on the values of AUC, pAUC_SP and pAUC_SE (*via* the bootstrap method). The output files are stored in a new directory (roctest) automatically generated and named after the module.

#### 3.3.3 Feature 6: The ROC curve-based statistics analysis

The internal combineresult module can combine individual results of each data across methods, and format them to be a summary output file (STATS.RData). Thereby, the internal violinplot module and the internal wilcoxtest module can conduct the ROC curve-based statistics analysis, comparing the individual key metrics (**Table 1**) among the methods under comparison. All resulting output files are stored in the respective directories (combineresult, violinplot, and wilcoxtest), which are automatically created and named after the modules.

### 3.4 Visualizing process

#### 3.4.1 Feature 7: Highlighting target signaling pathways with key regulators

The internal filtertrue module can extract the signaling pathways, and the internal pathwayview module adapted from the Pathview package (20) can render them. The resulting output files are stored in the two directories automatically generated and named after the two modules (filtertrue and pathwayview). Key regulators, which are impacted by the same KO gene and are coordinately, significantly up- or down-regulated, can be differentially marked on the target signaling pathways. These rendered signaling pathways illuminate putative mechanisms underlying the KO phenotype at the systems-level.

## 4 Advanced batch-execution features of the PathwayKO platform

### 4.1 The work-flow logic of utilizing individual modules

#### 4.1.1 Feature 8: Optional modes for desired tasks

**Figure 3:**
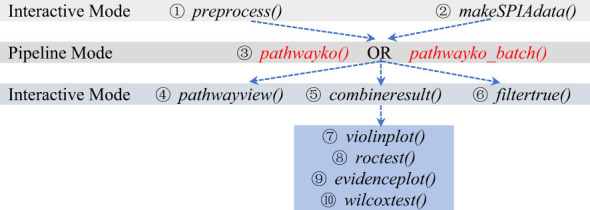
The modes of running individual modules of the PathwayKO platform.

The work-flow logic of utilizing individual modules of the PathwayKO platform is indicated below (**Figure 3**). The modules can be executed in interactive mode or in pipelined batch-execution mode.

### 4.2 Analyzing a collection of data in a pipeline fashion

#### 4.2.1 Feature 9: All-in-all computations in batch-execution mode

After a number of data having been preprocessed in a step-by-step manner and stored in the user’s working directory (**Supplemental Figure S2**), the user may conduct a batch-execution by typing the pathwayko_batch () command within the continued R session (**Supplemental Figure S3**). This command will start sequential operations: (i) to scan and identify data generated previously by the preprocess module; and (ii) to apply the main pathwayko module to conduct a batch-execution, performing the integrative KO pathway enrichment analysis in a pipeline fashion, both across methods and across data (**Supplemental Figure S3**).

### 4.3 Evaluating the performance of methods with a benchmark

#### 4.3.1 Feature 10: Evaluating the performance of methods under the same conditions

Once a collection of benchmark data have been previously preprocessed and stored in a user’s working directory (**Supplemental Figure S2**), the user should start a new R session to complete the desired batch-computations by following the offered tutorials, where some key parameters can be initialized in a step-by-step manner (**Supplemental Figure S3**). All resulting output files should be finally stored in the new directories that are automatically created and named after the modules (**Supplemental Figure S4**).

## 5 Results

### 5.1 Case study 1: Elucidating the mechanisms underlying systems immunology triggered by sterile extensive hepatectomy

#### 5.1.1 Assignment of transcriptomes reflecting the original bench-experiments

The original bench-experiments suggested that (i) wild-type (WT) mice had a high mortality rate after sterile 85% liver resection; (ii) single null *Myd88* (*Myd88* ^–/–^) mice had greatly reduced survival rates; (iii) double null *Myd88_ Ager* (*Myd88* ^–/–^ */Ager* ^–/–^) mice had even more greatly reduced survival rates; but (iv) single null *Ager* (*Ager* ^–/–)^ mice had drastically increased survival rates, which suggested opposing roles between MyD88 (encoded by *Myd88*) KO and RAGE (encoded by *Ager*) KO (3). The GSE22873 GEO data were created from tissues recovered from the remnants of mouse livers 2 hours after the sterile 85% liver resection in the indicated deficient and wild-type mice (3). These data were not subjected to bioinformatics analysis, similar to our perspectives, although some DEGs and pathways were suggested by primary analyses (3) with Pathway Express (16). To investigate the mechanisms underlying such phenomena raised by the original bench-experiments (3) from the perspective of systems immunology, we applied the PathwayKO platform to analyze the following six subtypes of data we assigned: GSE22873_M (KO *Myd88 vs*. WT), GSE22873_MA (KO *Myd88_Ager vs*. WT), GSE22873_A (KO *Ager vs*. WT), GSE22873_MAvM (KO *Myd88_Ager vs*. KO *Myd88*), GSE22873_MAvA (KO *Myd88_Ager vs*. KO *Ager*) and GSE22873_MvA (KO *Myd88 vs*. KO *Ager*). The results are presented as follows.

#### 5.1.2 The distribution of edge scores automatically estimated from a global graph

**Figure 4:**
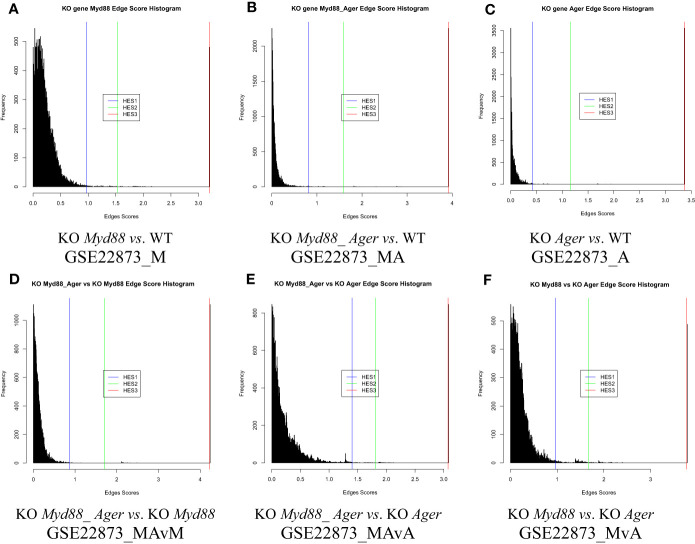
The distribution of edge scores with drastic variations among data. **(A)** GSE22873_M; **(B)** GSE22873_MA; **(C)** GSE22873_A; **(D)** GSE22873_MAvM; **(E)** GSE22873_MAvA; **(F)** GSE22873_MvA.

The distribution of edge scores varied drastically among data (**Figure 4**). Each showed a distinction, as marked by an HES threshold (HES1, HES2 and HES3, respectively), and reflected the different effects of experimental treatments.

#### 5.1.3 The differentially expressed genes statistically selected by an HES threshold

DEGs were statistically selected by using an HES threshold, as described in Methods (see **Figures 2**, **4**). By the HES1, HES2 and HES3 thresholds, respectively, GSE22873_M separately had 122 DEGs, 38 DEGs and 2 DEGs (**Supplemental Table S1**) with the top 10-ranked genes (*Myd88*, *Cxcl1*, *Fos*, *Nfatc3*, *Ccr1l1*, *Cxcr2*, *Ccr4*, *Rela*, *Jun* and *Xcr1*); GSE22873_MA had 130 DEGs, 57 DEGs and 2 DEGs (**Supplemental Table S2**) with the top 10-ranked genes (*Myd88*, *Cxcl1*, *H2-Bl*, *Ccr1l1*, *Ccr10*, *Cxcr2*, *Rela*, *Xcr1*, *Ccr1* and *Cxcr5*); and GSE22873_A had 135 DEGs, 27 DEGs and 2 DEGs (**Supplemental Table S3**) with the top 10-ranked genes (*H2-Bl*, *H2-T24*, *H2-M3*, *H2-Q6*, *Cdkn1a*, *Tapbp*, *AP1g1*, *Ap1b1*, *Ap1m1* and *H2-M10.1*). Similarly, by the HES1 threshold, there were 55 DEGs (with top-ranked *H2-Bl* and *H2-T24*) in GSE22873_MAvM (**Supplemental Table S4**); 67 DEGs (with top-ranked *Myd88* and *Cxcl1*) in GSE22873_MAvA (**Supplemental Table S5**); and 347 GEGs (with top-ranked *H2-Bl, H2-M10.1, Myd88* and *Cxcl1*) in GSE22873_MvA (**Supplemental Table S6**). Of note, rather than *Ager* itself, the MHC/antigen-related genes were ranked in the top 10 in GSE22873_A, which compared RAGE-null with wild-type mice. None of the above lists contained *Ager*, suggesting that (i) *Ager* itself was not yet significantly differentially expressed as early as 2 hours post-surgery when comparing RAGE-null against wild-type mice in GSE22873_A (KO *Ager vs*. WT), born by the transcriptome, in our current analysis; and (ii) RAGE (*Ager*)-mediated MHC/antigen-related genes play the most important roles, implying epigenetic effects.

#### 5.1.4 The KO gene-associated subnetwork automatically constructed from DEGs

**Figure 5:**
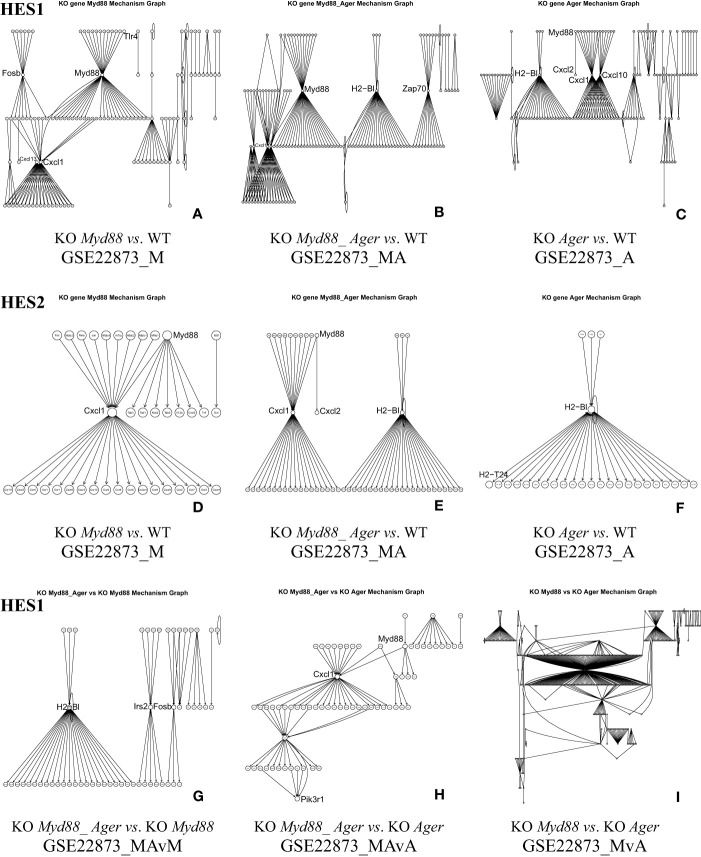
The KO gene-associated subnetworks comprised of DEGs statistically determined by HES1 and HES2. Landscapes are displayed since many node labels are unreadable by humans. Representative key nodes (e.g., Myd88, Cxcl1 and H2-Bl) are enlarged for clarity. **(A)** GSE22873_M, **(B)** GSE22873_MA, and **(C)** GSE22873_A at HES1; **(D)** GSE22873_M, **(E)** GSE22873_MA, and **(F)** GSE22873_A at HES2; **(G)** GSE22873_MAvM, **(H)** GSE22873_MAvA, and **(I)** GSE22873_MvA at HES1.

The KO gene-associated subnetwork was comprised of the statistically selected DEGs (**Figure 5**). The landscapes of these subnetworks explicitly revealed the global effects of experimental treatments at the systems-level. For instance, the top-ranked two genes (*Myd88* and *Cxcl1*) connected the last edge, as highlighted by the HES3 threshold (see **Figure 4**); this result suggests that the KO *Myd88*-signaling stimulated the highest responsive expression of *Cxcl1* in both GSE22873_M (**Supplemental Table S1**) and GSE22873_MA (**Supplemental Table S2**). Similarly, the top-ranked two genes (*H2-Bl* and *H2-T24*) connected the last edge (see **Figure 4**), suggesting that the KO *Ager*-signaling stimulated the highest responsive expression of *H2-Bl* and *H2-T24* in GSE22873_A (**Supplemental Table S3**). In addition, the remaining pairs displayed drastic variations, reflecting subtractive responses between the indicated pairs (**Figure 5**).

#### 5.1.5 The ROC curves with statistical features automatically built in a pipeline fashion

**Figure 6:**
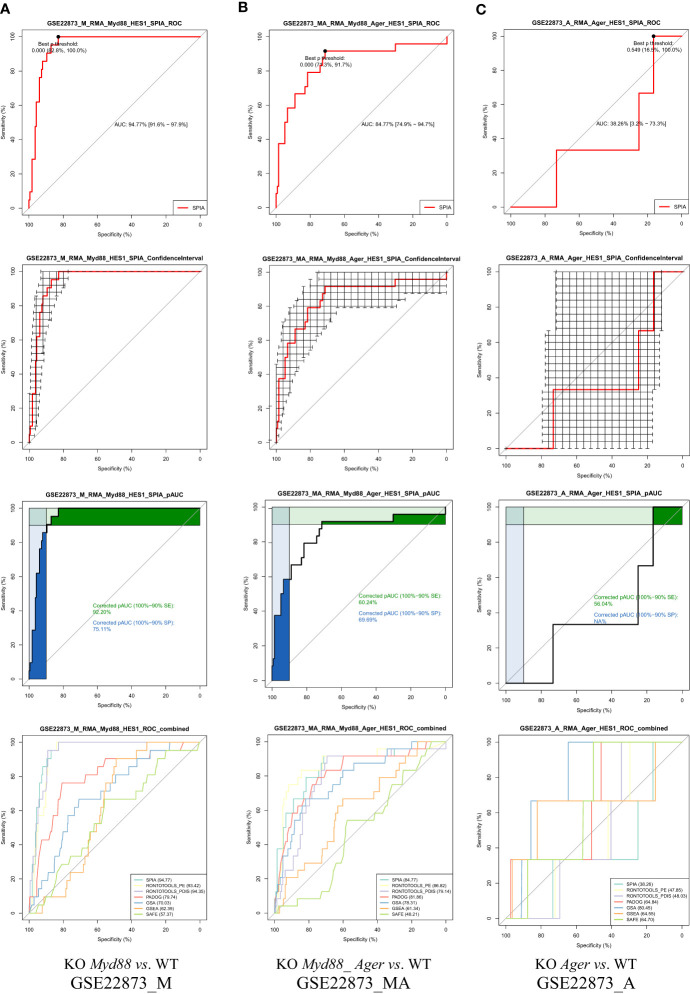
ROC curves with statistical features. GSE22873_A (KO *Ager* vs. WT) displays a weak signal to noise ratio, implying a lower degree of response to the stimulus. **(A)** GSE22873_M; **(B)** GSE22873_MA; **(C)** GSE22873_A.

The ROC curves with statistical features (**Figure 6**) were generated in a pipeline fashion, both across methods and across data. These results indicate the diverse landscapes of experimental treatments (data), which can discriminate the performance difference of pathway analysis methods under comparison. (i) An AUC with 95% CI and Youden’s best *p*-value threshold (noted shortly as *p*-Threshold) were marked on each ROC curve, indicating an optimal point (specificity, sensitivity) for a method. (ii) The 95% CIs of both specificity and sensitivity were marked on each ROC curve, indicating the deviation for a method on one data point. For instance, the drastic variation in 95% CIs shown in GSE22873_A (KO *Ager vs*. WT) data suggested weak signals over noise, which might impact the statistical hypothesis testing (19). (iii) The partial AUCs (pAUC_SP and pAUC_SE) of 90–100% specificity and sensitivity were marked on each ROC curve, implying a bias toward a higher specificity and sensitivity, respectively. (iv) The multiple ROC curves with AUCs intuitively displayed the superiority of methods when benchmarked on the same data (**Figure 6**).

#### 5.1.6 The ROC curve-based statistical hypothesis testing based on key metrics

**Figure 7:**
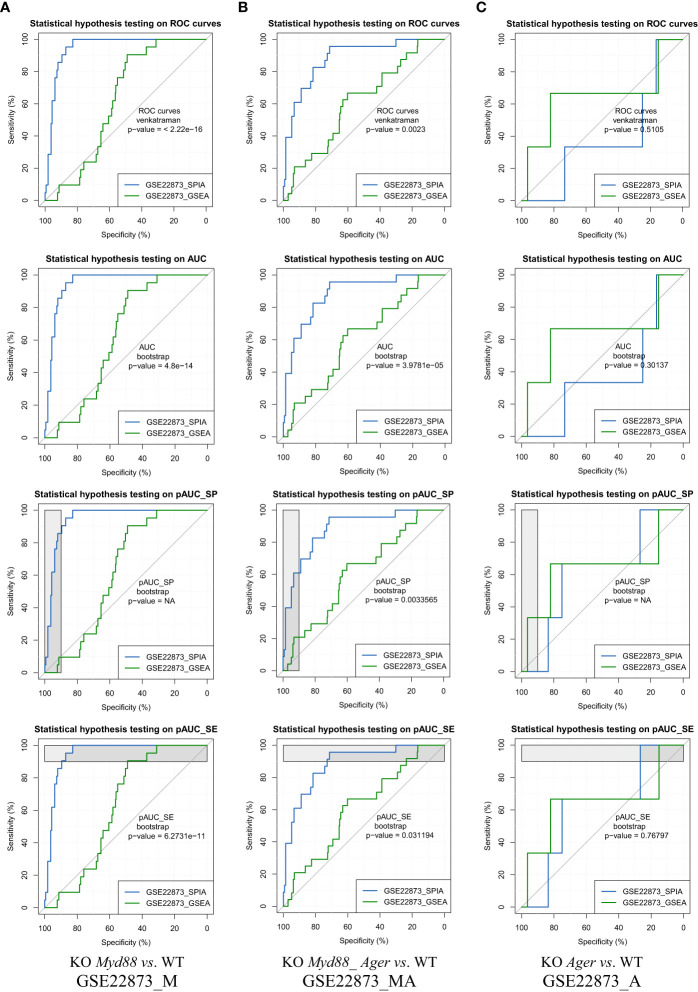
ROC curves-based statistical hypothesis testing of key metrics. The top-panel is testing the two ROC curves themselves (*via* the venkatraman method). The remaining panels are testing the AUC, pAUC_SP and pAUC_SE values (*via* the bootstrap method). **(A)** GSE22873_M; **(B)** GSE22873_MA; **(C)** GSE22873_A.

The ROC curve-based statistical hypothesis testing was conducted to indicate the significance level of the difference between the two methods (**Figure 7**). For instance, two methods (SPIA *vs*. GSEA) had a significant difference (e.g., *p*-value < 0.05) when benchmarked on GSE22873_M and GSE22873_MA, respectively, in view of both ROC curves themselves (*via* the venkatraman method) and the values of AUC, pAUC_SP and pAUC_SE (*via* the bootstrap method). SPIA was better than GSEA in these two cases (**Figures 7A, B**). However, the same two methods (SPIA *vs*. GSEA) showed no significant difference (e.g., *p*-value > 0.1) when benchmarked on GSE22873_A (**Figure 7C**). The reason is likely attributed to the drastic variations in the 95% CIs of specificity and sensitivity in GSE22873_A (see **Figure 6C**), which impact the resampling-based bootstrap assessments used for the ROC curves-based statistical hypothesis testing (19).

#### 5.1.7 The two-dimensional probability evidence plots

**Figure 8:**
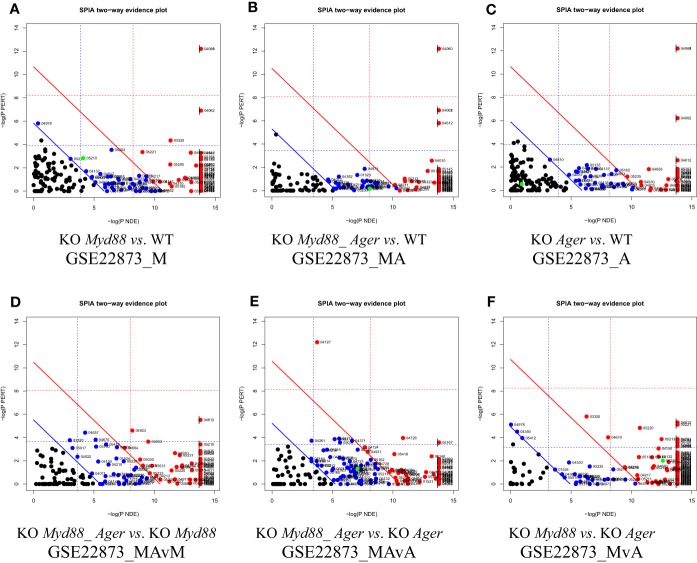
Two-dimensional probability evidence plots. A randomly assigned signaling pathway (green dot) located above or below the lines of significance level suggests the intuitive difference in fine distributions among data. **(A)** GSE22873_M; **(B)** GSE22873_MA; **(C)** GSE22873_A; **(D)** GSE22873_MAvM; **(E)** GSE22873_MAvA; **(F)** GSE22873_MvA.

The two-dimensional probability evidence plots were created for six data (**Figure 8**), which discriminate these data in view of the resolution along the X- and Y-axes. The highest resolution along the Y-axis suggests that the most likely target pathways were identified by SPIA, as indicated by the fine distribution.

#### 5.1.8 The true positive knockout signaling pathways statistically identified

We identified diverse true KO signaling pathways, which contained target KO gene(s), including 21 from GSE22873_M (KO *Myd88 vs*. WT), 23 from GSE22873_MA (KO *Myd88*_ *Ager vs*. WT), 3 from GSE22873_A (KO *Ager vs*. WT) and 24 from GSE22873_MvA (KO *Myd88 vs*. KO *Ager*) (**Table 2**; **Supplementary Table S7**). The majority of them were identified to be TPKO signaling pathways at a statistical significance level (e.g., pGFdr < 0.001 or 0.005) by our definitions described in Methods. (i) All 21 pathways from GSE22873_M were TPKO signaling pathways. (ii) Twenty-two out of 23 pathways were TPKO signaling pathways, whereas only 1 (mmu05010: Alzheimer disease) out of 23 pathways from GSE22873_MA was a false negative knockout (FNKO) signaling pathway; i.e., this pathway contained a target KO gene and should have been significantly impacted, but was not identified to be positive at a significance level (pGFdr > 0.005). (iii) Only 1 (mmu04933: AGE-RAGE signaling pathway in diabetic complications) out of 3 pathways from GSE22873_A was a TPKO signaling pathway, whereas the remaining two pathways were FNKO signaling pathways (pGFdr > 0.005). (iv) Twenty-three out of 24 pathways from GSE22873_MvA were TPKO signaling pathways, whereas only 1 (mmu05150: Staphylococcus aureus infection) out of 24 pathways was an FNKO signaling pathway (pGFdr > 0.005) (**Supplementary Table S7**).

**Table 2:** True KO signaling pathways identified by SPIA from GSE22873.

Order	Pathway ID	Pathway Name	Status	pSize	DEGs (%)	pGFdr	
Pathways identified from GSE22873_M (KO *Myd88 vs.* WT)							
**1**	**mmu04620**	**Toll-like receptor signaling pathway**	**Inhibited**	**87**	**36 (41.38)**	**1.03E-33**	
2	mmu05161	Hepatitis B	Inhibited	149	36 (24.16)	1.20E-24	
3	mmu05140	Leishmaniasis	Inhibited	66	23 (34.85)	9.51E-20	
4	mmu05152	Tuberculosis	Inhibited	158	32 (20.25)	2.20E-19	
5	mmu04621	NOD-like receptor signaling pathway	Inhibited	148	30 (20.27)	6.91E-18	
6	mmu05162	Measles	Inhibited	131	28 (21.37)	6.91E-18	
7	mmu05142	Chagas disease (American trypanosomiasis)	Inhibited	99	23 (23.23)	4.43E-15	
8	mmu05169	Epstein-Barr virus infection	Inhibited	185	29 (15.68)	1.16E-14	
9	mmu05164	Influenza A	Inhibited	143	26 (18.18)	1.40E-14	
10	mmu05145	Toxoplasmosis	Inhibited	105	22 (20.95)	2.62E-14	
11	mmu05170	Human immunodeficiency virus 1 infection	Inhibited	194	28 (14.43)	2.16E-13	
12	mmu05135	Yersinia infection	Inhibited	116	22 (18.97)	7.84E-13	
13	mmu05133	Pertussis	Inhibited	67	17 (25.37)	2.36E-12	
14	mmu04064	NF-kappa B signaling pathway	Inhibited	92	18 (19.57)	1.94E-10	
15	mmu05168	Herpes simplex virus 1 infection	Inhibited	340	31 (9.12)	3.64E-10	
16	mmu05132	Salmonella infection	Inhibited	193	24 (12.44)	5.73E-10	
17	mmu05134	Legionellosis	Inhibited	51	13 (25.49)	2.56E-09	
18	mmu05235	PD-L1/PD-1 checkpoint pathway in cancer	Inhibited	84	15 (17.86)	1.38E-08	
19	mmu04010	MAPK signaling pathway	Inhibited	281	25 (8.90)	3.65E-07	
20	mmu05144	Malaria	Inhibited	48	9 (18.75)	1.23E-05	
21	mmu05143	African trypanosomiasis	Inhibited	31	7 (22.58)	0.000126	
Pathways identified from GSE22873_MA (KO *Myd88*_*Ager vs.* WT)							
1	mmu05170	Human immunodeficiency virus 1 infection	Inhibited	194	61 (31.44)	2.18E-50	
**2**	**mmu04620**	**Toll-like receptor signaling pathway**	**Inhibited**	**87**	**44 (50.57)**	**1.24E-45**	
3	mmu05169	Epstein-Barr virus infection	Activated	185	53 (28.65)	8.65E-41	
4	mmu05142	Chagas disease (American trypanosomiasis)	Inhibited	99	35 (35.35)	2.30E-29	
5	mmu05162	Measles	Inhibited	131	38 (29.01)	1.42E-28	
6	mmu05161	Hepatitis B	Inhibited	149	40 (26.85)	1.63E-28	
7	mmu05135	Yersinia infection	Inhibited	116	35 (30.17)	1.61E-26	
8	mmu05235	PD-L1/PD-1 checkpoint pathway in cancer	Inhibited	84	31 (36.91)	1.94E-26	
9	mmu05168	Herpes simplex virus 1 infection	Inhibited	340	50 (14.71)	6.55E-24	
10	mmu04621	NOD-like receptor signaling pathway	Activated	148	33 (22.30)	1.24E-20	
11	mmu05140	Leishmaniasis	Inhibited	66	23 (34.85)	3.92E-19	
12	mmu05164	Influenza A	Inhibited	143	31 (21.68)	7.33E-19	
13	mmu05152	Tuberculosis	Inhibited	158	31 (19.62)	1.74E-17	
14	mmu05133	Pertussis	Inhibited	67	21 (31.34)	2.00E-16	
15	mmu05145	Toxoplasmosis	Inhibited	105	23 (21.91)	1.60E-14	
16	mmu04064	NF-kappa B signaling pathway	Inhibited	92	22 (23.91)	5.09E-14	
17	mmu05132	Salmonella infection	Inhibited	193	26 (13.47)	5.18E-11	
**18**	**mmu04933**	**AGE-RAGE signaling pathway in diabetics**	**Activated**	**98**	**18 (18.37)**	**1.58E-09**	
19	mmu05134	Legionellosis	Inhibited	51	13 (25.49)	7.03E-09	
20	mmu04010	MAPK signaling pathway	Inhibited	281	22 (7.83)	6.66E-06	
21	mmu05144	Malaria	Inhibited	48	9 (18.75)	2.00E-05	
22	mmu05143	African trypanosomiasis	Inhibited	31	7 (22.58)	0.000149	
23	mmu05010	Alzheimer disease	Activated	311	13 (4.18)	0.158326	
Pathways identified from GSE22873_A (KO *Ager vs.* WT)							
**1**	**mmu04933**	**AGE-RAGE signaling pathway in diabetics**	**Activated**	**98**	**10 (10.20)**	**0.002314**	
2	mmu05150	Staphylococcus aureus infection	Activated	76	3 (3.95)	0.476408	
3	mmu05010	Alzheimer disease	Activated	311	8 (2.57)	0.638874	

True positive knockout (TPKO) signaling pathway (pGFdr < 0.001 or 0.005) and false negative knockout (FNKO) signaling pathway (pGFdr > 0.005) are distinguishable. Bold pathways are discussed in the main text.

We categorized target TPKO signaling pathways into the following two groups (1): the basal signaling pathway that is solely responsible for the KO-gene phenotype; and the composite signaling pathway that embeds the named basal signaling pathway. Here, the basal signaling pathway for KO *Myd88* was the Toll-like receptor signaling pathway (mmu04620), while the composite signaling pathways were the remaining *Myd88*-containing signaling pathways (**Table 2**; **Supplementary Table S7**). Similarly, the basal signaling pathway for KO *Ager* was the AGE-RAGE signaling pathway in diabetic complications (mmu04933), while the composite signaling pathways were the remaining *Ager*-containing pathways (**Table 2**; **Supplementary Table S7**). Diverse target TPKO signaling pathways were mainly linked to innate inflammatory responses.

#### 5.1.9 The target TPKO signaling pathways highlighted at the systems-level

**Figure 9:**
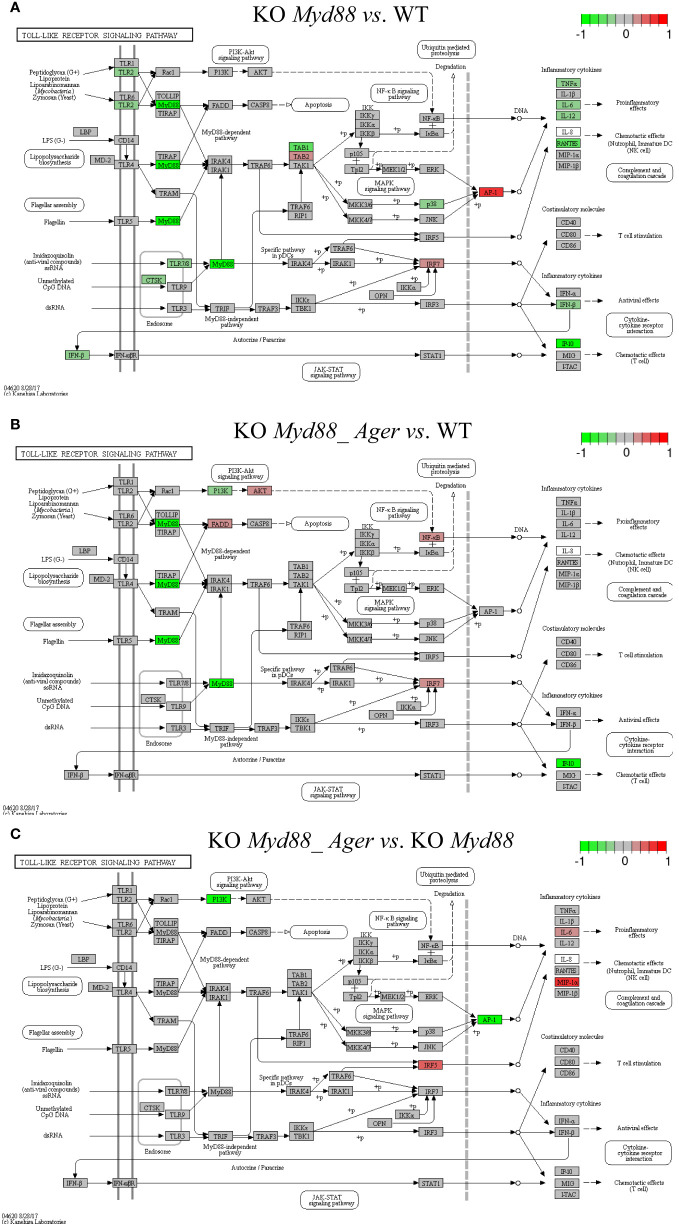
Comparison of the rendered Toll-like receptor signaling pathway (mmu04620) identified by the SPIA method. This target basal TPKO signaling pathway is solely responsible for the *Myd88*-null phenotype and is significantly inhibited in deficient mice compared to wild-type mice. The opposing roles of the **(A)** single *Myd88*-null and **(B)** double *Myd88_Ager*-null mutants highlight the global effects of key regulators (e.g., MyD88, NFκB, AP1 and cytokines) on orchestrating innate inflammatory responses at the systems-level. Treatment **(C)** reflects the subtractive difference between treatments **(A, B)**. **(A)** GSE22873_M; **(B)** GSE22873_MA; **(C)** GSE22873_MAvM.

**Figure 10:**
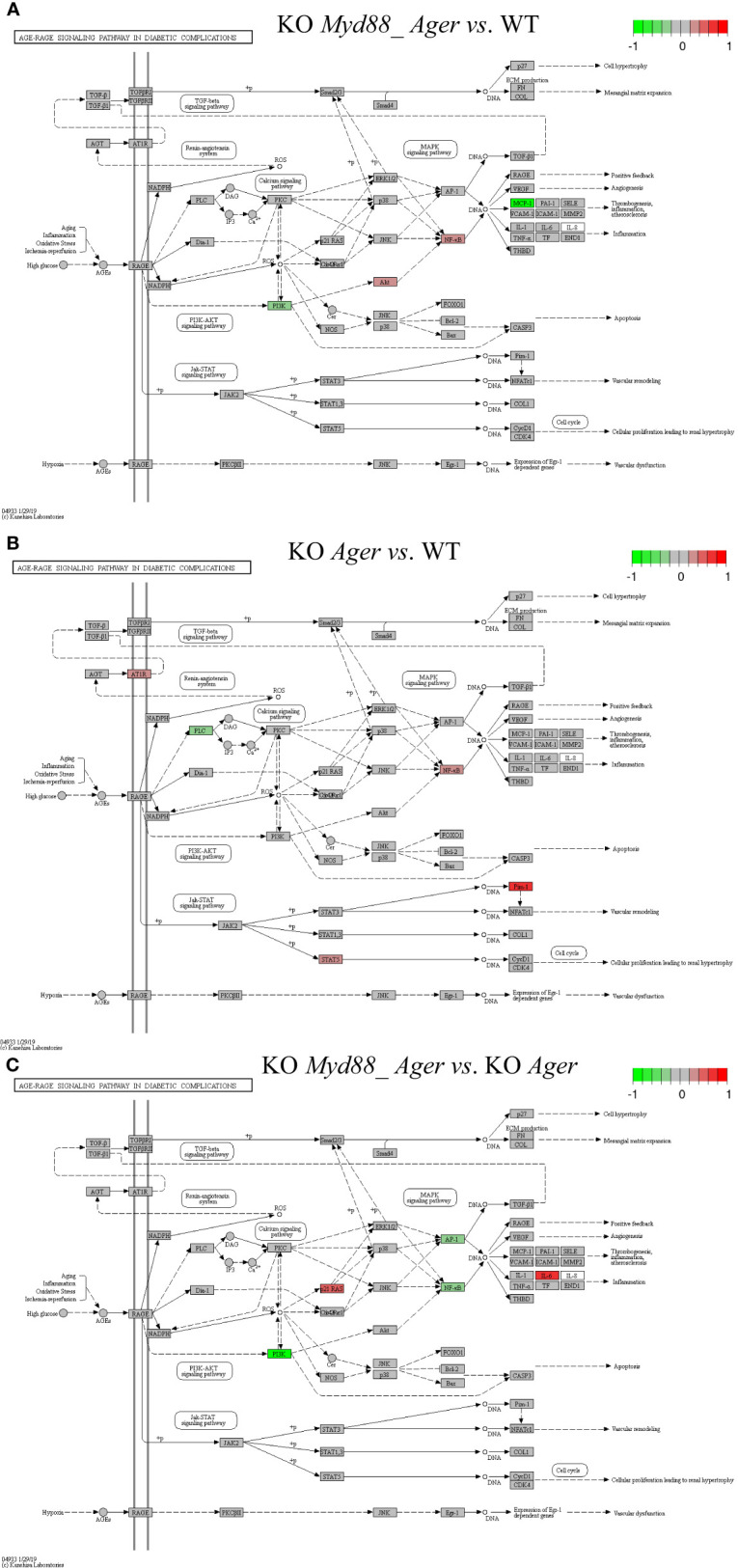
Comparison of the rendered AGE-RAGE signaling pathway in diabetic complications (mmu04933) identified by the SPIA method. This target basal TPKO signaling pathway is solely responsible for the Ager-null (KO RAGE) phenotype, and is significantly activated in deficient mice compared to wild-type mice as early as 2 hours post-surgery. The opposing roles of the **(A)** double Myd88_ Ager-null and **(B)** single Ager-null mutants highlight the global effects of key regulators (e.g., NFkB, MCP1 and PIM1) on orchestrating innate inflammatory responses at the systems-level. Treatment **(C)** reflects the subtractive difference between treatments **(A, B)**. **(A)** GSE22873_MA; **(B)** GSE22873_A; **(C)** GSE22873_MAvA.

**Figure 11:**
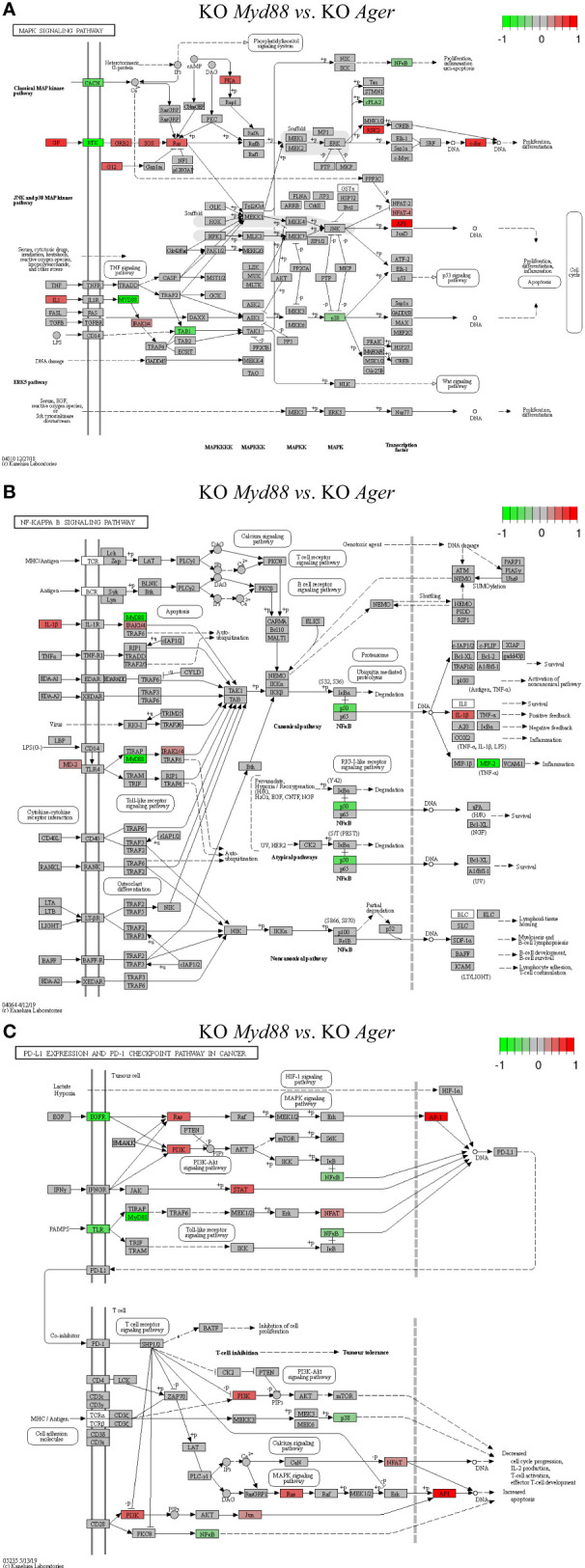
Comparison of the rendered target TPKO signaling pathways identified by the SPIA method from GSE22873_MvA (KO Myd88 vs. KO Ager). Explanations are presented in the main text. **(A)** The MAPK signaling pathway (mmu04010). **(B)** The NF-kappa B signaling pathway (mmu04064). **(C)** The PD-L1 expression and PD-1 checkpoint pathway in cancer (mmu05235).

The target TPKO signaling pathways identified by the SPIA method (see **Table 2**; **Supplementary Table S7**) were rendered with key regulatory proteins (encoded by *genes*). Key regulators included ligands, receptors, adapters, transducers, transcription factors and cytokines, which were coordinately, significantly and differentially expressed at the systems-level. Such key regulators may constitute a signaling-axis, as marked on a target TPKO signaling pathway. Some top-ranked target TPKO signaling pathways are highlighted below as examples (**Figures 9**–**11**).

##### 5.1.9.1 Scenario 1

The Toll-like receptor signaling pathway (mmu04620) was the target basal signaling pathway that was solely responsible for the *Myd88*-null phenotype in both single *Myd88*-null and double *Myd88_Ager*-null mutants (**Figure 9**). This pathway was significantly inhibited in deficient mice compared to wild-type mice (see **Table 2**; **Figure 9**). More than 40% of its 87 critical genes were DEGs that were coordinately, significantly and differentially up- or down-regulated as early as 2 hours post-surgery (see **Table 2**). The opposing roles of single *Myd88*-null and double *Myd88_Ager*-null mutants highlighted the global effects of key regulators (MyD88, NFκB, AP1 (FOS, JUN) and cytokines) on orchestrating innate inflammatory responses at the systems-level. (i) Key regulators in GSE22873_M (**Figure 9A**) included significantly down-regulated TLR2, MyD88 and p38 as well as significantly up-regulated TAB2, IRF7 and AP1 (FOS, JUN). These regulators were part of the TLR2-MyD88-TABs-AP1-ILs/CCL axis, which orchestrated the significant down-regulation of downstream inflammatory cytokines (TNFα, IL6, IL12, RANTES (CCL5), IFNβ and IP10 (CXCL10)), and reduced the proinflammatory, anti-viral and chemotactic effects at the systems-level. (ii) Key regulators in GSE22873_MA (**Figure 9B**) included significantly down-regulated MyD88, PI3K and IP10 (CXCL10) as well as significantly up-regulated FADD, AKT, NFκB and IRF7. Such regulators constituted the TLRs-MyD88-IRAKs-TABs-NFκB axis, the TLRs-PI3K-AKT-NFκB axis, the TLRs-MyD88-IRAKs-IRF7 axis, and the TLRs-MyD88-FADD-CASP8 axis. Most of these axes regulated the expression of inflammatory cytokines and induced proinflammatory and chemotactic effects. In addition, the TLRs-MyD88-FADD-CASP8 axis promoted apoptosis. (iii) The subtractive difference between the single *Myd88*-null and double *Myd88*_*Ager*-null mutants (**Figures 9A, B**) is displayed in **Figure 9C**. *Ager*-null mice, backgrounded in *Myd88*-null, induced down-regulation of PI3K and AP1 (FOS, JUN), but up-regulation of IRF5, IL6 and MIP-1α (CCL3); these factors further regulated the proinflammatory and chemotactic effects.

##### 5.1.9.2 Scenario 2

The AGE-RAGE signaling pathway in diabetic complications (mmu04933) was the target basal signaling pathway that was solely responsible for the *Ager*-null phenotype in both single *Ager*-null and double *Myd88*_*Ager*-null mutants (**Figure 10**). This pathway was significantly activated in the indicated deficient mice compared to wild-type mice (see **Table 2**; **Figure 10**). Less than 20% of its 98 critical genes were DEGs that were coordinately, significantly and differentially up- or down-regulated as early as 2 hours post-hepatectomy in the two treatments (see **Table 2**). The opposing roles of the single *Ager*-null and double *Myd88_ Ager*-null mutants highlighted the global effects of key regulators (NFκB, PIM1 and MCP1 (CCL2 and CCL12)) on orchestrating innate inflammatory responses at the systems-level. (i) Key regulators in GSE22873_MA (**Figure 10A**) included significantly down-regulated PI3K as well as significantly up-regulated AKT and NFκB, which were part of the AGEs-RAGE-PI3K-AKT-NFκB-MCP1 axis of the embedded PI3K-AKT signaling pathway (mmu04151). Activation of this axis determined the significant down-regulation of downstream inflammatory cytokine MCP1 (CCL2 and CCL12), thereby reduced the inflammation, atherosclerosis and thrombogenesis effects at the systems-level. (ii) Key regulators in GSE22873_A (**Figure 10B**) constituted multiple axes involved in regulating the single *Ager*-null phenotype. Firstly, key regulators included significantly down-regulated PLC and significantly up-regulated NFκB, and were part of the AGEs-RAGE-PLC-PKC-NFκB axis. Activation of this axis, coupled with the embedded MAPK signaling pathway (mmu04010) and the calcium signaling pathway (mmu04020), induced inflammation, atherosclerosis and thrombogenesis, in addition to RAGE-mediated positive feedback. Secondly, key regulators including significantly up-regulated STAT5 and PIM1 were part of the AGEs-RAGE-JAK2-STAT3-PIM1 axis. This axis, coupled with the embedded JAK-STAT signaling pathway (mmu04630), regulated vascular remodeling. Alternatively, the AGEs-RAGE-JAK2-STAT5-CycD1/CDK4 axis, coupled with the cell cycle signaling pathway, regulated cellular proliferation, leading to renal hypertrophy. Finally, the key regulator AT1R was significantly up-regulated and part of the AGT-AT1R-TGFβ-TGFβR-SMADs-p27/ECM axis. This axis, coupled with the embedded TGFβ signaling pathway (mmu04350), regulated cell hypertrophy and mesangial matrix expansion. Strikingly, RAGE (encoded by *Ager*) itself was not significantly up- or down-regulated in both cases when comparing the indicated null mutants against wild-type mice as early as 2 hours post-surgery in our current analysis, which implies that RAGE likely has an indirect effect of unclear epigenetics on regulating the single *Ager*-null (KO RAGE) phenotype at the systems-level. In addition, the subtractive difference between the two cases (**Figures 10A, B**) is displayed in **Figure 10C**. *Myd88*-null mice, backgrounded in *Ager*-null, induced down-regulation of PI3K, AP1 (FOS, JUN) and NFκB, but up-regulation of p21RAS, which further up-regulated the production of IL6, which is responsible for inflammation.

##### 5.1.9.3 Scenario 3

The treatment GSE22873_MvA (KO *Myd88 vs.* KO *Ager*) for a contrast between the *Myd88*-null and *Ager*-null mutants was interesting and plausible to elaborate the subtractive effects of KO *Myd88* against KO *Ager*. We identified 22 out of 23 significant TPKO signaling pathways (pGFdr < 0.001 or 0.005) from it (**Supplemental Table S7**). Firstly, two basal TPKO signaling pathways were significantly altered (**Supplemental Table S7**; **Figure S5**). (i) The Toll-like receptor signaling pathway (mmu04620) (**Supplemental Table S7**; **Figure S5A**) was ranked 2nd (with 55.17% DEGs out of the 87 critical genes) and was significantly inhibited. Key regulators (TLR2, TLR7/8, MyD88, CTSK, TAB1, p105, p38 and NFκB) were significantly down-regulated, whereas key regulators (MD2, PI3K, IRAK1, IRF7 and AP1 (FOS, JUN)) were significantly up-regulated. Such regulators constituted the TLR2-MyD88-IRAK1-TAB1-NFκB axis, the TLR2-PI3K-AKT-NFκB axis, the TLR7/8-MyD88-IRAK1-IRF7 axis, and the TLR2-MyD88-FADD-CASP8 axis. Activation of these axes resulted in up-regulation of inflammatory cytokines (IL1β and IL6) and down-regulation of inflammatory cytokines (IL12 and RANTES (CCL5)), which provoked proinflammatory effects and chemotactic (neutrophils and immature dendritic cells) effects at the systems-level. (ii) The AGE-RAGE signaling pathway in diabetic complications (mmu04933) (**Supplemental Table S7**; **Figure S5B**) was ranked 7th (with 36.73% DEGs out of the 98 critical genes) and was significantly activated. Key regulators (PLC, p38 and NFκB) were significantly down-regulated, whereas key regulators (PKC, PI3K, STAT3, STAT1/3, p21RAS, AP1 (FOS, JUN) and EGR1) were significantly up-regulated. Most importantly, activation of (i) the AGE-RAGE-PI3K-AKT-NFκB axis, coupled with the embedded PI3K-AKT signaling pathway, up-regulated IL1 and IL6, which are responsible for inflammation, as well as up-regulated VEGF, which is responsible for angiogenesis; (ii) alternatively, activation of the AGE-RAGE-PLC-PI3K-p38-AP1 axis or the AGE-RAGE-PI3K-ROS-p21RAS-p38-AP1 axis, coupled with the embedded MAPK signaling pathway and the calcium signaling pathway, up-regulated the expression of IL1, IL6 and VEGF; (iii) activation of the AGE-RAGE-JAK2-STAT3-axis, coupled with the embedded JAK-STAT signaling pathway, down-regulated the expression of PIM1, which is responsible for vascular remodeling; and (iv) activation of the AGE-RAGE-PKCβII-JNK-EGR1-axis increased the expression of EGR1-dependent genes leading to vascular dysfunction.

Secondly, multiple composite TPKO signaling pathways were also targeted, as described below (**Figure 11**; **Supplemental Table S7**). (i) The MAPK signaling pathway (mmu04010) (**Figure 11A**) was ranked 12th and was significantly activated (with 20.64% DEGs of the 281 critical genes) (**Supplemental Table S7**). On the classical MAPK pathway, several key regulators (CACN, RTK, cPLA2 and NFκB) were significantly down-regulated, whereas other key regulators (GF, G12, GRB2, SOS, Ras, PKA, RSK2 and cFOS) were significantly up-regulated, which induced proliferation and differentiation. On the JNK/p38 MAPK pathway, several key regulators (MyD88, TAB1 and p38) were significantly down-regulated, whereas other key regulators (IL1, IRAK1/4, NFAT4 and AP1 (FOS, JUN)) were significantly up-regulated, which stimulated proliferation, differentiation and inflammation. (ii) The NFκB signaling pathway (mmu04064) (**Figure 11B**) was ranked 21st and was significantly inhibited (with 21.74% DEGs out of the 92 critical genes) (**Supplemental Table S7**). Several key regulators (MyD88, NFκB (p50) and MIP2) were significantly down-regulated, whereas other key regulators (IL1β, MD2 and IRAK1/4) were significantly up-regulated, which resulted in increased IL1β leading to positive feedback and decreased MIP2 (CXCL1~CXCL3) leading to reduced inflammation. (iii) The PD-L1 expression and PD-1 checkpoint pathway in cancer (mmu05235) (**Figure 11C**) was ranked 15th and was significantly inhibited (with 33.33% DEGs of the 84 critical genes). Several key regulators (EGFR, TLR, MyD88, NFκB and p38) were significantly down-regulated, whereas other key regulators (PI3K, Ras, STAT, JUN, NFAT and AP1 (FOS, JUN)) were significantly up-regulated. Activation of the EGF-EGFR-Ras-Erk-AP1 axis coupled with the MAPK signaling pathway, the EGF-EGFR-PI3K-NFκB axis coupled with the PI3K-AKT signaling pathway, or the PAMPs-TLR-MyD88-NFAT/NFκB axis coupled with the Toll-like receptor signaling pathway stimulated the production of PD-L1 (CD274). The PD-L1/PD-1 complex led to further downstream effects, such as increased apoptosis as well as decreased IL-2 production, T-cell activation, effector T-cell development, and cell cycle progression. Auxiliary signaling pathways including the Toll-like receptor signaling pathway, the MAPK signaling pathway, the PI3K/AKT signaling pathway, the calcium signaling pathway, and the T-cell receptor signaling pathway were also embedded in the indicated target composite signaling pathways.

### 5.2 Case study 2: Evaluating the performance difference of pathway analysis methods

#### 5.2.1 Assignment of a benchmark

Based on the above comprehensive characterizations of individual data in this study and in our previous publication (1), we conclude that GSE22873_M, GSE22873_MA, GSE24327_A, GSE24327_B and GSE24327_C have strong signal to noise ratios when generated from the original bench-experiments (2, 3). These datasets are qualified to be included in a collection of qualified data that serves as a proper benchmark used to exemplify the different tasks in the present study (**Supplemental Figure S2**).

#### 5.2.2 All-in-all computations through batch-execution in a pipeline fashion

The operational scripts depict batch-executions, exemplifying how target KO signaling pathways are automatically deciphered at the systems-level in a pipeline fashion, both across methods and across data (**Supplemental Figure S3**). It takes approximately 25 minutes to complete the batch-computations across seven methods over one data on our high-performance workstation with the assigned configuration (see **Figure 1**; **Supplemental Figure S1**). This is a time-limiting step.

#### 5.2.3 Evaluating the performance difference of methods under the same conditions

**Figure 12:**
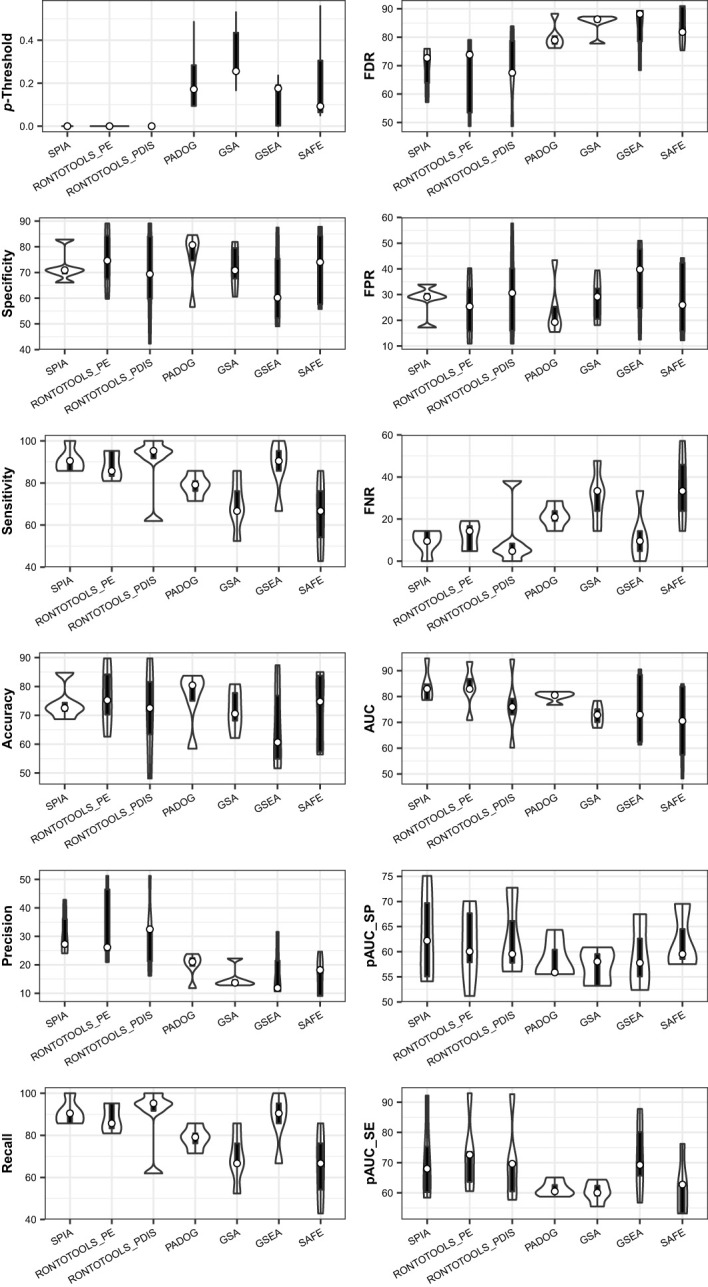
Violin plot of key metrics indicates the performance difference of seven methods when benchmarked on a collection of five data. The topology-based methods (SPIA, ROntoTools_PE and ROntoTools_pDIS) generally perform better than the non-topology-based methods (GSEA, GSA, SAFF and PADOG). SPIA is overall better than ROntoTools_PE and ROntoTools_pDIS.

The ROC curve-based statistical analyses (e.g., violin plot and Wilcox test) are suitable for assessing the performance difference of the pathway analysis methods under comparison (**Supplemental Figure S4**). The violin plot graph suggested that the topology-based methods (SPIA, ROntoTools_PE and ROntoTools_pDIS) generally performed better than the non-topology-based methods (GSEA, GSA, SAFF and PADOG); and that SPIA was overall better than ROntoTools_PE and ROntoTools_pDIS (**Figure 12**). These results coincide well with the findings of each individual data (see **Figures 6**, **7**, plus **Figure 2** therein Ref (1). However, the results of the Wilcox test (data not shown) on the global metrics (AUC, pAUC_SP and pAUC_SE) suggested that no significant difference was observed among methods when benchmarked on this collection of data.

#### 5.2.4 Key metrics suitable for a local comparison among methods

Collected at the optimal *p*-Threshold, the key metrics (FDR, FPR, FNR, specificity, sensitivity, accuracy, precision and recall) reflect the local properties of the ROC curves and are suitable for a local comparison among methods (see **Table 1**). With the chosen benchmark, the topology-based methods (SPIA, ROntoTools_PE and ROntoTools_pDIS) performed better than the non-topology-based methods (GSEA, GSA, SAFF and PADOG) in terms of *p*-Threshold and FDR; and GSEA displayed the poorest performance among the seven tested methods in terms of specificity, sensitivity, accuracy and precision (**Figure 12**).

#### 5.2.5 Key metrics suitable for a global comparison among methods

The key metrics including AUC, pAUC_SP and pAUC_SE reflect the overall properties of the ROC curves and are appropriate for a global comparison among methods (see **Table 1**). The partial AUCs (pAUC_SP and pAUC_SE) of the 90–100% specificity and sensitivity may display drastic variations on a case-by-case basis, thus measuring possible bias toward a higher specificity (true negative rate) or higher sensitivity (true positive rate). With the chosen benchmark, ROntoTools_PE performed better than ROntoTools_pDIS in terms of AUC, pAUC_SP and pAUC_SE; and SPIA had a higher specificity (pAUC_SP) but a lower sensitivity (pAUC_SE) than GSEA (**Figure 12**).

## 6 Discussion

### 6.1 Mechanisms underlying systems immunology triggered by sterile extensive hepatectomy

**Figure 13:**
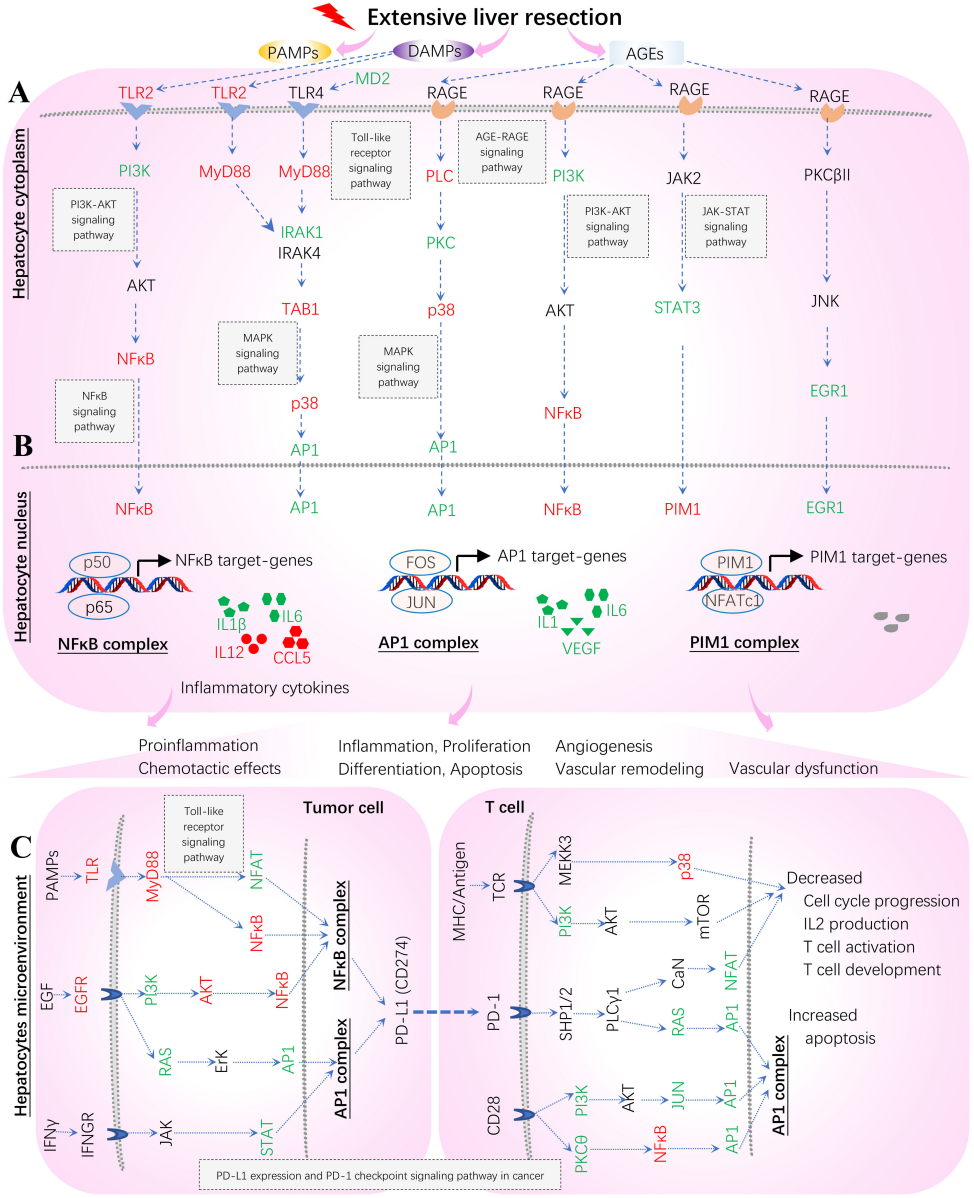
Hypothetical mechanisms underlying systems immunology triggered by extensive hepatectomy 2 hours post-resection in liver remnants. Key regulators are up (red)- and down (green)-regulated and marked on the indicated signaling axis coupled with target TPKO signaling pathways. These regulators coordinate MyD88 (*Myd88*) vs. RAGE (*Ager*) signaling transduction, thus orchestrating the regulation of innate inflammatory responses at the systems-level. The MyD88 signaling pathway responsible for proinflammatory responses is essential for survival consequent to 85% resection of the liver. MyD88 action stimulates the activation of NFκB and the subsequent up-regulation of key genes (including IL6) involved in liver regeneration responses. In contrast, RAGE opposes the actions of MyD88 signaling by suppressing NFκB, thereby reducing the activation of NFκB and the consequent production of cytokines (including IL1, IL1β, IL6, IL12 and CCL5), and by suppressing IL6-mediated phosphorylation of STAT3, which down-regulates PIM1 and reduces hyperplastic responses. The AP1 complex (FOS and JUN) acts as a hub in the present study. **(A)** Hepatocyte cytoplasm; **(B)** Hepatocyte nucleus; **(C)** Hepatocytes microenvironment (Tumor cells and T cells).

Our results endorse the main findings of the original bench-experiments in view of the signaling effects on innate immune responses after extensive hepatectomy (3), as summarized below. (i) MyD88 and RAGE distinctly modulated inflammation, proliferation and apoptosis. (ii) Deletion of MyD88 gene (*Myd88* null) significantly decreased the survival rate. (iii) Deletion of RAGE gene (*Ager* null) significantly increased the survival rate. (iv) Deletion of RAGE gene modulated NFκB activation, up-regulated PIM1 expression and increased phospho-STAT3. These results suggest that blockade of RAGE might rescue liver remnants from the multiple signals that preclude adaptive proliferation triggered primarily by MyD88 signaling pathways. We hypothesize mechanisms underlying systems immunology triggered by the sterile 85% liver resection as early as 2 hours post-hepatectomy in the liver remnants of wild-type mice (**Figure 13**). We exemplify five TPKO signaling pathways, including the Toll-like receptor signaling pathway, the AGE-RAGE signaling pathway in diabetic complications, the MAPK signaling pathway, the NFκB signaling pathway, and the PD-L1 expression and PD-1 checkpoint pathway in cancer (**Figure 13**). Nonetheless, it does not necessarily mean that other target TPKO signaling pathways (see **Table 2**; **Supplemental Table S7**) should be excluded, nor should these target pathways be altered at the same time or in a single event; rather, these pathways are targeted because of massive signaling-crosstalk at the systems-level (see **Figures 9**–**11**; **Supplemental Figure S5**), which are similar to those scenarios identified from the CASP-model sepsis previously discussed in detail (1). Hence, our results suggest diverse postinjury dysfunctions of the liver at the systems-level. This increases the difficulty of early diagnosis and prevention of liver failure after extensive hepatectomy because it is unlikely that systemic failures can be precisely attributed to a specific type of TPKO signaling pathway. Altogether, the hypothetical mechanisms we proposed from bioinformatics analysis and systems immunology provide novel informative cues that warrant bench-experimental validation at the systems-level in the future. We anticipate that such systems immunology in mice may serve as models for humans and ultimately guide formulating the research paradigms and composite strategies for the early diagnosis and prevention of diverse dysfunctions of livers post-hepatectomy in ICU patients who have undergone successful 85% hepatectomy for removing sick tissue and transplanting healthy tissue (3).

Our results offer a better understanding of the mechanisms underlying the innate inflammatory responses triggered by sterile 85% liver resection (3) from the perspectives of bioinformatics analysis and systems immunology (**Figure 13**). The *in vitro* original bench-experiments suggested that some key regulators (RAGE, TRL4, NFκB and PIM1) were significantly increased at 6 hours when comparing hepatectomy wild-type mice to sham-surgery wild-type mice (3). Unfortunately, the microarray profiling data from sham-surgery wild-type mice were absent from the transcriptome of GSE22873 (https://ncbi.nlm.nih.gov/geo/query/acc.cgi/GSE22873) produced from tissues sampled at 2 hours post-hepatectomy (3), which hindered us from comparing hepatectomy against sham surgery in wild-type mice. However, in the present study, we can infer a virtual treatment, GSE22873_MvA (KO *Myd88 vs.* KO *Ager*), that reflects a virtual comparison between resected wild-type mice and untreated wild-type mice (see **Figure 11**; **Supplementary Figure S5**). By which, we can elaborate the subtractive effects of KO *Myd88* over KO *Ager* by summarizing the up- and down-regulation of key regulators, as highlighted on the indicated signaling axis with target TPKO signaling pathways (see **Figure 11**; **Supplementary Figure S5**). Thereby, we can further depict innate inflammatory responses at the systems-level to stimulus from the sterile 85% liver resection in wild-type mice, where the influences of attenuation or enhancement by the deletion of MyD88 and RAGE are inversely adapted (**Figure 13**). The critical regulators are differentially marked on target TPKO signaling pathways, such as TLR2/4, MyD88, RAGE, NFκB, PI3K, STAT3, NFAT, PIM1, IL1, IL1β, IL6, IL12 and RANTES (CCL5) (see **Figure 11**; **Supplementary Figure S5**). These results are consistent with *in vitro* bench-experiment evidence (3, 29–34) and in line with recent reviews (4, 35–37). Intriguingly, the AP1 (FOS, JUN) complex emerges as a hub for the first time in this setting, and this complex plays a critical role in the signal transduction of DAMPs, PAMPs and AGEs that are triggered by extensive liver resection (**Figure 13**). DAMPs are released by damaged and dying cells (such as tumor cells) as endogenous host-derived molecules to promote sterile inflammation (4). DAMPs can also lead to the development of numerous inflammatory diseases, including autoimmune diseases, metabolic disorders, neurodegenerative diseases, and cancer (3, 4). The recognition of DAMPs is essential for tissue repair and regeneration (3, 4). The NFAT/AP1 transcriptional complex should be further explored, as a hub involved in regulating AP1-targeted genes at the systems-level (**Figure 13**). In addition to hepatocytes responding to stimulus from 85% hepatectomy (**Figures 13A, B**), the hepatocyte microenvironment is crucial to maintain proliferation, differentiation, apoptosis and inflammation (**Figures 13B, C**). Our findings are in line with recent advances in literature (38, 39). The NFAT/AP1 transcriptional complex was recently modulated by compounds that disrupt the NFAT : AP1:DNA complex, impairing transcription of cytokine genes (including IL2) important for the effector immune response (38). The transcription factor nuclear factor of activated T cells (NFAT) has a key role in both T-cell activation and tolerance and has emerged as an important target of immune modulation (38). NFAT directs the effector arm of the immune response in the presence of activator protein-1 (AP1), and T-cell anergy/exhaustion in the absence of AP1 (38). The PIM1/NFATc1 signaling pathway was recently suggested to be associated with impaired fibrosis resolution in aged mice after bleomycin injury (39).

Nevertheless, we did not recapture the original bench-experiment observation that the RAGE-dependent suppression of Glo1 (i.e., a detoxification pathway for pre-AGEs) enhanced the levels of AGEs and fueled a mechanism suppressing IL6 action (3). The RAGE-mediated down-regulation of Glo1 increased the production of AGEs (i.e., RAGE ligand) in the remnant, and AGEs *via* RAGE antagonized IL6, which in turn reduced the phosphorylation of STAT3 and thus blocked the up-regulation of PIM1 in wild-type mice compared to RAGE-null mice (3). In fact, *in vitro* bench-experiments (Affymetrix gene arrays, quantitative real-time PCR/ChIP assays, immunoblotting assays, etc.) assessing target chemicals (AGE, IL6, phospho-STAT3 and PIM1) in hepatocyte remnants 2 hours post-injury affirmed the observation that AGE-RAGE suppressed the IL6-induced phosphorylation of STAT3 and the up-regulation of PIM1 expression (3). These results were consistent with data from *ex vivo* bench-experiments in which hepatocytes isolated from both WT and RAGE-null mice 2 hours after 85% liver resection were incubated under the indicated conditions (3). Remarkably, AGE molecules can mediate extracellular signals (e.g., inflammation, aging, oxidative stress, ischemia-reperfusion and high glucose) to the RAGE receptor, but RAGE (encoded by *Ager*) itself was not significantly up- or down-regulated in our current analysis when comparing deficient mice to wild-type mice (KO *Ager vs*. WT) (see **Figure 10**; **Supplemental Figure S5).** Our findings tentatively suggest that RAGE may indirectly (*via* unclear epigenetics) regulate the KO RAGE (null *Ager*) phenotype and that more differentially expressed genes (DEGs) may be targeted and involved (see **Figures 9**–**11**; **Supplemental Figure S5**; **Tables S1**-**S6**); further study is advised. We speculate that the expression of RAGE itself and the RAGE-dependent suppression of Glo1 impairing IL-6 activity were not dominant enough to be significantly detected as early as 2 hours post-surgery, born by the transcriptome, when comparing deficient mice to wild-type mice (see **Figure 10**; **Supplemental Table S3**). The attractive role of RAGE-mediated epigenetics remains elusive.

### 6.2 Lessons from both non-infectious extensive hepatectomy and infectious CASP-model surgery

Strikingly, our data indicate that sterile extensive hepatectomy (3) and septic CASP-model surgery (1, 2) share 21 KO MyD88-associated target TPKO signaling pathways, including the Toll-like receptor signaling pathway, the NFκB signaling pathway, the MAPK signaling pathway, and the PD-L1 expression and PD-1 checkpoint pathway in cancer, alongside the pathways of bacterial, viral and parasitic infections (see **Table 2**; **Supplementary Table S7**). These findings suggest that the two cases share common fundamental mechanisms underlying the innate inflammatory responses as early as 2 hours and 12 hours post-surgery, respectively (2, 3). We infer that the down-regulation of MyD88-signaling, as marked on target TPKO signaling pathways (see **Figure 9**), should significantly diminish proinflammatory responses by eliminating the downstream “cytokine storm”, similar to the scenario we previously discussed in infectious CASP-model sepsis (1). We speculate that such target TPKO signaling pathways could orchestrate the innate immune responses at the early stage of post-surgery before diverse dysfunctions of post-injured organs occur (2, 3). Our results offer valuable informative cues of systems immunology that warrant bench-experiment validation at the systems-level in the future. We anticipate that common fundamental mechanisms for both surgeries in mice may serve as models for humans and ultimately guide formulating research paradigms and prevention strategies for the early diagnosis and prevention of dysfunctions of multiple organs at the early stage of post-surgery, which in turn should reduce the high mortality rates of ICU patients who have undergone successful traumatic surgical treatments.

Furthermore, our results favor recent proposals that TLRs, MyD88, RAGE and AGEs should have complex interactions to coordinate diverse mechanisms underlying innate immune responses at the systems-level (4, 40–42). Systems immunology triggered by MyD88- and TLRs-signaling in a wide range of immune-associated diseases, including mechanisms involved in diabetic complications (43), cardiovascular disease (44), metabolic disease (44) and other diseases associated with innate inflammatory signaling pathways (4, 45), deserves to be elusive in the context of gene-KO experiments through both experimental and computational analysis. Therefore, systems characterization of the immune landscapes of innate immune responses may dominate such research topics. We expect that the PathwayKO platform offers powerful tools for exploring such important topics and beyond.

### 6.3 The fundamental advantages of the PathwayKO platform

The PathwayKO platform has currently incorporated several eminent methods that have been widely used by the community (see **Figure 1**). It was not our intention to choose the most prestigious method(s) to integrate into the PathwayKO platform; rather, we intended to create an integrated platform, flexible enough to incorporate promising methods and to evaluate them under the same context. Multiple aspects were considered as follows. (i) The choice of method is data-dependent since each method may have individual bias toward specific data (5, 7). It is difficult to predict in advance which method should be chosen (5, 7, 8). (ii) Readers should be free to make their own judgments on proper method(s) toward data with complete resulting outputs in hand (see **Supplemental Figures S2**-**S4**); customers should also choose their favorite method(s) for routine analysis. (iii) It is possible for readers to remove methods that are performing consistently worse from the integrated PathwayKO platform by simply opting out of them when initializing parameters for a batch-execution (see **Supplemental Figures S1**, **S2**). (iv) The PathwayKO platform remains open to be constantly updated by incorporating new promising methods. Accordingly, we have established three fundamental advantages for the integrated PathwayKO platform, as highlighted below.

Firstly, a ground truth is chosen for the PathwayKO platform based on whether or not the knockout gene itself is included in the predicted pathway to evaluate method performance, modified from the literature (5). We defined the TPKO (true positive knockout) signaling pathway as the pathway that comprises of, and is significantly impacted by, the KO gene, and is correctly identified as a significantly positive pathway (e.g., at the pathway-level *p*-value < 0.0001). We further defined a set of terms modified from the literature (5), including FPKO, TNKO and FNKO signaling pathways, as described in the Methods section. Thereby, we defined a set of derivative metrics modified from the literature (19), including FDR, FPR, FNR, specificity, sensitivity, accuracy, precision, recall, AUC, pAUC_SP and pAUC_SE (see **Table 1**). Such that the universal rules in computer science for direct measurements on a prediction accuracy can be fitted to pathway enrichment analysis, which differs from indirect measurements used by the community (5, 7, 8, 46).

Secondly, for such a true-false case of prediction, the response versus prediction with a probability allows us employing the external pROC package (19) to build ROC curves and compute the set of key metrics (see **Table 1**). The performance difference of pathway enrichment analysis methods can be evaluated in terms of the ROC curve-based statistics analysis for the first time in this setting (see **Figures 6**–**8**, **12**). Moreover, each point on an ROC curve represents a true KO (both TPKO and FNKO) signaling pathway in our cases; and an ROC curve represents the tradeoff between specificity and sensitivity for every possible *p*-value (19). The Youden’s best *p*-value threshold (denoted as *p*-Threshold) is a *p*-value that defines an optimal point (specificity, sensitivity) on an ROC curve (19, 26), where the sum of specificity and sensitivity is maximal (19, 25). Hence, collected at the optimal *p*-Threshold, some key metrics (FDR, FPR, FNR, specificity, sensitivity, accuracy, precision and recall) with local properties can be used to conduct a local comparison, while others (AUC, pAUC_SP and pAUC_SE) with global properties can be employed to perform a global comparison (see **Table 1**). And such metrics are appropriate for evaluating the performance difference of methods (see **Figure 12**) and for assessing the quality of data (see **Figures 6**–**8**), both in terms of the ROC curve-based statistics analysis.

Finally, the HES (high-edge-scores) approach and the change-point analysis method (17, 26) are employed to statistically select DEGs (see **Figures 2**, **4**, **5**) in a pipeline fashion. This approach exerts two advantages: (i) a fair comparison can be made among the methods integrated in the PathwayKO platform in a pipeline fashion, rather than conducted one by one in a manually-interfering manner. This also saves hundreds of hours from non-stop computations under the same conditions when a large-scale collection of (benchmark) data should be analyzed through a large-scale batch-computation (see **Supplemental Figures S2**, **S3**); and (ii) the resulting output files are automatically created and named after each data (see **Supplemental Figures S3**, **S4**). Thereby, a fair comparison can be pursued under the same conditions, and thus a fair choice of method (or data) can be made in the same context. These features allow readers to obtain complete and broad insights into customer data in a timely manner at the systems-level (1).

### 6.4 The prospects of the PathwayKO platform

We note some limitations in the current status of the PathwayKO platform. Firstly, the complete resulting output files may be further integrated for more displaying ways, e.g., integrating the results into one output after applying a voting or merging mechanism (7, 8). Secondly, the PathwayKO platform deserves to explore other kinds of signaling pathways (47) (e.g., beyond the KO gene-associated signaling pathways defined by the choice of ground truth). For example, it might be possible that (i) a gene set containing a KO gene does not represent an actual regulated pathway (in the respective tissue and under respective condition, e.g., owing to epigenetic effects or missing co-factors); and (ii) the gene set of a truly affected pathway might not contain the KO gene. Thirdly, the PathwayKO platform does not fit to other kinds of data sources beyond GEO and KEGG pathways (47). Fourthly, a large-scale benchmark (constituted by KO transcriptomes) deserves to be created because a larger benchmark will yield a fairer comparison to evaluate the performance difference of methods under the same conditions (5). For which, qualified data must be selected, whose strong signals over noise must be validated through comprehensive characterizations, as revealed in the present study (see **Figure 6**). Finally, a large-scale benchmarking study toward gold-standard benchmarking remains to be explored, as another major challenge in the field (5, 7, 8, 46). All of these prospects deserve to be explored *via* updating the platform in the future.

## 7 Conclusion

This article exemplified case studies associated with systems immunology to elaborate the methodology, principle and application features of the PathwayKO platform. The PathwayKO platform can comprehensively analyze gene-knockout (KO) transcriptomes to uncover mechanisms underlying systems immunology triggered by non-infectious extensive hepatectomy born by GSE22873 in the present study and by infectious CASP-model surgery born by GSE24327 in our previous publication. The PathwayKO platform model-based assessments on the performance of pathway analysis methods can also effectively evaluate the performance difference of methods when benchmarked on a collection of KO transcriptomes. A proper method toward data can be inferred. Taken together, we recommend the PathwayKO platform be applicable to broad fields (e.g., immunology, microbiology, genetics, pharmacology, cancers biology, cell and developmental biology, etc.) as long as KO transcriptomes are available.

The PathwayKO platform is suitable for systems characterization of immune molecular landscapes of innate inflammatory responses in health, disease and clinical intervention cases through analyzing high-throughput transcriptomes from gene-knockout (KO) experiments. Real-world case studies suggest that both cases (GSE22873 and GSE24327) in the study share the same core set of 21 KO MyD88-associated target signaling pathways, including the Toll-like receptor signaling pathway, the NFκB signaling pathway, the MAPK signaling pathway, and the PD-L1 expression and PD-1 checkpoint pathway in cancer, alongside the pathways of bacterial, viral and parasitic infections. These findings suggest common fundamental mechanisms between the two cases and offer valuable insights into a better understanding of mechanisms underlying the innate inflammatory responses triggered by the non-infectious extensive hepatectomy (2 hours after 85% liver resection surgery in GSE22873) and the infectious CASP-model sepsis (12 hours after CASP-model surgery in GSE24327). Our results thus provide novel informative cues from the perspectives of bioinformatics analysis and systems immunology, which warrant further experimental validation in mice and may serve as models for humans.

## Data availability statement

The original contributions presented in the study are included in the article/**Supplementary Material**. Further inquiries can be directed to the corresponding authors.

## Author contributions

HA and YA designed the project HA designed and implemented the PathwayKO platform, conducted computations and analyzed data. HA, FM and YA interpreted results, wrote manuscript and approved the final manuscript. All authors contributed to the article and approved the submitted version.

## References

[1] Ai H Li B Meng F Ai Y . CASP-model sepsis triggers systemic innate immune responses revealed by the systems-level signaling pathways. Front Immunol (2022) 13:907646. doi: 10.3389/fimmu.2022.907646 35774781 PMC9238352

[2] Rime D Rossmann-Bloeck T Jusek G da Costa OP Holzmann B . Improved host defense against septic peritonitis in mice lacking MyD88 and TRIF is linked to a normal interferon response. J Leukoc Biol (2011) 90:613–20. doi: 10.1189/jib.1110602 21628330

[3] Zeng S Zhang QY Huang J Vedantham S Rosario R Ananthakrishnan R . Opposing roles of RAGE and MyD88 signaling in extensive liver resection. FASEB J (2012) 26:882–93. doi: 10.1096/fj.11-192997 PMC3365861 22075646

[4] Gong T Liu L Jiang W Zhou R . DAMP-sensing receptors in sterile inflammation and inflammatory diseases. Nat Rev Immunol (2020) 20:95–112. doi: 10.1038/s41577-019-0215-7 31558839

[5] Nguyen T Shafi A Nguyen T Draghici S . Identifying significantly impacted pathways: a comprehensive review and assessment. Genome Biol (2019) 20:203. doi: 10.1186/s13059-019-1790-4 31597578 PMC6784345

[6] Kanehisa M Sato Y Kawashima M Furumichi M Tanabe M . KEGG as a reference resource for gene and protein annotation. Nucleic Acids Res (2016) 44:D475–62. doi: 10.1093/nar/gkv1070 PMC4702792 26476454

[7] Nguyen T Mitrea C Draghici S . Network-based approaches for pathway level analysis. Curr Protoc Bioinf (2018) 1:28. doi: 10.1002/cpbi.42 30040185

[8] Nguyen T Mitrea C Tagett R Draghici S . DANUBE: Data-driven meta-analysis using unbiased empirical distributions - applied to biological pathway analysis. Proc IEEE (2017) 105:496–515. doi: 10.1109/JPROC.2015.2507119 PMC5919277 29706661

[9] Barry WT Nobel AB Wright FA . Significance analysis of functional categories in gene expression studies: a structured permutation approach. Bioinformatics (2005) 21:1943–9. doi: 10.1093/bioinformatics/bti260 15647293

[10] Subramanian A Tamayo P Mootha VK Mukherjee S Ebert BL Gillette MA . Gene set enrichment analysis: a knowledge-based approach for interpreting genome-wide expression profiles. Proc Natl Acad Sci USA (2005) 102:15545–50. doi: 10.1073/pnas.0506580102 PMC1239896 16199517

[11] Efron B Tibshirani R . On testing the significance of sets of genes. Ann Appl Stat (2007) 1:107–29. doi: 10.1214/07-AOAS101

[12] Tarca AL Draghici S Bhatti G Romero R . Down-weighting overlapping genes improves gene set analysis. BMC Bioinf (2012) 13:136. doi: 10.1186/1471-2105-13-136 PMC3443069 22713124

[13] Breitling R Amtmann A Herzyk P . Iterative group analysis (iGA): a simple method to enhance sensitivity and facilitate interpretation of microarray experiments. BMC Bioinf (2004) 5:34. doi: 10.1186/1471-2105-5-34 PMC403636 15050037

[14] Goeman JJ Buehlmann P . Analyzing gene expression data in terms of gene sets: methodological issues. Bioinformatics (2007) 23:980–7. doi: 10.1093/bioinformatics/btm051 17303618

[15] Tarca AL Draghici S Khatri P Hassan SS Mitta P Kim J . A novel signaling pathway impact analysis. Bioinformatics (2009) 25:75–82. doi: 10.1093/bioinformatics/btn577 18990722 PMC2732297

[16] Draghici S Khatri P Tarca AL Amin K Done A Voichita C . A systems biology approach for pathway level analysis. Genome Res (2007) 17:1537–45. doi: 10.1101/gr.6202607 PMC1987343 17785539

[17] Hanoudi S Donato M Draghici S . Identifying biologically relevant putative mechanisms in a given phenotype comparison. PloS One (2017) 12:e0176950. doi: 10.1371/journal.pone.0176950 28486531 PMC5423614

[18] Killick R Eckley IA . Changepoint: An r package for changepoint analysis. J Stat Soft (2014) 58:1–19. doi: 10.18637/jss.v058.i03

[19] Robin X Turck N Hainard A Tiberti N Lisacek F Sanchez J-C . pROC: an open-source package for r and s+ to analyze and compare ROC curves. BMC Bioinf (2011) 12:77. doi: 10.1186/1471-2105-12-77 PMC3068975 21414208

[20] Luo W Brouwer C . Pathview: an R/Bioconductor package for pathway-based data integration and visualization. Bioinformatics (2013) 29:1830–1. doi: 10.1093/bioinformatics/btt285 PMC3702256 23740750

[21] Carvalho BS Irizarry RA . A framework for oligonucleotide microarray preprocessing. Bioinformatics (2010) 16:2363–7. doi: 10.1093/bioinformatics/btq431 PMC2944196 20688976

[22] Smyth GK . Linear models and empirical bayes methods for assessing differential expression in microarray experiments. Stat Appl Genet Mol Biol (2004) 3:3. doi: 10.2202/1544-6115.1027 16646809

[23] Ritchie ME Phipson B Wu D Hu Y Law CW Shi W . *limma* powers differential expression analyses for RNA-sequencing and microarray studies. Nucleic Acids Res (2015) 43:e47. doi: 10.1093/nar/gkv007 25605792 PMC4402510

[24] Zhang JD Wiemann S . KEGGgraph: a graph approach to KEGG pathway in r and bioconductor. Bioinformatics (2009) 25:1470–1. doi: 10.1093/bioinformatics/btp167 PMC2682514 19307239

[25] McClish DK . Analyzing a portion of the ROC curve. Med Decis Mak (1989) 9:190–5. doi: 10.1177/0272989X8900900307 2668680

[26] Youden WJ . Index for rating diagnostic tests. Cancer (1950) 3:32–5. doi: 10.1002/1097-0142 15405679

[27] Benjamini Y Hochberg Y . Controlling the false discovery rate: A practical and powerful approach to multiple testing. J R Statist Soc B (1995) 57:289–300. doi: 10.1111/j.2517-6161.1995.tb02031.x12

[28] Benjamini Y Yekutieli D . The control of the false discovery rate in multiple testing under dependency. Ann Stat (2001) 29:1165–88. doi: 10.1214/aos/1013699998

[29] Cataldegirmen G Zeng S Ippagunta N Dun H Qu W Lu Y . RAGE limits regeneration after massive liver injury by coordinated suppression of TNF-alpha and NF-kappa b. J Exp Med (2005) 201:473–484. doi: 10.1084/jem.20040934 15699076 PMC2213026

[30] Nihira K Ando Y Yamaguchi T Kagami Y Miki Y Yoshida K . Pim-1 controls NF-kB signaling by stabilizing RelA/p65. Cell Death Differ (2010) 17:689–98. doi: 10.1038/cdd.2009.174 19911008

[31] Meloche J Paulin R Courboulin A Lambert C Barrier M Pierre Bonnet P . RAGE-dependent activation of the oncoprotein Pim1 plays a critical role in systemic vascular remodeling processes. Arterioscler Thromb Vasc Biol (2011) 31:2114–24. doi: 10.1161/ATVBAHA.111.230573 PMC3545710 21680901

[32] Chen X Xu C Zhang F Ma J . Microarray approach reveals the relevance of interferon signaling pathways with rat liver restoration post 2/3 hepatectomy at the cellular level. J Interferon Cytokine Res (2010) 30:525–39. doi: 10.1089/jir.2009.0111 20626293

[33] Ramasamy R Yan SF Schmidt AM . RAGE: therapeutic target and biomarker of the inflammatory response–the evidence mounts. J Leukoc Biol (2009) 86:505–12. doi: 10.1189/jlb.0409230 19477910

[34] Stepniak E Ricci R Eferl R Sumara G Sumara I Rath M . C-Jun/AP-1 controls liver regeneration by repressing p53/p21 and p38 MAPK activity. Genes Dev (2006) 20:2306–14. doi: 10.1101/gad.390506 PMC1553212 16912279

[35] Michalopoulos GK Bhushan B . Liver regeneration: biological and pathological mechanisms and implications. Nat Rev Gastroenterol Hepatol (2021) 8:40–55. doi: 10.1038/s41575-020-0342-4 32764740

[36] Kiseleva YV Antonyan SZ Zharikova TS Tupikin KA Kalinin DV Zharikov YO . Molecular pathways of liver regeneration: A comprehensive review. World J Hepatol (2021) 13:270–90. doi: 10.4254/wjh.v13.i3.270 PMC8006075 33815672

[37] Campana L Esser H Huch M Forbes S . Liver regeneration and inflammation: from fundamental science to clinical applications. Nat Rev Mol Cell Biol (2021) 22:608–24. doi: 10.1038/s41580-021-00373-7 34079104

[38] Mognol GP González-Avalos E Ghosh S Hogan PG . Targeting the NFAT:AP-1 transcriptional complex on DNA with a small-molecule inhibitor. PNAS (2019) 116:9959–68. doi: 10.1073/pnas.1820604116 PMC6525529 31019078

[39] Pham TX Lee J Guan J Caporarello N Meridew JA Jones DL . Transcriptional analysis of lung fibroblasts identifies PIM1 signaling as a driver of aging-associated persistent fibrosis. JCI Insight (2022) 7:e153672. doi: 10.1172/jci.insight.153672 35167499 PMC8986080

[40] Prantner D Nallar S Vogel SN . The role of RAGE in host pathology and crosstalk between RAGE and TLR4 in innate immune signal transduction pathways. FASEB J (2020) 34:15659–74. doi: 10.1096/fj.202002136R PMC8121140 33131091

[41] MacLean M Derk J Ruiz HH Juranek JK Ramasamy R Schmidt MM . The receptor for advanced glycation end products (RAGE) and DIAPH1: Implications for vascular and neuroinflammatory dysfunction in disorders of the central nervous system. Neurochem Int (2019) 126:154–64. doi: 10.1080/14789450.2018.1536551 PMC10976457 30902646

[42] Egaña-Gorroño L López-Díez R Yepuri G Ramirez LS Reverdatto S Gugger PF . Receptor for advanced glycation end products (RAGE) and mechanisms and therapeutic opportunities in diabetes and cardiovascular disease: Insights from human subjects and animal models. Front Cardiovasc Med (2020) 7:37. doi: 10.3389/fcvm.2020.00037 32211423 PMC7076074

[43] Senatus L López-Díez R Egaña-Gorroño L Liu J Hu J Daffu G . RAGE impairs murine diabetic atherosclerosis regression and implicates IRF7 in macrophage inflammation and cholesterol metabolism. JCI Insight (2020) 5:e137289. doi: 10.1172/jci.insight.137289 32641587 PMC7406264

[44] Senatus L MacLean M Arivazhagan L Egaña-Gorroño L López-Díez R Manigrasso MB . Inflammation meets metabolism: Roles for the receptor for advanced glycation end products axis in cardiovascular disease. Immunometabolism (2021) 3:e210024. doi: 10.20900/immunometab20210024 34178389 PMC8232874

[45] Li B Zou Z Meng F Raz E Huang Y Tao A . Dust mite-derived der f 3 activates a pro-inflammatory program in airway epithelial cells *via* PAR-1 and PAR-2. Mol Immunol (2019) 109:1–11. doi: 10.1016/j.molimm.2019.02.018 30836204

[46] Maleki F Ovens K Hogan DJ Kusalik AJ . Gene set analysis: Challenges, opportunities, and future research. Front Genet (2020) 11:654. doi: 10.3389/fgene.2020.00654 32695141 PMC7339292

[47] Rhee YS Wood V Dolinski K Draghici S . Use and misuse of the gene ontology annotations. Nat Rev Genet (2008) 9:509–15. doi: 10.1038/nrg2363 18475267

