# Supplementary material for: PathwayKO: An integrated platform for deciphering the systems-level signaling pathways
Source: Front Immunol. 2023 Mar 23;14:1103392. doi: 10.3389/fimmu.2023.1103392 (PMC10080220; doi:10.3389/fimmu.2023.1103392)
Supplement: Supplementary file 1 [file DataSheet_1.pdf]

Supplemental Materials

I. Tutorial for installation of the PathwayKO platform

To install the PathwayKO platform in a Linux session, users should create and enter into a working directory (e.g., PathwayKO_platform) and type command lines:

```
$ cd /home/PathwayKO_platform
PathwayKO_platform$ R
> library(devtools)
> devtools::install_github("allenaigit/pathwayko")
> sessionInfo()
```

The prerequisite packages including R (R 4.0.2+), Bioconductor (3.11+), external methods and diverse dependencies must be correctly installed before installing the PathwayKO platform (as an R package itself). The user may need to install and update the prerequisite packages for multiple rounds and should consult R's help page or IT support staff with package-installation questions. After all required packages have been correctly installed, the environment configuration should look like the example session information (Supplemental Figure S1), exemplifying the main items with correct R-versions that were used to analyze example data in this study.

II. Tutorial for step-by-step applications of the PathwayKO platform

Suppose the user defined a working directory (e.g., PathwayKO_platform), the user needs to start an R session and type the following command lines to preprocess one data (Supplemental Figure S2):

```
$ cd /home/PathwayKO_platform
PathwayKO_platform$ R
> library(oligo)
> library(limma)
> library(pathwayko)
> preprocess()
> makeSPIAdata()
```

To automatically select DEGs, the user should type the following command into the continued R session (Supplemental Figure S2):

```
> pathwayko()
```

To build ROC curves and calculate key metrics, the user should type the following command into the continued R session (Supplemental Figure S2):

```
> pathwayko()
```

To highlight the true positive knockout (TPKO) signaling pathways that are impacted by the same KO gene, the user should type the following command into the continued R session (Supplemental Figure S3):

```
> pathwayview()
```

To illustrate the substantial changes of probability evidence, the user should type the following command into the continued R session (Supplemental Figure S4):

```
> evidenceplot()
```

To compare the difference between two methods based on one data, the user should type the following command into the continued R session (Supplemental Figure S4):

```
> roctest()
```

To conduct the ROC curve-based statistics analysis based on a collection of data, the user should type the following commands into the continued R session (Supplemental Figure S4):

```
> combineresult()
> violinplot()
> wilcoxtest()
```

III. Tutorial for batch-computation applications of the PathwayKO platform

Once a collection of benchmark data have been previously preprocessed and stored in a user's working directory (Supplemental Figure S2), the user should start a new R session and type the following commands to conduct the desired batch-computations, where some key parameters can be initialized in a step-by-step manner (Supplemental Figure S3):

```
$ cd /home/PathwayKO_platform
PathwayKO_platform$ R
> library(oligo)
> library(limma)
> library(pathwayko)
> pathwayko_batch()
> combineresult()
> violinplot()
> wilcoxtest()
> filtertrue()
```

All resulting output files should be finally stored in the new directories that are automatically created and named after the modules (Supplemental Figure S4).

IV. Supplemental Data

A

```
ayc@MMBL-WS:~$ cd PathwayKO_platform/
ayc@MMBL-WS:~/PathwayKO_platform$ R

> sessionInfo()
R version 4.0.3 (2020-10-10)
Platform: x86_64-pc-linux-gnu (64-bit)
Running under: Ubuntu 20.04.3 LTS
Matrix products: default
BLAS:   /usr/lib/x86_64-linux-gnu/blas/libblas.so.3.9.0
LAPACK: /usr/lib/x86_64-linux-gnu/lapack/liblapack.so.3.9.0
locale:
 [1] LC_CTYPE=en_US.UTF-8          LC_NUMERIC=C
 [3] LC_TIME=zh_CN.UTF-8           LC_COLLATE=en_US.UTF-8
 [5] LC_MONETARY=zh_CN.UTF-8       LC_MESSAGES=en_US.UTF-8
 [7] LC_PAPER=zh_CN.UTF-8          LC_NAME=C
 [9] LC_ADDRESS=C                  LC_TELEPHONE=C
[11] LC_MEASUREMENT=zh_CN.UTF-8    LC_IDENTIFICATION=C
attached base packages:
[1] stats     graphics  grDevices utils     datasets  methods   base
loaded via a namespace (and not attached):
[1] compiler_4.0.3
```

B

```
> library(oligo)
Loading required package: BiocGenerics
Loading required package: parallel
Attaching package: 'BiocGenerics'
The following objects are masked from 'package:parallel':
    clusterApply, clusterApplyLB, clusterCall, clusterEvalQ,
    clusterExport, clusterMap, parApply, parCapply, parLapply,
    parLapplyLB, parRapply, parSapply, parSapplyLB
The following objects are masked from 'package:stats':
    IQR, mad, sd, var, xtabs
The following objects are masked from 'package:base':
    anyDuplicated, append, as.data.frame, basename, cbind, colnames,
    dirname, do.call, duplicated, eval, evalq, Filter, Find, get, grep,
    grepl, intersect, is.unsorted, lapply, Map, mapply, match, mget,
    order, paste, pmax, pmax.int, pmin, pmin.int, Position, rank,
    rbind, Reduce, rownames, sapply, setdiff, sort, table, tapply,
    union, unique, unsplit, which, which.max, which.min
Loading required package: oligoClasses
Welcome to oligoClasses version 1.50.4
Loading required package: Biobase
Welcome to Bioconductor
    Vignettes contain introductory material; view with
    'browseVignettes()'. To cite Bioconductor, see
    'citation("Biobase")', and for packages 'citation("pkgname")'.
Loading required package: Biostrings
Loading required package: S4Vectors
Loading required package: stats4
Attaching package: 'S4Vectors'
The following object is masked from 'package:base':
    expand.grid
Loading required package: IRanges
Loading required package: XVector
Attaching package: 'Biostrings'
The following object is masked from 'package:base':
    strsplit
No methods found in package 'RSQLite' for request: 'dbListFields' when loading 'oligo'
================================================================================
Welcome to oligo version 1.52.1
================================================================================

> library(limma)
Attaching package: 'limma'
The following object is masked from 'package:oligo':
    backgroundCorrect
The following object is masked from 'package:BiocGenerics':
    plotMA

> library(pathwayko)
```

C

```
> sessionInfo()
R version 4.0.3 (2020-10-10)
Platform: x86_64-pc-linux-gnu (64-bit)
Running under: Ubuntu 20.04.3 LTS
Matrix products: default
BLAS:   /usr/lib/x86_64-linux-gnu/blas/libblas.so.3.9.0
LAPACK: /usr/lib/x86_64-linux-gnu/lapack/liblapack.so.3.9.0
locale:
 [1] LC_CTYPE=en_US.UTF-8       LC_NUMERIC=C
 [3] LC_TIME=zh_CN.UTF-8        LC_COLLATE=en_US.UTF-8
 [5] LC_MONETARY=zh_CN.UTF-8    LC_MESSAGES=en_US.UTF-8
 [7] LC_PAPER=zh_CN.UTF-8       LC_NAME=C
 [9] LC_ADDRESS=C               LC_TELEPHONE=C
[11] LC_MEASUREMENT=zh_CN.UTF-8 LC_IDENTIFICATION=C
attached base packages:
[1] stats4    parallel  stats     graphics  grDevices utils     datasets
[8] methods   base
other attached packages:
 [1] pathwayko_0.1.1      limma_3.44.3         oligo_1.52.1
 [4] Biostrings_2.56.0    XVector_0.28.0       IRanges_2.22.2
 [7] S4Vectors_0.26.1     Biobase_2.48.0       oligoClasses_1.50.4
[10] BiocGenerics_0.34.0
loaded via a namespace (and not attached):
 [1] nlme_3.1-151                         bitops_1.0-6
 [3] matrixStats_0.57.0                   bit64_4.0.5
 [5] RColorBrewer_1.1-2                   httr_1.4.2
 [7] GenomeInfoDb_1.24.2                  Rgraphviz_2.32.0
 [9] tools_4.0.3                          doRNG_1.8.2
[11] R6_2.5.0                             affyio_1.58.0
[13] DBI_1.1.0                            colorspace_2.0-0
[15] gridExtra_2.3                        tidyselect_1.1.0
[17] bit_4.0.4                            compiler_4.0.3
[19] preprocessCore_1.50.0                graph_1.66.0
[21] SparseM_1.78                         DelayedArray_0.14.1
[23] KEGGgraph_1.48.0                     scales_1.1.1
[25] PADOG_1.30.0                         rappdirs_0.3.1
[27] stringr_1.4.0                        digest_0.6.27
[29] pkgconfig_2.0.3                      changepoint_2.2.2
[31] fgsea_1.14.0                         rlang_0.4.9
[33] RSQLite_2.2.1                        generics_0.1.0
[35] zoo_1.8-8                            BiocParallel_1.22.0
[37] dplyr_1.0.2                          ROntoTools_2.16.0
[39] RCurl_1.98-1.2                       magrittr_2.0.1
[41] GO.db_3.11.4                         GenomeInfoDbData_1.2.3
[43] Matrix_1.4-0                         Rcpp_1.0.5
[45] munsell_0.5.0                        EnrichmentBrowser_2.18.2
[47] lifecycle_0.2.0                      stringi_1.5.3
[49] pROC_1.16.2                          SummarizedExperiment_1.18.2
[51] zlibbioc_1.34.0                      plyr_1.8.6
[53] KEGGdzPathwaysGEO_1.26.0             grid_4.0.3
[55] affxparser_1.60.0                    blob_1.2.1
[57] crayon_1.3.4                         lattice_0.20-45
[59] splines_4.0.3                        annotate_1.66.0
[61] KEGGREST_1.28.0                      magick_2.5.2
[63] pillar_1.4.7                         rngtools_1.5
[65] igraph_1.2.6                         GenomicRanges_1.40.0
[67] SPIA_2.40.0                          hgu133plus2.db_3.2.3
[69] boot_1.3-28                          hgu133a.db_3.2.3
[71] KEGGandMetacoreDzPathwaysGEO_1.8.0   codetools_0.2-18
[73] fastmatch_1.1-0                      XML_3.99-0.5
[75] glue_1.4.2                           GSA_1.03.1
[77] data.table_1.13.4                    BiocManager_1.30.10
[79] png_0.1-7                            vctrs_0.3.6
[81] foreach_1.5.1                        org.Mm.eg.db_3.11.4
[83] purrr_0.3.4                          GSEABase_1.50.1
[85] gtable_0.3.0                         ggplot2_3.3.2
[87] xtable_1.8-4                         ff_4.0.5
[89] tibble_3.0.4                         iterators_1.0.13
[91] safe_3.28.1                          AnnotationDbi_1.50.3
[93] memoise_1.1.0                        ellipsis_0.3.1
```

FIGURE S1 Example environment configuration required by the PathwayKO platform in this study. (**A**) The initial status before loading any assigned component. (**B**) The updating status while loading relevant assigned components. (**C**) The final status after having installed all components required by the PathwayKO platform.

A

```
ayc@MMBL-WS:~$ cd /home/ayc/PathwayKO_platform
ayc@MMBL-WS:~/PathwayKO_platform$ ls
GSE22873_MA_preprocess_output   GSE24327_A_preprocess_output   GSE24327_C_preprocess_output
GSE22873_M_preprocess_output    GSE24327_B_preprocess_output   KEGG mmuSPIA.RData
ayc@MMBL-WS:~/PathwayKO_platform$ cd GSE22873_MA_preprocess_output/
ayc@MMBL-WS:~/PathwayKO_platform/GSE22873_MA_preprocess_output$ ls
GSE22873_MA_Myd88_Ager_RMA_PREP.RData   GSE22873_MA_pData.csv
ayc@MMBL-WS:~/PathwayKO_platform/GSE22873_MA_preprocess_output$ cd ..
ayc@MMBL-WS:~/PathwayKO_platform$ cd GSE22873_M_preprocess_output/
ayc@MMBL-WS:~/PathwayKO_platform/GSE22873_M_preprocess_output$ ls
GSE22873_M_Myd88_RMA_PREP.RData   GSE22873_M_pData.csv
ayc@MMBL-WS:~/PathwayKO_platform/GSE22873_M_preprocess_output$ cd ..
ayc@MMBL-WS:~/PathwayKO_platform$ cd GSE24327_A_preprocess_output/
ayc@MMBL-WS:~/PathwayKO_platform/GSE24327_A_preprocess_output$ ls
GSE24327_A_Myd88_RMA_PREP.RData   GSE24327_A_pData.csv
ayc@MMBL-WS:~/PathwayKO_platform/GSE24327_A_preprocess_output$ cd ..
ayc@MMBL-WS:~/PathwayKO_platform$ cd GSE24327_B_preprocess_output/
ayc@MMBL-WS:~/PathwayKO_platform/GSE24327_B_preprocess_output$ ls
GSE24327_B_Myd88_RMA_PREP.RData   GSE24327_B_pData.csv
ayc@MMBL-WS:~/PathwayKO_platform/GSE24327_B_preprocess_output$ cd ..
ayc@MMBL-WS:~/PathwayKO_platform$ cd GSE24327_C_preprocess_output/
ayc@MMBL-WS:~/PathwayKO_platform/GSE24327_C_preprocess_output$ ls
GSE24327_C_Myd88_RMA_PREP.RData   GSE24327_C_pData.csv
ayc@MMBL-WS:~/PathwayKO_platform/GSE24327_C_preprocess_output$ cd ..
```

B

```
ayc@MMBL-WS:~/PathwayKO_platform$ cd KEGG
ayc@MMBL-WS:~/PathwayKO_platform/KEGG$ ls
mmu00010.xml  mmu00512.xml  mmu00982.xml  mmu04066.xml  mmu04392.xml  mmu04730.xml  mmu04973.xml  mmu05200.xml
mmu00020.xml  mmu00513.xml  mmu00983.xml  mmu04068.xml  mmu04510.xml  mmu04740.xml  mmu04974.xml  mmu05202.xml
mmu00030.xml  mmu00514.xml  mmu01040.xml  mmu04070.xml  mmu04512.xml  mmu04742.xml  mmu04975.xml  mmu05203.xml
mmu00040.xml  mmu00515.xml  mmu01100.xml  mmu04071.xml  mmu04514.xml  mmu04744.xml  mmu04976.xml  mmu05204.xml
mmu00051.xml  mmu00520.xml  mmu01200.xml  mmu04072.xml  mmu04520.xml  mmu04750.xml  mmu04977.xml  mmu05205.xml
mmu00052.xml  mmu00524.xml  mmu01210.xml  mmu04080.xml  mmu04530.xml  mmu04810.xml  mmu04978.xml  mmu05206.xml
mmu00053.xml  mmu00531.xml  mmu01212.xml  mmu04110.xml  mmu04540.xml  mmu04910.xml  mmu04979.xml  mmu05210.xml
mmu00061.xml  mmu00532.xml  mmu01230.xml  mmu04114.xml  mmu04550.xml  mmu04911.xml  mmu05010.xml  mmu05211.xml
mmu00062.xml  mmu00533.xml  mmu01521.xml  mmu04115.xml  mmu04610.xml  mmu04912.xml  mmu05012.xml  mmu05212.xml
mmu00071.xml  mmu00534.xml  mmu01522.xml  mmu04120.xml  mmu04611.xml  mmu04913.xml  mmu05014.xml  mmu05213.xml
mmu00072.xml  mmu00561.xml  mmu01523.xml  mmu04122.xml  mmu04612.xml  mmu04914.xml  mmu05016.xml  mmu05214.xml
mmu00100.xml  mmu00562.xml  mmu01524.xml  mmu04130.xml  mmu04614.xml  mmu04915.xml  mmu05017.xml  mmu05215.xml
mmu00120.xml  mmu00563.xml  mmu02010.xml  mmu04136.xml  mmu04620.xml  mmu04916.xml  mmu05020.xml  mmu05216.xml
mmu00130.xml  mmu00564.xml  mmu03008.xml  mmu04137.xml  mmu04621.xml  mmu04917.xml  mmu05030.xml  mmu05217.xml
mmu00140.xml  mmu00565.xml  mmu03010.xml  mmu04140.xml  mmu04622.xml  mmu04918.xml  mmu05031.xml  mmu05218.xml
mmu00190.xml  mmu00590.xml  mmu03013.xml  mmu04141.xml  mmu04623.xml  mmu04919.xml  mmu05032.xml  mmu05219.xml
mmu00220.xml  mmu00591.xml  mmu03015.xml  mmu04142.xml  mmu04625.xml  mmu04920.xml  mmu05033.xml  mmu05220.xml
mmu00230.xml  mmu00592.xml  mmu03018.xml  mmu04144.xml  mmu04630.xml  mmu04921.xml  mmu05034.xml  mmu05221.xml
mmu00232.xml  mmu00600.xml  mmu03020.xml  mmu04145.xml  mmu04640.xml  mmu04922.xml  mmu05100.xml  mmu05222.xml
mmu00240.xml  mmu00601.xml  mmu03022.xml  mmu04146.xml  mmu04650.xml  mmu04923.xml  mmu05132.xml  mmu05223.xml
mmu00250.xml  mmu00603.xml  mmu03030.xml  mmu04150.xml  mmu04657.xml  mmu04924.xml  mmu05133.xml  mmu05224.xml
mmu00260.xml  mmu00604.xml  mmu03040.xml  mmu04151.xml  mmu04658.xml  mmu04925.xml  mmu05134.xml  mmu05225.xml
mmu00270.xml  mmu00620.xml  mmu03050.xml  mmu04152.xml  mmu04659.xml  mmu04926.xml  mmu05135.xml  mmu05226.xml
mmu00280.xml  mmu00630.xml  mmu03060.xml  mmu04210.xml  mmu04660.xml  mmu04927.xml  mmu05140.xml  mmu05230.xml
mmu00290.xml  mmu00640.xml  mmu03320.xml  mmu04211.xml  mmu04662.xml  mmu04928.xml  mmu05142.xml  mmu05231.xml
mmu00310.xml  mmu00650.xml  mmu03410.xml  mmu04213.xml  mmu04664.xml  mmu04929.xml  mmu05143.xml  mmu05235.xml
mmu00330.xml  mmu00670.xml  mmu03420.xml  mmu04215.xml  mmu04666.xml  mmu04930.xml  mmu05144.xml  mmu05310.xml
mmu00340.xml  mmu00730.xml  mmu03430.xml  mmu04216.xml  mmu04668.xml  mmu04931.xml  mmu05145.xml  mmu05320.xml
mmu00350.xml  mmu00740.xml  mmu03440.xml  mmu04217.xml  mmu04670.xml  mmu04932.xml  mmu05146.xml  mmu05321.xml
mmu00360.xml  mmu00750.xml  mmu03450.xml  mmu04218.xml  mmu04672.xml  mmu04933.xml  mmu05150.xml  mmu05322.xml
mmu00380.xml  mmu00760.xml  mmu03460.xml  mmu04260.xml  mmu04710.xml  mmu04934.xml  mmu05152.xml  mmu05323.xml
mmu00400.xml  mmu00770.xml  mmu04010.xml  mmu04261.xml  mmu04713.xml  mmu04935.xml  mmu05160.xml  mmu05330.xml
mmu00410.xml  mmu00780.xml  mmu04012.xml  mmu04270.xml  mmu04714.xml  mmu04940.xml  mmu05161.xml  mmu05332.xml
mmu00430.xml  mmu00785.xml  mmu04014.xml  mmu04310.xml  mmu04720.xml  mmu04950.xml  mmu05162.xml  mmu05340.xml
mmu00440.xml  mmu00790.xml  mmu04015.xml  mmu04330.xml  mmu04721.xml  mmu04960.xml  mmu05163.xml  mmu05410.xml
mmu00450.xml  mmu00830.xml  mmu04020.xml  mmu04340.xml  mmu04722.xml  mmu04961.xml  mmu05164.xml  mmu05412.xml
mmu00471.xml  mmu00860.xml  mmu04022.xml  mmu04350.xml  mmu04723.xml  mmu04962.xml  mmu05165.xml  mmu05414.xml
mmu00472.xml  mmu00900.xml  mmu04024.xml  mmu04360.xml  mmu04724.xml  mmu04964.xml  mmu05166.xml  mmu05416.xml
mmu00480.xml  mmu00910.xml  mmu04060.xml  mmu04370.xml  mmu04725.xml  mmu04966.xml  mmu05167.xml  mmu05418.xml
mmu00500.xml  mmu00920.xml  mmu04061.xml  mmu04371.xml  mmu04726.xml  mmu04970.xml  mmu05168.xml
mmu00510.xml  mmu00970.xml  mmu04062.xml  mmu04380.xml  mmu04727.xml  mmu04971.xml  mmu05169.xml
mmu00511.xml  mmu00980.xml  mmu04064.xml  mmu04390.xml  mmu04728.xml  mmu04972.xml  mmu05170.xml
ayc@MMBL-WS:~/PathwayKO_platform/KEGG$ cd ..
```

FIGURE S2 Snapshots of the complete list of files required for a batch-computation by the `Pathwayko_batch` module in this study. (**A**) The benchmark containing the preprocessed five data, and the preprocessed mmuSPIA.RData. (**B**) The KEGG signaling pathways used to generate mmuSPIA.RData.

A

```
ayc@MMBL-WS:~$ cd /home/ayc/PathwayKO_platform
ayc@MMBL-WS:~/PathwayKO_platform$ ls
GSE22873_MA_preprocess_output   GSE24327_A_preprocess_output   GSE24327_C_preprocess_output
GSE22873_M_preprocess_output    GSE24327_B_preprocess_output   KEGG mmuSPIA.RData

PathwayKO_platform$ R
> library(oligo)
> library(limma)
> library(pathwayko)
> pathwayko_batch()

INFO: Acquiring user parameters...
INFO: files in current working directory:
[1] "/home/ayc/PathwayKO_platform/GSE22873_M_preprocess_output/GSE22873_M_Myd88_RMA_PREP.RData"
[2] "/home/ayc/PathwayKO_platform/GSE22873_MA_preprocess_output/GSE22873_MA_Myd88_Ager_RMA_PREP.RData"
[3] "/home/ayc/PathwayKO_platform/GSE24327_A_preprocess_output/GSE24327_A_Myd88_RMA_PREP.RData"
[4] "/home/ayc/PathwayKO_platform/GSE24327_B_preprocess_output/GSE24327_B_Myd88_RMA_PREP.RData"
[5] "/home/ayc/PathwayKO_platform/GSE24327_C_preprocess_output/GSE24327_C_Myd88_RMA_PREP.RData"
Process all?: (Y/N) Y

INFO: successfully queued 5 RData files.
INFO: directories in current working directory:
[1] "/home/ayc/PathwayKO_platform"
[2] "/home/ayc/PathwayKO_platform/GSE22873_M_preprocess_output"
[3] "/home/ayc/PathwayKO_platform/GSE22873_MA_preprocess_output"
[4] "/home/ayc/PathwayKO_platform/GSE24327_A_preprocess_output"
[5] "/home/ayc/PathwayKO_platform/GSE24327_B_preprocess_output"
[6] "/home/ayc/PathwayKO_platform/GSE24327_C_preprocess_output"
[7] "/home/ayc/PathwayKO_platform/KEGG"
Enter KEGG xml directory index: 7

INFO: SPIA data in current working directory:
[1] "/home/ayc/PathwayKO_platform/mmuSPIA.RData"
Enter SPIA data index: 1

Run SPIA?: (Y/N) Y
Run ROntoTools PE?: (Y/N) Y
Run ROntoTools PDIS?: (Y/N) Y
Run PADOG?: (Y/N) Y
Run GSA?: (Y/N) Y
Run SAFE?: (Y/N) Y
Run GSEA?: (Y/N) Y

How to choose DE genes? ('HES1','HES2','HES3' for HighEdgeS lower, change point, upper bound, 'CLA' for Classical)
Note: geneset-based methods will not use DE genes regardlessly: HES1
Lower bound of specificity (X-axis) in partial ROC? (e.g. 90): 90
Lower bound of sensitivity (Y-axis) in partial ROC? (e.g. 90): 90
```

B

```
INFO: batch job ( 1 / 5 )...
INFO: starting pathwayko...
INFO: preprocessing...
INFO: 122 genes are considered differentially expressed.
INFO: target pathways:
 [1] "04010" "04064" "04620" "04621" "05132" "05133" "05134" "05135" "05140"
[10] "05142" "05143" "05144" "05145" "05152" "05161" "05162" "05164" "05168"
[19] "05169" "05170" "05235"
INFO: pathway analysis...
INFO: plotting ROC...
INFO: pathwayko completed.
INFO: batch job ( 1 / 5 ) completed.

INFO: batch job ( 2 / 5 )...
INFO: starting pathwayko...
INFO: preprocessing...
INFO: 130 genes are considered differentially expressed.
INFO: target pathways:
 [1] "04010" "04064" "04620" "04621" "04933" "05010" "05132" "05133" "05134"
[10] "05135" "05140" "05142" "05143" "05144" "05145" "05150" "05152" "05161"
[19] "05162" "05164" "05168" "05169" "05170" "05235"
INFO: pathway analysis...
INFO: plotting ROC...
INFO: pathwayko completed.
INFO: batch job ( 2 / 5 ) completed.
```

```
INFO: batch job ( 3 / 5 )...
INFO: starting pathwayko...
INFO: preprocessing...
INFO: 408 genes are considered differentially expressed.
INFO: target pathways:
  [1] "04010" "04064" "04620" "04621" "05132" "05133" "05134" "05135" "05140"
[10] "05142" "05143" "05144" "05145" "05152" "05161" "05162" "05164" "05168"
[19] "05169" "05170" "05235"
INFO: pathway analysis...
INFO: plotting ROC...
INFO: pathwayko completed.
INFO: batch job ( 3 / 5 ) completed.

INFO: batch job ( 4 / 5 )...
INFO: starting pathwayko...
INFO: preprocessing...
INFO: 266 genes are considered differentially expressed.
INFO: target pathways:
  [1] "04010" "04064" "04620" "04621" "05132" "05133" "05134" "05135" "05140"
[10] "05142" "05143" "05144" "05145" "05152" "05161" "05162" "05164" "05168"
[19] "05169" "05170" "05235"
INFO: pathway analysis...
INFO: plotting ROC...
INFO: pathwayko completed.
INFO: batch job ( 4 / 5 ) completed.

INFO: batch job ( 5 / 5 )...
INFO: starting pathwayko...
INFO: preprocessing...
INFO: 648 genes are considered differentially expressed.
INFO: target pathways:
  [1] "04010" "04064" "04620" "04621" "05132" "05133" "05134" "05135" "05140"
[10] "05142" "05143" "05144" "05145" "05152" "05161" "05162" "05164" "05168"
[19] "05169" "05170" "05235"
INFO: pathway analysis...
INFO: plotting ROC...
INFO: pathwayko completed.
INFO: batch job ( 5 / 5 ) completed.

> combineresult()
INFO: files in current working directory:
[1] "/home/ayc/PathwayKO_platform/GSE22873_M_result_output/GSE22873_M_RMA_Myd88_HES1_SUM.RData"
[2] "/home/ayc/PathwayKO_platform/GSE22873_MA_result_output/GSE22873_MA_RMA_Myd88_Ager_HES1_SUM.RData"
[3] "/home/ayc/PathwayKO_platform/GSE24327_A_result_output/GSE24327_A_RMA_Myd88_HES1_SUM.RData"
[4] "/home/ayc/PathwayKO_platform/GSE24327_B_result_output/GSE24327_B_RMA_Myd88_HES1_SUM.RData"
[5] "/home/ayc/PathwayKO_platform/GSE24327_C_result_output/GSE24327_C_RMA_Myd88_HES1_SUM.RData"
Process all?: (Y/N) Y
INFO: successfully loaded 5 RData files.
INFO: combining results...
INFO: all process completed.

> violinplot()
INFO: summary files in current working directory:
[1] "/home/ayc/PathwayKO_platform/combinedresults/2023-01-06.STATS.RData"
Enter stats data index: 1
INFO: starting...
INFO: completed.

> wilcoxtest()
INFO: summary files in current working directory:
[1] "/home/ayc/PathwayKO_platform/combinedresults/2023-01-06.STATS.RData"
Enter stats data index: 1
INFO: starting...
INFO: completed.
```

C

FIGURE S3 Snapshots of a batch-computation by the `Pathwayko_batch` module in this study. (**A**) The environment configuration required for the batch-computation. (**B**) The sequential executions of the batch-computation across the five data. (**C**) The post-executions of statistics analyses based on the resulting outputs.

```
ayc@MMBL-WS:~$ cd /home/ayc/PathwayKO_platform
ayc@MMBL-WS:~/PathwayKO_platform$ ls                                                                                     A
combinedresults                 GSE22873_M_result_output          GSE24327_B_result_output          mmuSPIA.RData
GSE22873_MA_preprocess_output   GSE24327_A_preprocess_output   GSE24327_C_preprocess_output   pathwayko_batch_log.txt
GSE22873_MA_result_output       GSE24327_A_result_output          GSE24327_C_result_output          ViolinPlots
GSE22873_M_preprocess_output    GSE24327_B_preprocess_output   KEGG                                        Wilcoxtests

ayc@MMBL-WS:~/PathwayKO_platform$                                                                                         B
ayc@MMBL-WS:~/PathwayKO_platform$ cd GSE22873_MA_result_output/
ayc@MMBL-WS:~/PathwayKO_platform/GSE22873_MA_result_output$ ls
GSE22873_MA_RMA_Myd88_Ager_HES1_AUC.csv                         GSE22873_MA_RMA_Myd88_Ager_HES1_RONTOTOOLS_PDIS_CI.pdf
GSE22873_MA_RMA_Myd88_Ager_HES1_DE_genes.csv                    GSE22873_MA_RMA_Myd88_Ager_HES1_RONTOTOOLS_PDIS.csv
GSE22873_MA_RMA_Myd88_Ager_HES1_GSA_CI.pdf
GSE22873_MA_RMA_Myd88_Ager_HES1_RONTOTOOLS_PDIS_pAUC.pdf
GSE22873_MA_RMA_Myd88_Ager_HES1_GSA.csv
GSE22873_MA_RMA_Myd88_Ager_HES1_RONTOTOOLS_PDIS_ROC.pdf
GSE22873_MA_RMA_Myd88_Ager_HES1_GSA_pAUC.pdf                    GSE22873_MA_RMA_Myd88_Ager_HES1_RONTOTOOLS_PE_CI.pdf
GSE22873_MA_RMA_Myd88_Ager_HES1_GSA_ROC.pdf                     GSE22873_MA_RMA_Myd88_Ager_HES1_RONTOTOOLS_PE.csv
GSE22873_MA_RMA_Myd88_Ager_HES1_GSEA_CI.pdf                     GSE22873_MA_RMA_Myd88_Ager_HES1_RONTOTOOLS_PE_pAUC.pdf
GSE22873_MA_RMA_Myd88_Ager_HES1_GSEA.csv                        GSE22873_MA_RMA_Myd88_Ager_HES1_RONTOTOOLS_PE_ROC.pdf
GSE22873_MA_RMA_Myd88_Ager_HES1_GSEA_pAUC.pdf                   GSE22873_MA_RMA_Myd88_Ager_HES1_SAFE_CI.pdf
GSE22873_MA_RMA_Myd88_Ager_HES1_GSEA_ROC.pdf                    GSE22873_MA_RMA_Myd88_Ager_HES1_SAFE.csv
GSE22873_MA_RMA_Myd88_Ager_HES1_HighEdgeS_plot.pdf              GSE22873_MA_RMA_Myd88_Ager_HES1_SAFE_pAUC.pdf
GSE22873_MA_RMA_Myd88_Ager_HES1_Histogram_plot.pdf              GSE22873_MA_RMA_Myd88_Ager_HES1_SAFE_ROC.pdf
GSE22873_MA_RMA_Myd88_Ager_HES1_PADOG_CI.pdf                    GSE22873_MA_RMA_Myd88_Ager_HES1_SPIA_CI.pdf
GSE22873_MA_RMA_Myd88_Ager_HES1_PADOG.csv                       GSE22873_MA_RMA_Myd88_Ager_HES1_SPIA.csv
GSE22873_MA_RMA_Myd88_Ager_HES1_PADOG_pAUC.pdf                  GSE22873_MA_RMA_Myd88_Ager_HES1_SPIA_pAUC.pdf
GSE22873_MA_RMA_Myd88_Ager_HES1_PADOG_ROC.pdf                   GSE22873_MA_RMA_Myd88_Ager_HES1_SPIA_ROC.pdf
GSE22873_MA_RMA_Myd88_Ager_HES1_pAUC_SE_90_Corrected.csv      GSE22873_MA_RMA_Myd88_Ager_HES1_stats1.csv
GSE22873_MA_RMA_Myd88_Ager_HES1_pAUC_SE_90_Original.csv       GSE22873_MA_RMA_Myd88_Ager_HES1_stats2.csv
GSE22873_MA_RMA_Myd88_Ager_HES1_pAUC_SP_90_Corrected.csv      GSE22873_MA_RMA_Myd88_Ager_HES1_stats.csv
GSE22873_MA_RMA_Myd88_Ager_HES1_pAUC_SP_90_Original.csv       GSE22873_MA_RMA_Myd88_Ager_HES1_SUM.RData
GSE22873_MA_RMA_Myd88_Ager_HES1_ROC_combined.pdf                GSE22873_MA_RMA_Myd88_Ager_HES1_target_pathways.csv
ayc@MMBL-WS:~/PathwayKO_platform/GSE22873_MA_result_output$ cd ..

ayc@MMBL-WS:~/PathwayKO_platform$ ls
combinedresults                 GSE22873_M_result_output          GSE24327_B_result_output          mmuSPIA.RData    C
GSE22873_MA_preprocess_output   GSE24327_A_preprocess_output   GSE24327_C_preprocess_output   pathwayko_batch_log.txt
GSE22873_MA_result_output       GSE24327_A_result_output          GSE24327_C_result_output          ViolinPlots
GSE22873_M_preprocess_output    GSE24327_B_preprocess_output   KEGG                                        Wilcoxtests
ayc@MMBL-WS:~/PathwayKO_platform$ cd combinedresults/
ayc@MMBL-WS:~/PathwayKO_platform/combinedresults$ ls
2023-01-06.STATS.RData   Summary.AUC.csv   Summary.parameters.csv   Summary.pAUC_SE.csv   Summary.pAUC_SP.csv
ayc@MMBL-WS:~/PathwayKO_platform/combinedresults$ cd ..

ayc@MMBL-WS:~/PathwayKO_platform$ cd ViolinPlots/
ayc@MMBL-WS:~/PathwayKO_platform/ViolinPlots$ ls
plot.Accuracy.pdf   plot.FDR.pdf   plot.pAUC_SE.pdf      plot.Recall.pdf           plot.STATS1.comb.pdf   plot.STATS5.comb.pdf
plot.AUC.comb.pdf   plot.FNR.pdf   plot.pAUC_SP.pdf       plot.Sensitivity.pdf   plot.STATS2.comb.pdf   plot.Threshold.pdf
plot.AUC.pdf            plot.FPR.pdf   plot.Precision.pdf   plot.Specificity.pdf   plot.STATS3.comb.pdf
ayc@MMBL-WS:~/PathwayKO_platform/ViolinPlots$ cd ..

ayc@MMBL-WS:~/PathwayKO_platform$ cd Wilcoxtests/
ayc@MMBL-WS:~/PathwayKO_platform/Wilcoxtests$ ls
wilcox.out.txt
ayc@MMBL-WS:~/PathwayKO_platform/Wilcoxtests$ cd ..
ayc@MMBL-WS:~/PathwayKO_platform$ cd ..
```

FIGURE S4 Snapshots of a batch-computation by the `Pathwayko_batch` module in this study. (**A**) The directories of resulting outputs after the batch-computation. (**B**) The complete list of resulting output files in GSE22873_MA_result_output, as an example, after the batch-computation. (**C**) The complete list of resulting output files after the post-statistics analyses.

Table_S1 GSE22873_M (a. KO *Myd88 vs.* WT)_RMA_Myd88_HES1_DE_genes

	ENTREZID	SYMBOL	GENENAME
1	17874	***Myd88***	myeloid differentiation primary response gene 88
2	14825	***Cxcl1***	chemokine (C-X-C motif) ligand 1
3	14281	*Fos*	FBJ osteosarcoma oncogene
4	18021	*Nfatc3*	nuclear factor of activated T cells, cytoplasmic, calcineurin dependent 3
5	12770	*Ccr1l1*	chemokine (C-C motif) receptor 1-like 1
6	12765	*Cxcr2*	chemokine (C-X-C motif) receptor 2
7	12773	*Ccr4*	chemokine (C-C motif) receptor 4
8	19697	*Rela*	v-rel reticuloendotheliosis viral oncogene homolog A (avian)
9	16476	*Jun*	jun proto-oncogene
10	23832	*Xcr1*	chemokine (C motif) receptor 1
11	73181	*Nfatc4*	nuclear factor of activated T cells, cytoplasmic, calcineurin dependent 4
12	12768	*Ccr1*	chemokine (C-C motif) receptor 1
13	80901	*Cxcr6*	chemokine (C-X-C motif) receptor 6
14	12766	*Cxcr3*	chemokine (C-X-C motif) receptor 3
15	12777	*Ccr10*	chemokine (C-C motif) receptor 10
16	15251	*Hif1a*	hypoxia inducible factor 1, alpha subunit
17	18019	*Nfatc2*	nuclear factor of activated T cells, cytoplasmic, calcineurin dependent 2
18	12769	*Ccr9*	chemokine (C-C motif) receptor 9
19	12774	*Ccr5*	chemokine (C-C motif) receptor 5
20	12776	*Ccr8*	chemokine (C-C motif) receptor 8
21	13051	*Cx3cr1*	chemokine (C-X3-C motif) receptor 1
22	12458	*Ccr6*	chemokine (C-C motif) receptor 6
23	18018	*Nfatc1*	nuclear factor of activated T cells, cytoplasmic, calcineurin dependent 1
24	18033	*Nfkb1*	nuclear factor of kappa light polypeptide gene enhancer in B cells 1, p105
25	12145	*Cxcr5*	chemokine (C-X-C motif) receptor 5
26	12772	*Ccr2*	chemokine (C-C motif) receptor 2
27	12775	*Ccr7*	chemokine (C-C motif) receptor 7
28	12771	*Ccr3*	chemokine (C-C motif) receptor 3
29	12767	*Cxcr4*	chemokine (C-X-C motif) receptor 4
30	66724	*Tab3*	TGF-beta activated kinase 1/MAP3K7 binding protein 3
31	66513	*Tab1*	TGF-beta activated kinase 1/MAP3K7 binding protein 1
32	18126	*Nos2*	nitric oxide synthase 2, inducible
33	68652	*Tab2*	TGF-beta activated kinase 1/MAP3K7 binding protein 2
34	16159	*Il12a*	interleukin 12a
35	20310	*Cxcl2*	chemokine (C-X-C motif) ligand 2
36	17342	*Mitf*	melanogenesis associated transcription factor
37	13190	*Dct*	dopachrome tautomerase
38	21926	*Tnf*	tumor necrosis factor
39	24088	*Tlr2*	toll-like receptor 2
40	15977	*Ifnb1*	interferon beta 1, fibroblast
41	22337	*Vdr*	vitamin D (1,25-dihydroxyvitamin D3) receptor
42	170743	*Tlr7*	toll-like receptor 7
43	20847	*Stat2*	signal transducer and activator of transcription 2
44	16177	*Il1r1*	interleukin 1 receptor, type I
45	81897	*Tlr9*	toll-like receptor 9
46	16179	*Irak1*	interleukin-1 receptor-associated kinase 1
47	21898	*Tlr4*	toll-like receptor 4
48	14082	*Fadd*	Fas (TNFRSF6)-associated via death domain
49	16180	*Il1rap*	interleukin 1 receptor accessory protein
50	15967	*Ifna4*	interferon alpha 4
51	21899	*Tlr6*	toll-like receptor 6
52	16193	*Il6*	interleukin 6
53	21897	*Tlr1*	toll-like receptor 1
54	26409	*Map3k7*	mitogen-activated protein kinase kinase kinase 7
55	20846	*Stat1*	signal transducer and activator of transcription 1
56	330122	*Cxcl3*	chemokine (C-X-C motif) ligand 3
57	15972	*Ifna9*	interferon alpha 9
58	22034	*Traf6*	TNF receptor-associated factor 6
59	266632	*Irak4*	interleukin-1 receptor-associated kinase 4
60	16176	*Il1b*	interleukin 1 beta
61	17087	*Ly96*	lymphocyte antigen 96

62	13115	*Cyp27b1*	cytochrome P450, family 27, subfamily b, polypeptide 1
63	16160	*Il12b*	interleukin 12b
64	15968	*Ifna5*	interferon alpha 5
65	170744	*Tlr8*	toll-like receptor 8
66	15974	*Ifnab*	interferon alpha B
67	55985	*Cxcl13*	chemokine (C-X-C motif) ligand 13
68	11835	*Ar*	androgen receptor
69	19013	*Ppara*	peroxisome proliferator activated receptor alpha
70	17869	*Myc*	myelocytomatosis oncogene
71	14961	*H2-Ab1*	histocompatibility 2, class II antigen A, beta 1
72	14969	*H2-Eb1*	histocompatibility 2, class II antigen E beta
73	208647	*Creb3l2*	cAMP responsive element binding protein 3-like 2
74	14282	*Fosb*	FBJ osteosarcoma oncogene B
75	14960	*H2-Aa*	histocompatibility 2, class II antigen A, alpha
76	16480	*Jup*	junction plakoglobin
77	235320	*Zbtb16*	zinc finger and BTB domain containing 16
78	11911	*Atf4*	activating transcription factor 4
79	13038	*Ctsk*	cathepsin K
80	16149	*Cd74*	CD74 antigen (invariant polypeptide of major histocompatibility complex, class II antigen-associated)
81	23872	*Ets2*	E26 avian leukemia oncogene 2, 3' domain
82	13653	*Egr1*	early growth response 1
83	15182	*Hdac2*	histone deacetylase 2
84	208715	*Hmgcs1*	3-hydroxy-3-methylglutaryl-Coenzyme A synthase 1
85	14733	*Gpc1*	glypican 1
86	12389	*Cav1*	caveolin 1, caveolae protein
87	20239	*Atxn2*	ataxin 2
88	16438	*Itpr1*	inositol 1,4,5-trisphosphate receptor 1
89	20181	*Rxra*	retinoid X receptor alpha
90	15945	*Cxcl10*	chemokine (C-X-C motif) ligand 10
91	207742	*Rnf43*	ring finger protein 43
92	14370	*Fzd8*	frizzled class receptor 8
93	18747	*Prkaca*	protein kinase, cAMP dependent, catalytic, alpha
94	21416	*Tcf7l2*	transcription factor 7 like 2, T cell specific, HMG box
95	26415	*Mapk13*	mitogen-activated protein kinase 13
96	20304	*Ccl5*	chemokine (C-C motif) ligand 5
97	109264	*Me3*	malic enzyme 3, NADP(+)-dependent, mitochondrial
98	11909	*Atf2*	activating transcription factor 2
99	13122	*Cyp7a1*	cytochrome P450, family 7, subfamily a, polypeptide 1
100	13119	*Cyp4a14*	cytochrome P450, family 4, subfamily a, polypeptide 14
101	15360	*Hmgcs2*	3-hydroxy-3-methylglutaryl-Coenzyme A synthase 2
102	13867	*Erbb3*	erb-b2 receptor tyrosine kinase 3
103	18754	*Prkce*	protein kinase C, epsilon
104	384783	*Irs2*	insulin receptor substrate 2
105	57875	*Angptl4*	angiopoietin-like 4
106	74205	*Acsl3*	acyl-CoA synthetase long-chain family member 3
107	14367	*Fzd5*	frizzled class receptor 5
108	54123	*Irf7*	interferon regulatory factor 7
109	26427	*Creb3l1*	cAMP responsive element binding protein 3-like 1
110	12606	*Cebpa*	CCAAT/enhancer binding protein (C/EBP), alpha
111	12568	*Cdk5*	cyclin-dependent kinase 5
112	22340	*Vegfb*	vascular endothelial growth factor B
113	17130	*Smad6*	SMAD family member 6
114	17125	*Smad1*	SMAD family member 1
115	20296	*Ccl2*	chemokine (C-C motif) ligand 2
116	17436	*Me1*	malic enzyme 1, NADP(+)-dependent, cytosolic
117	14784	*Grb2*	growth factor receptor bound protein 2
118	12915	*Atf6b*	activating transcription factor 6 beta
119	14366	*Fzd4*	frizzled class receptor 4
120	17977	*Ncoa1*	nuclear receptor coactivator 1
121	18045	*Nfyb*	nuclear transcription factor-Y beta
122	56637	*Gsk3b*	glycogen synthase kinase 3 beta

* HES1: 122 DEGs; HES2: 38 DEGs above the second line. HES3: 2 DEGs above the first line. *Myd88* and *Cxcl1* are the highest top-ranked.

Table_S2 GSE22873_MA (b. KO *Myd88_Ager vs.* WT)_RMA_Myd88_Ager_HES1_DE_genes

	ENTREZID	SYMBOL	GENENAME
1	17874	***Myd88***	myeloid differentiation primary response gene 88
2	14825	***Cxcl1***	chemokine (C-X-C motif) ligand 1
3	14963	*H2-Bl*	histocompatibility 2, blastocyst
4	12770	*Ccr1l1*	chemokine (C-C motif) receptor 1-like 1
5	12777	*Ccr10*	chemokine (C-C motif) receptor 10
6	12765	*Cxcr2*	chemokine (C-X-C motif) receptor 2
7	19697	*Rela*	v-rel reticuloendotheliosis viral oncogene homolog A (avian)
8	23832	*Xcr1*	chemokine (C motif) receptor 1
9	12768	*Ccr1*	chemokine (C-C motif) receptor 1
10	12145	*Cxcr5*	chemokine (C-X-C motif) receptor 5
11	12766	*Cxcr3*	chemokine (C-X-C motif) receptor 3
12	73181	*Nfatc4*	nuclear factor of activated T cells, cytoplasmic, calcineurin dependent 4
13	12776	*Ccr8*	chemokine (C-C motif) receptor 8
14	18019	*Nfatc2*	nuclear factor of activated T cells, cytoplasmic, calcineurin dependent 2
15	18021	*Nfatc3*	nuclear factor of activated T cells, cytoplasmic, calcineurin dependent 3
16	12771	*Ccr3*	chemokine (C-C motif) receptor 3
17	16476	*Jun*	jun proto-oncogene
18	80901	*Cxcr6*	chemokine (C-X-C motif) receptor 6
19	12772	*Ccr2*	chemokine (C-C motif) receptor 2
20	12773	*Ccr4*	chemokine (C-C motif) receptor 4
21	13051	*Cx3cr1*	chemokine (C-X3-C motif) receptor 1
22	18033	*Nfkb1*	nuclear factor of kappa light polypeptide gene enhancer in B cells 1, p105
23	12775	*Ccr7*	chemokine (C-C motif) receptor 7
24	15251	*Hif1a*	hypoxia inducible factor 1, alpha subunit
25	14281	*Fos*	FBJ osteosarcoma oncogene
26	12767	*Cxcr4*	chemokine (C-X-C motif) receptor 4
27	12769	*Ccr9*	chemokine (C-C motif) receptor 9
28	12458	*Ccr6*	chemokine (C-C motif) receptor 6
29	12774	*Ccr5*	chemokine (C-C motif) receptor 5
30	18018	*Nfatc1*	nuclear factor of activated T cells, cytoplasmic, calcineurin dependent 1
31	15042	*H2-T24*	histocompatibility 2, T region locus 24
32	110557	*H2-Q6*	histocompatibility 2, Q region locus 6
33	14991	*H2-M3*	histocompatibility 2, M region locus 3
34	11767	*Ap1m1*	adaptor-related protein complex AP-1, mu subunit 1
35	12575	*Cdkn1a*	cyclin-dependent kinase inhibitor 1A (P21)
36	11766	*Ap1g2*	adaptor protein complex AP-1, gamma 2 subunit
37	11768	*Ap1m2*	adaptor protein complex AP-1, mu 2 subunit
38	21356	*Tapbp*	TAP binding protein
39	12525	*Cd8a*	CD8 antigen, alpha chain
40	11764	*Ap1b1*	adaptor protein complex AP-1, beta 1 subunit
41	15043	*H2-T3*	histocompatibility 2, T region locus 3
42	108012	*Ap1s2*	adaptor-related protein complex 1, sigma 2 subunit
43	12526	*Cd8b1*	CD8 antigen, beta chain 1
44	16641	*Klrc1*	killer cell lectin-like receptor subfamily C, member 1
45	14972	*H2-K1*	histocompatibility 2, K1, K region
46	15007	*H2-Q10*	histocompatibility 2, Q region locus 10
47	14827	*Pdia3*	protein disulfide isomerase associated 3
48	12317	*Calr*	calreticulin
49	11765	*Ap1g1*	adaptor protein complex AP-1, gamma 1 subunit
50	12010	*B2m*	beta-2 microglobulin
51	16643	*Klrd1*	killer cell lectin-like receptor, subfamily D, member 1
52	11769	*Ap1s1*	adaptor protein complex AP-1, sigma 1
53	14997	*H2-M9*	histocompatibility 2, M region locus 9
54	14985	*H2-M10.1*	histocompatibility 2, M region locus 10.1
55	15013	*H2-Q2*	histocompatibility 2, Q region locus 2
56	14964	*H2-D1*	histocompatibility 2, D region locus 1
57	20310	*Cxcl2*	chemokine (C-X-C motif) ligand 2
58	16177	*Il1r1*	interleukin 1 receptor, type I
59	14082	*Fadd*	Fas (TNFRSF6)-associated via death domain
60	66724	*Tab3*	TGF-beta activated kinase 1/MAP3K7 binding protein 3

61	18126	*Nos2*	nitric oxide synthase 2, inducible
62	170744	*Tlr8*	toll-like receptor 8
63	66513	*Tab1*	TGF-beta activated kinase 1/MAP3K7 binding protein 1
64	22034	*Traf6*	TNF receptor-associated factor 6
65	20847	*Stat2*	signal transducer and activator of transcription 2
66	16159	*Il12a*	interleukin 12a
67	13115	*Cyp27b1*	cytochrome P450, family 27, subfamily b, polypeptide 1
68	21897	*Tlr1*	toll-like receptor 1
69	21899	*Tlr6*	toll-like receptor 6
70	22337	*Vdr*	vitamin D (1,25-dihydroxyvitamin D3) receptor
71	16179	*Irak1*	interleukin-1 receptor-associated kinase 1
72	330122	*Cxcl3*	chemokine (C-X-C motif) ligand 3
73	68652	*Tab2*	TGF-beta activated kinase 1/MAP3K7 binding protein 2
74	81897	*Tlr9*	toll-like receptor 9
75	16193	*Il6*	interleukin 6
76	170743	*Tlr7*	toll-like receptor 7
77	15977	*Ifnb1*	interferon beta 1, fibroblast
78	15945	*Cxcl10*	chemokine (C-X-C motif) ligand 10
79	17087	*Ly96*	lymphocyte antigen 96
80	16180	*Il1rap*	interleukin 1 receptor accessory protein
81	21926	*Tnf*	tumor necrosis factor
82	24088	*Tlr2*	toll-like receptor 2
83	15967	*Ifna4*	interferon alpha 4
84	266632	*Irak4*	interleukin-1 receptor-associated kinase 4
85	16176	*Il1b*	interleukin 1 beta
86	20846	*Stat1*	signal transducer and activator of transcription 1
87	15968	*Ifna5*	interferon alpha 5
88	16160	*Il12b*	interleukin 12b
89	15972	*Ifna9*	interferon alpha 9
90	26409	*Map3k7*	mitogen-activated protein kinase kinase kinase 7
91	21898	*Tlr4*	toll-like receptor 4
92	15974	*Ifnab*	interferon alpha B
93	54131	*Irf3*	interferon regulatory factor 3
94	54123	*Irf7*	interferon regulatory factor 7
95	22637	*Zap70*	zeta-chain (TCR) associated protein kinase
96	18708	*Pik3r1*	phosphoinositide-3-kinase regulatory subunit 1
97	16391	*Irf9*	interferon regulatory factor 9
98	29857	*Mapk12*	mitogen-activated protein kinase 12
99	80859	*Nfkbiz*	nuclear factor of kappa light polypeptide gene enhancer in B cells inhibitor, zeta
100	20296	*Ccl2*	chemokine (C-C motif) ligand 2
101	12737	*Cldn1*	claudin 1
102	27375	*Tjp3*	tight junction protein 3
103	17342	*Mitf*	melanogenesis associated transcription factor
104	13190	*Dct*	dopachrome tautomerase
105	11652	*Akt2*	thymoma viral proto-oncogene 2
106	55985	*Cxcl13*	chemokine (C-X-C motif) ligand 13
107	18709	*Pik3r2*	phosphoinositide-3-kinase regulatory subunit 2
108	14360	*Fyn*	Fyn proto-oncogene
109	106759	*Ticam1*	toll-like receptor adaptor molecule 1
110	68458	*Ppp1r14a*	protein phosphatase 1, regulatory inhibitor subunit 14A
111	232807	*Ppp1r12c*	protein phosphatase 1, regulatory subunit 12C
112	16818	*Lck*	lymphocyte protein tyrosine kinase
113	11651	*Akt1*	thymoma viral proto-oncogene 1
114	26416	*Mapk14*	mitogen-activated protein kinase 14
115	15170	*Ptpn6*	protein tyrosine phosphatase, non-receptor type 6
116	12501	*Cd3e*	CD3 antigen, epsilon polypeptide
117	26415	*Mapk13*	mitogen-activated protein kinase 13
118	18710	*Pik3r3*	phosphoinositide-3-kinase regulatory subunit 3
119	12739	*Cldn3*	claudin 3
120	12503	*Cd247*	CD247 antigen
121	29806	*Limd1*	LIM domains containing 1
122	64010	*Sav1*	salvador family WW domain containing 1
123	50523	*Lats2*	large tumor suppressor 2
124	208650	*Cblb*	Casitas B-lineage lymphoma b

125	216148	*Shc2*	SHC (Src homology 2 domain containing) transforming protein 2
126	16798	*Lats1*	large tumor suppressor
127	12502	*Cd3g*	CD3 antigen, gamma polypeptide
128	16822	*Lcp2*	lymphocyte cytosolic protein 2
129	16797	*Lat*	linker for activation of T cells
130	74769	*Pik3cb*	phosphatidylinositol-4,5-bisphosphate 3-kinase catalytic subunit beta

* HES1: 130 DEGs; HES2: 57 DEGs above the second line; HES3: 2 DEGs above the first line. *Myd88* and *Cxcl1* are the highest top-ranked.

Table_S3 GSE22873_A (c. KO *Ager vs*. WT)_RMA_Ager_HES1_DE_genes

	ENTREZID	SYMBOL	GENENAME
1	14963	***H2-Bl***	histocompatibility 2, blastocyst
2	15042	***H2-T24***	histocompatibility 2, T region locus 24
3	14991	*H2-M3*	histocompatibility 2, M region locus 3
4	110557	*H2-Q6*	histocompatibility 2, Q region locus 6
5	12575	*Cdkn1a*	cyclin-dependent kinase inhibitor 1A (P21)
6	21356	*Tapbp*	TAP binding protein
7	11765	*Ap1g1*	adaptor protein complex AP-1, gamma 1 subunit
8	11764	*Ap1b1*	adaptor protein complex AP-1, beta 1 subunit
9	11767	*Ap1m1*	adaptor-related protein complex AP-1, mu subunit 1
10	14985	*H2-M10.1*	histocompatibility 2, M region locus 10.1
11	15007	*H2-Q10*	histocompatibility 2, Q region locus 10
12	14827	*Pdia3*	protein disulfide isomerase associated 3
13	11766	*Ap1g2*	adaptor protein complex AP-1, gamma 2 subunit
14	12525	*Cd8a*	CD8 antigen, alpha chain
15	16643	*Klrd1*	killer cell lectin-like receptor, subfamily D, member 1
16	14972	*H2-K1*	histocompatibility 2, K1, K region
17	15043	*H2-T3*	histocompatibility 2, T region locus 3
18	12317	*Calr*	calreticulin
19	16641	*Klrc1*	killer cell lectin-like receptor subfamily C, member 1
20	108012	*Ap1s2*	adaptor-related protein complex 1, sigma 2 subunit
21	14997	*H2-M9*	histocompatibility 2, M region locus 9
22	11769	*Ap1s1*	adaptor protein complex AP-1, sigma 1
23	11768	*Ap1m2*	adaptor protein complex AP-1, mu 2 subunit
24	12526	*Cd8b1*	CD8 antigen, beta chain 1
25	14964	*H2-D1*	histocompatibility 2, D region locus 1
26	15013	*H2-Q2*	histocompatibility 2, Q region locus 2
27	12010	*B2m*	beta-2 microglobulin
28	11911	*Atf4*	activating transcription factor 4
29	17691	*Sik1*	salt inducible kinase 1
30	17874	*Myd88*	myeloid differentiation primary response gene 88
31	14825	*Cxcl1*	chemokine (C-X-C motif) ligand 1
32	12765	*Cxcr2*	chemokine (C-X-C motif) receptor 2
33	19697	*Rela*	v-rel reticuloendotheliosis viral oncogene homolog A (avian)
34	12737	*Cldn1*	claudin 1
35	18033	*Nfkb1*	nuclear factor of kappa light polypeptide gene enhancer in B cells 1, p105
36	12770	*Ccr1l1*	chemokine (C-C motif) receptor 1-like 1
37	18021	*Nfatc3*	nuclear factor of activated T cells, cytoplasmic, calcineurin dependent 3
38	12771	*Ccr3*	chemokine (C-C motif) receptor 3
39	12768	*Ccr1*	chemokine (C-C motif) receptor 1
40	12767	*Cxcr4*	chemokine (C-X-C motif) receptor 4
41	15945	*Cxcl10*	chemokine (C-X-C motif) ligand 10
42	23832	*Xcr1*	chemokine (C motif) receptor 1
43	80901	*Cxcr6*	chemokine (C-X-C motif) receptor 6
44	12777	*Ccr10*	chemokine (C-C motif) receptor 10
45	12766	*Cxcr3*	chemokine (C-X-C motif) receptor 3
46	18018	*Nfatc1*	nuclear factor of activated T cells, cytoplasmic, calcineurin dependent 1
47	14281	*Fos*	FBJ osteosarcoma oncogene
48	12774	*Ccr5*	chemokine (C-C motif) receptor 5
49	15251	*Hif1a*	hypoxia inducible factor 1, alpha subunit
50	12772	*Ccr2*	chemokine (C-C motif) receptor 2
51	13051	*Cx3cr1*	chemokine (C-X3-C motif) receptor 1
52	73181	*Nfatc4*	nuclear factor of activated T cells, cytoplasmic, calcineurin dependent 4
53	12773	*Ccr4*	chemokine (C-C motif) receptor 4
54	12458	*Ccr6*	chemokine (C-C motif) receptor 6
55	12769	*Ccr9*	chemokine (C-C motif) receptor 9
56	12775	*Ccr7*	chemokine (C-C motif) receptor 7
57	12145	*Cxcr5*	chemokine (C-X-C motif) receptor 5
58	12776	*Ccr8*	chemokine (C-C motif) receptor 8
59	18019	*Nfatc2*	nuclear factor of activated T cells, cytoplasmic, calcineurin dependent 2
60	16476	*Jun*	jun proto-oncogene
61	11537	*Cfd*	complement factor D (adipsin)

62	12266	*C3*	complement component 3
63	12914	*Crebbp*	CREB binding protein
64	16391	*Irf9*	interferon regulatory factor 9
65	328572	*Ep300*	E1A binding protein p300
66	27375	*Tjp3*	tight junction protein 3
67	208647	*Creb3l2*	cAMP responsive element binding protein 3-like 2
68	54123	*Irf7*	interferon regulatory factor 7
69	11909	*Atf2*	activating transcription factor 2
70	14609	*Gja1*	gap junction protein, alpha 1
71	22154	*Tubb5*	tubulin, beta 5 class I
72	54131	*Irf3*	interferon regulatory factor 3
73	20846	*Stat1*	signal transducer and activator of transcription 1
74	20847	*Stat2*	signal transducer and activator of transcription 2
75	208677	*Creb3l3*	cAMP responsive element binding protein 3-like 3
76	12912	*Creb1*	cAMP responsive element binding protein 1
77	74343	*Crtc2*	CREB regulated transcription coactivator 2
78	78284	*Creb3l4*	cAMP responsive element binding protein 3-like 4
79	26427	*Creb3l1*	cAMP responsive element binding protein 3-like 1
80	12913	*Creb3*	cAMP responsive element binding protein 3
81	231991	*Creb5*	cAMP responsive element binding protein 5
82	12739	*Cldn3*	claudin 3
83	20852	*Stat6*	signal transducer and activator of transcription 6
84	18712	*Pim1*	proviral integration site 1
85	19699	*Reln*	reelin
86	16419	*Itgb5*	integrin beta 5
87	14962	*Cfb*	complement factor B
88	109700	*Itga1*	integrin alpha 1
89	19303	*Pxn*	paxillin
90	54635	*Pdgfc*	platelet-derived growth factor, C polypeptide
91	13867	*Erbb3*	erb-b2 receptor tyrosine kinase 3
92	22145	*Tuba4a*	tubulin, alpha 4A
93	12741	*Cldn5*	claudin 5
94	18260	*Ocln*	occludin
95	16367	*Irs1*	insulin receptor substrate 1
96	53857	*Tuba8*	tubulin, alpha 8
97	12389	*Cav1*	caveolin 1, caveolae protein
98	20351	*Sema4a*	sema domain, immunoglobulin domain (Ig), transmembrane domain (TM) and short cytoplasmic domain, (semaphorin) 4A
99	140570	*Plxnb2*	plexin B2
100	18048	*Klk1b4*	kallikrein 1-related pepidase b4
101	14463	*Gata4*	GATA binding protein 4
102	13424	*Dync1h1*	dynein cytoplasmic 1 heavy chain 1
103	94230	*Cpsf1*	cleavage and polyadenylation specific factor 1
104	19046	*Ppp1cb*	protein phosphatase 1 catalytic subunit beta
105	54140	*Avpr1a*	arginine vasopressin receptor 1A
106	18796	*Plcb2*	phospholipase C, beta 2
107	93742	*Pard3*	par-3 family cell polarity regulator
108	19349	*Rab7*	RAB7, member RAS oncogene family
109	17281	*Fyco1*	FYVE and coiled-coil domain containing 1
110	12649	*Chek1*	checkpoint kinase 1
111	22390	*Wee1*	WEE 1 homolog 1 (S. pombe)
112	104099	*Itga9*	integrin alpha 9
113	16542	*Kdr*	kinase insert domain protein receptor
114	20850	*Stat5a*	signal transducer and activator of transcription 5A
115	19268	*Ptprf*	protein tyrosine phosphatase, receptor type, F
116	226421	*Rab7b*	RAB7B, member RAS oncogene family
117	12738	*Cldn2*	claudin 2
118	20851	*Stat5b*	signal transducer and activator of transcription 5B
119	215303	*Camk1g*	calcium/calmodulin-dependent protein kinase I gamma
120	11607	*Agtr1a*	angiotensin II receptor, type 1a
121	16403	*Itga6*	integrin alpha 6
122	16410	*Itgav*	integrin alpha V
123	74987	*Cldn34d*	claudin 34D
124	14199	*Fhl1*	four and a half LIM domains 1

125	17342	*Mitf*	melanogenesis associated transcription factor
126	13190	*Dct*	dopachrome tautomerase
127	20310	*Cxcl2*	chemokine (C-X-C motif) ligand 2
128	14700	*Gng10*	guanine nucleotide binding protein (G protein), gamma 10
129	20181	*Rxra*	retinoid X receptor alpha
130	18830	*Pltp*	phospholipid transfer protein
131	22330	*Vcl*	vinculin
132	22393	*Wfs1*	wolframin ER transmembrane glycoprotein
133	12915	*Atf6b*	activating transcription factor 6 beta
134	21872	*Tjp1*	tight junction protein 1
135	53624	*Cldn7*	claudin 7

* HES1: 135 DEGs; HES2: 27 DEGs above the second line; HES3: 2 DEGs above the first line. *H2-Bl* and *H2-T24* are the highest top-ranked.

Table_S4 GSE22873_MAvM (KO *Myd88_Ager vs.* KO *Myd88*)_RMA_Ager_HES1_DE_genes

	ENTREZID	SYMBOL	GENENAME
1	14963	H2-Bl	histocompatibility 2, blastocyst
2	15042	H2-T24	histocompatibility 2, T region locus 24
3	110557	H2-Q6	histocompatibility 2, Q region locus 6
4	14985	H2-M10.1	histocompatibility 2, M region locus 10.1
5	12317	Calr	calreticulin
6	108012	Ap1s2	adaptor-related protein complex 1, sigma 2 subunit
7	11767	Ap1m1	adaptor-related protein complex AP-1, mu subunit 1
8	11765	Ap1g1	adaptor protein complex AP-1, gamma 1 subunit
9	11766	Ap1g2	adaptor protein complex AP-1, gamma 2 subunit
10	14997	H2-M9	histocompatibility 2, M region locus 9
11	12526	Cd8b1	CD8 antigen, beta chain 1
12	12575	Cdkn1a	cyclin-dependent kinase inhibitor 1A (P21)
13	11764	Ap1b1	adaptor protein complex AP-1, beta 1 subunit
14	21356	Tapbp	TAP binding protein
15	15043	H2-T3	histocompatibility 2, T region locus 3
16	14964	H2-D1	histocompatibility 2, D region locus 1
17	15007	H2-Q10	histocompatibility 2, Q region locus 10
18	16643	Klrd1	killer cell lectin-like receptor, subfamily D, member 1
19	11769	Ap1s1	adaptor protein complex AP-1, sigma 1
20	14827	Pdia3	protein disulfide isomerase associated 3
21	16641	Klrc1	killer cell lectin-like receptor subfamily C, member 1
22	11768	Ap1m2	adaptor protein complex AP-1, mu 2 subunit
23	14972	H2-K1	histocompatibility 2, K1, K region
24	15013	H2-Q2	histocompatibility 2, Q region locus 2
25	12525	Cd8a	CD8 antigen, alpha chain
26	14991	H2-M3	histocompatibility 2, M region locus 3
27	12010	B2m	beta-2 microglobulin
28	384783	Irs2	insulin receptor substrate 2
29	18708	Pik3r1	phosphoinositide-3-kinase regulatory subunit 1
30	208647	Creb3l2	cAMP responsive element binding protein 3-like 2
31	17691	Sik1	salt inducible kinase 1
32	14282	Fosb	FBJ osteosarcoma oncogene B
33	19013	Ppara	peroxisome proliferator activated receptor alpha
34	17869	Myc	myelocytomatosis oncogene
35	20787	Srebf1	sterol regulatory element binding transcription factor 1
36	12702	Socs3	suppressor of cytokine signaling 3
37	12914	Crebbp	CREB binding protein
38	27056	Irf5	interferon regulatory factor 5
39	20302	Ccl3	chemokine (C-C motif) ligand 3
40	16476	Jun	jun proto-oncogene
41	14784	Grb2	growth factor receptor bound protein 2
42	74769	Pik3cb	phosphatidylinositol-4,5-bisphosphate 3-kinase catalytic subunit beta
43	12737	Cldn1	claudin 1
44	16193	Il6	interleukin 6
45	14281	Fos	FBJ osteosarcoma oncogene
46	18706	Pik3ca	phosphatidylinositol-4,5-bisphosphate 3-kinase catalytic subunit alpha
47	18830	Pltp	phospholipid transfer protein
48	18754	Prkce	protein kinase C, epsilon
49	228775	Trib3	tribbles pseudokinase 3
50	58988	Rps6kb2	ribosomal protein S6 kinase, polypeptide 2
51	12913	Creb3	cAMP responsive element binding protein 3
52	12915	Atf6b	activating transcription factor 6 beta
53	17436	Me1	malic enzyme 1, NADP(+)-dependent, cytosolic
54	328572	Ep300	E1A binding protein p300
55	18710	Pik3r3	phosphoinositide-3-kinase regulatory subunit 3

* HES1: 55 DEGs; *H2-Bl* and *H2-T24* are the highest top-ranked.

Table_S5 GSE22873_MAvA (KO *Myd88_Ager vs.* KO *Ager*)_RMA_Ager_HES1_DE_genes

	ENTREZID	SYMBOL	GENENAME
1	17874	Myd88	myeloid differentiation primary response gene 88
2	14825	Cxcl1	chemokine (C-X-C motif) ligand 1
3	12767	Cxcr4	chemokine (C-X-C motif) receptor 4
4	18033	Nfkb1	nuclear factor of kappa light polypeptide gene enhancer in B cells 1, p105
5	14677	Gnai1	guanine nucleotide binding protein (G protein), alpha inhibiting 1
6	18708	Pik3r1	phosphoinositide-3-kinase regulatory subunit 1
7	12777	Ccr10	chemokine (C-C motif) receptor 10
8	12776	Ccr8	chemokine (C-C motif) receptor 8
9	12145	Cxcr5	chemokine (C-X-C motif) receptor 5
10	16476	Jun	jun proto-oncogene
11	73181	Nfatc4	nuclear factor of activated T cells, cytoplasmic, calcineurin dependent 4
12	74747	Ddit4	DNA-damage-inducible transcript 4
13	22084	Tsc2	TSC complex subunit 2
14	66066	Gng11	guanine nucleotide binding protein (G protein), gamma 11
15	18019	Nfatc2	nuclear factor of activated T cells, cytoplasmic, calcineurin dependent 2
16	12766	Cxcr3	chemokine (C-X-C motif) receptor 3
17	14281	Fos	FBJ osteosarcoma oncogene
18	12768	Ccr1	chemokine (C-C motif) receptor 1
19	18018	Nfatc1	nuclear factor of activated T cells, cytoplasmic, calcineurin dependent 1
20	23832	Xcr1	chemokine (C motif) receptor 1
21	12770	Ccr1l1	chemokine (C-C motif) receptor 1-like 1
22	14700	Gng10	guanine nucleotide binding protein (G protein), gamma 10
23	12774	Ccr5	chemokine (C-C motif) receptor 5
24	12771	Ccr3	chemokine (C-C motif) receptor 3
25	12772	Ccr2	chemokine (C-C motif) receptor 2
26	18021	Nfatc3	nuclear factor of activated T cells, cytoplasmic, calcineurin dependent 3
27	12765	Cxcr2	chemokine (C-X-C motif) receptor 2
28	12773	Ccr4	chemokine (C-C motif) receptor 4
29	12769	Ccr9	chemokine (C-C motif) receptor 9
30	16193	Il6	interleukin 6
31	12458	Ccr6	chemokine (C-C motif) receptor 6
32	15251	Hif1a	hypoxia inducible factor 1, alpha subunit
33	13051	Cx3cr1	chemokine (C-X3-C motif) receptor 1
34	12775	Ccr7	chemokine (C-C motif) receptor 7
35	19697	Rela	v-rel reticuloendotheliosis viral oncogene homolog A (avian)
36	80901	Cxcr6	chemokine (C-X-C motif) receptor 6
37	67046	Tbc1d7	TBC1 domain family, member 7
38	20310	Cxcl2	chemokine (C-X-C motif) ligand 2
39	13636	Efna1	ephrin A1
40	14182	Fgfr1	fibroblast growth factor receptor 1
41	13649	Egfr	epidermal growth factor receptor
42	16177	Il1r1	interleukin 1 receptor, type I
43	29857	Mapk12	mitogen-activated protein kinase 12
44	384783	Irs2	insulin receptor substrate 2
45	64930	Tsc1	TSC complex subunit 1
46	26416	Mapk14	mitogen-activated protein kinase 14
47	13867	Erbb3	erb-b2 receptor tyrosine kinase 3
48	15461	Hras	Harvey rat sarcoma virus oncogene
49	13610	S1pr3	sphingosine-1-phosphate receptor 3
50	17295	Met	met proto-oncogene
51	19228	Pth1r	parathyroid hormone 1 receptor
52	84112	Sucnr1	succinate receptor 1
53	14688	Gnb1	guanine nucleotide binding protein (G protein), beta 1
54	16542	Kdr	kinase insert domain protein receptor
55	16337	Insr	insulin receptor
56	16179	Irak1	interleukin-1 receptor-associated kinase 1
57	14360	Fyn	Fyn proto-oncogene
58	108068	Grm2	glutamate receptor, metabotropic 2

59	19016	Pparg	peroxisome proliferator activated receptor gamma
60	12491	Cd36	CD36 molecule
61	18176	Nras	neuroblastoma ras oncogene
62	223864	Rapgef3	Rap guanine nucleotide exchange factor (GEF) 3
63	17773	Mtnr1a	melatonin receptor 1A
64	14254	Flt1	FMS-like tyrosine kinase 1
65	22637	Zap70	zeta-chain (TCR) associated protein kinase
66	18783	Pla2g4a	phospholipase A2, group IVA (cytosolic, calcium-dependent)
67	18795	Plcb1	phospholipase C, beta 1

* HES1: 67 DEGs; *Myd88* and *Cxcl1* are the highest top-ranked.

Table_S6 GSE22873_MvA (KO *Myd88 vs.* KO *Ager*)_RMA_Ager_HES1_DE_genes

	ENTREZID	SYMBOL	GENENAME
1	14963	H2-Bl	histocompatibility 2, blastocyst
2	14985	H2-M10.1	histocompatibility 2, M region locus 10.1
3	17874	Myd88	myeloid differentiation primary response gene 88
4	14825	Cxcl1	chemokine (C-X-C motif) ligand 1
5	74747	Ddit4	DNA-damage-inducible transcript 4
6	22084	Tsc2	TSC complex subunit 2
7	15251	Hif1a	hypoxia inducible factor 1, alpha subunit
8	12575	Cdkn1a	cyclin-dependent kinase inhibitor 1A (P21)
9	12526	Cd8b1	CD8 antigen, beta chain 1
10	14827	Pdia3	protein disulfide isomerase associated 3
11	84112	Sucnr1	succinate receptor 1
12	14677	Gnai1	guanine nucleotide binding protein (G protein), alpha inhibiting 1
13	64930	Tsc1	TSC complex subunit 1
14	67046	Tbc1d7	TBC1 domain family, member 7
15	15042	H2-T24	histocompatibility 2, T region locus 24
16	11768	Ap1m2	adaptor protein complex AP-1, mu 2 subunit
17	15043	H2-T3	histocompatibility 2, T region locus 3
18	14991	H2-M3	histocompatibility 2, M region locus 3
19	11765	Ap1g1	adaptor protein complex AP-1, gamma 1 subunit
20	11764	Ap1b1	adaptor protein complex AP-1, beta 1 subunit
21	108012	Ap1s2	adaptor-related protein complex 1, sigma 2 subunit
22	16641	Klrc1	killer cell lectin-like receptor subfamily C, member 1
23	16643	Klrd1	killer cell lectin-like receptor, subfamily D, member 1
24	14964	H2-D1	histocompatibility 2, D region locus 1
25	19016	Pparg	peroxisome proliferator activated receptor gamma
26	12491	Cd36	CD36 molecule
27	12525	Cd8a	CD8 antigen, alpha chain
28	21356	Tapbp	TAP binding protein
29	11769	Ap1s1	adaptor protein complex AP-1, sigma 1
30	12010	B2m	beta-2 microglobulin
31	12317	Calr	calreticulin
32	15007	H2-Q10	histocompatibility 2, Q region locus 10
33	14997	H2-M9	histocompatibility 2, M region locus 9
34	14972	H2-K1	histocompatibility 2, K1, K region
35	110557	H2-Q6	histocompatibility 2, Q region locus 6
36	15013	H2-Q2	histocompatibility 2, Q region locus 2
37	11767	Ap1m1	adaptor-related protein complex AP-1, mu subunit 1
38	11766	Ap1g2	adaptor protein complex AP-1, gamma 2 subunit
39	11555	Adrb2	adrenergic receptor, beta 2
40	11512	Adcy6	adenylate cyclase 6
41	66513	Tab1	TGF-beta activated kinase 1/MAP3K7 binding protein 1
42	80885	Hcar2	hydroxycarboxylic acid receptor 2
43	16176	Il1b	interleukin 1 beta
44	14688	Gnb1	guanine nucleotide binding protein (G protein), beta 1
45	15162	Hck	hemopoietic cell kinase
46	21825	Thbs1	thrombospondin 1
47	16179	Irak1	interleukin-1 receptor-associated kinase 1
48	13610	S1pr3	sphingosine-1-phosphate receptor 3
49	17773	Mtnr1a	melatonin receptor 1A
50	74769	Pik3cb	phosphatidylinositol-4,5-bisphosphate 3-kinase catalytic subunit beta
51	14823	Grm8	glutamate receptor, metabotropic 8
52	70839	P2ry12	purinergic receptor P2Y, G-protein coupled 12
53	16159	Il12a	interleukin 12a
54	16193	Il6	interleukin 6
55	108068	Grm2	glutamate receptor, metabotropic 2
56	18754	Prkce	protein kinase C, epsilon
57	170743	Tlr7	toll-like receptor 7
58	66066	Gng11	guanine nucleotide binding protein (G protein), gamma 11
59	18747	Prkaca	protein kinase, cAMP dependent, catalytic, alpha

60	24088	Tlr2	toll-like receptor 2
61	11539	Adora1	adenosine A1 receptor
62	26409	Map3k7	mitogen-activated protein kinase kinase kinase 7
63	14191	Fgr	FGR proto-oncogene, Src family tyrosine kinase
64	20847	Stat2	signal transducer and activator of transcription 2
65	12767	Cxcr4	chemokine (C-X-C motif) receptor 4
66	14700	Gng10	guanine nucleotide binding protein (G protein), gamma 10
67	12669	Chrm1	cholinergic receptor, muscarinic 1, CNS
68	64337	Gng13	guanine nucleotide binding protein (G protein), gamma 13
69	11835	Ar	androgen receptor
70	18126	Nos2	nitric oxide synthase 2, inducible
71	56508	Rapgef4	Rap guanine nucleotide exchange factor (GEF) 4
72	12773	Ccr4	chemokine (C-C motif) receptor 4
73	15461	Hras	Harvey rat sarcoma virus oncogene
74	18390	Oprm1	opioid receptor, mu 1
75	17087	Ly96	lymphocyte antigen 96
76	15974	Ifnab	interferon alpha B
77	18752	Prkcg	protein kinase C, gamma
78	13115	Cyp27b1	cytochrome P450, family 27, subfamily b, polypeptide 1
79	11556	Adrb3	adrenergic receptor, beta 3
80	12845	Comp	cartilage oligomeric matrix protein
81	22337	Vdr	vitamin D (1,25-dihydroxyvitamin D3) receptor
82	14282	Fosb	FBJ osteosarcoma oncogene B
83	14281	Fos	FBJ osteosarcoma oncogene
84	21926	Tnf	tumor necrosis factor
85	12374	Casr	calcium-sensing receptor
86	18176	Nras	neuroblastoma ras oncogene
87	20609	Sstr5	somatostatin receptor 5
88	54652	Cacna1f	calcium channel, voltage-dependent, alpha 1F subunit
89	21827	Thbs3	thrombospondin 3
90	15968	Ifna5	interferon alpha 5
91	94226	S1pr5	sphingosine-1-phosphate receptor 5
92	432530	Adcy1	adenylate cyclase 1
93	13611	S1pr4	sphingosine-1-phosphate receptor 4
94	243270	Hcar1	hydrocarboxylic acid receptor 1
95	18706	Pik3ca	phosphatidylinositol-4,5-bisphosphate 3-kinase catalytic subunit alpha
96	170744	Tlr8	toll-like receptor 8
97	11554	Adrb1	adrenergic receptor, beta 1
98	19013	Ppara	peroxisome proliferator activated receptor alpha
99	666168	Cyp4a31	cytochrome P450, family 4, subfamily a, polypeptide 31
100	210044	Adcy2	adenylate cyclase 2
101	12776	Ccr8	chemokine (C-C motif) receptor 8
102	20846	Stat1	signal transducer and activator of transcription 1
103	66724	Tab3	TGF-beta activated kinase 1/MAP3K7 binding protein 3
104	223864	Rapgef3	Rap guanine nucleotide exchange factor (GEF) 3
105	15977	Ifnb1	interferon beta 1, fibroblast
106	15967	Ifna4	interferon alpha 4
107	105787	Prkaa1	protein kinase, AMP-activated, alpha 1 catalytic subunit
108	14699	Gngt1	guanine nucleotide binding protein (G protein), gamma transducing activity polypeptide 1
109	13489	Drd2	dopamine receptor D2
110	16180	Il1rap	interleukin 1 receptor accessory protein
111	81897	Tlr9	toll-like receptor 9
112	14702	Gng2	guanine nucleotide binding protein (G protein), gamma 2
113	14706	Gng4	guanine nucleotide binding protein (G protein), gamma 4
114	14745	Lpar1	lysophosphatidic acid receptor 1
115	13491	Drd4	dopamine receptor D4
116	18430	Oxtr	oxytocin receptor
117	12774	Ccr5	chemokine (C-C motif) receptor 5
118	26415	Mapk13	mitogen-activated protein kinase 13
119	14082	Fadd	Fas (TNFRSF6)-associated via death domain

120	21899	Tlr6	toll-like receptor 6
121	17391	Mmp24	matrix metallopeptidase 24
122	18707	Pik3cd	phosphatidylinositol-4,5-bisphosphate 3-kinase catalytic subunit delta
123	16522	Kcnj6	potassium inwardly-rectifying channel, subfamily J, member 6
124	18749	Prkacb	protein kinase, cAMP dependent, catalytic, beta
125	19217	Ptger2	prostaglandin E receptor 2 (subtype EP2)
126	26416	Mapk14	mitogen-activated protein kinase 14
127	12292	Cacna1s	calcium channel, voltage-dependent, L type, alpha 1S subunit
128	19219	Ptger4	prostaglandin E receptor 4 (subtype EP4)
129	104110	Adcy4	adenylate cyclase 4
130	329502	Pla2g4e	phospholipase A2, group IVE
131	381489	Rxfp1	relaxin/insulin-like family peptide receptor 1
132	18750	Prkca	protein kinase C, alpha
133	14704	Gng3	guanine nucleotide binding protein (G protein), gamma 3
134	19228	Pth1r	parathyroid hormone 1 receptor
135	21898	Tlr4	toll-like receptor 4
136	12777	Ccr10	chemokine (C-C motif) receptor 10
137	30955	Pik3cg	phosphatidylinositol-4,5-bisphosphate 3-kinase catalytic subunit gamma
138	19739	Rgs9	regulator of G-protein signaling 9
139	17096	Lyn	LYN proto-oncogene, Src family tyrosine kinase
140	12672	Chrm4	cholinergic receptor, muscarinic 4
141	18798	Plcb4	phospholipase C, beta 4
142	12770	Ccr1l1	chemokine (C-C motif) receptor 1-like 1
143	266632	Irak4	interleukin-1 receptor-associated kinase 4
144	18127	Nos3	nitric oxide synthase 3, endothelial cell
145	12766	Cxcr3	chemokine (C-X-C motif) receptor 3
146	15552	Htr1d	5-hydroxytryptamine (serotonin) receptor 1D
147	12921	Crhr1	corticotropin releasing hormone receptor 1
148	19216	Ptger1	prostaglandin E receptor 1 (subtype EP1)
149	12458	Ccr6	chemokine (C-C motif) receptor 6
150	16160	Il12b	interleukin 12b
151	104111	Adcy3	adenylate cyclase 3
152	16177	Il1r1	interleukin 1 receptor, type I
153	21897	Tlr1	toll-like receptor 1
154	15550	Htr1a	5-hydroxytryptamine (serotonin) receptor 1A
155	18796	Plcb2	phospholipase C, beta 2
156	15551	Htr1b	5-hydroxytryptamine (serotonin) receptor 1B
157	16521	Kcnj5	potassium inwardly-rectifying channel, subfamily J, member 5
158	22034	Traf6	TNF receptor-associated factor 6
159	23832	Xcr1	chemokine (C motif) receptor 1
160	15972	Ifna9	interferon alpha 9
161	12768	Ccr1	chemokine (C-C motif) receptor 1
162	68652	Tab2	TGF-beta activated kinase 1/MAP3K7 binding protein 2
163	208188	Ghsr	growth hormone secretagogue receptor
164	20310	Cxcl2	chemokine (C-X-C motif) ligand 2
165	18783	Pla2g4a	phospholipase A2, group IVA (cytosolic, calcium-dependent)
166	14709	Gng8	guanine nucleotide binding protein (G protein), gamma 8
167	330122	Cxcl3	chemokine (C-X-C motif) ligand 3
168	29857	Mapk12	mitogen-activated protein kinase 12
169	14739	S1pr2	sphingosine-1-phosphate receptor 2
170	67839	Gpsm1	G-protein signalling modulator 1 (AGS3-like, C. elegans)
171	20779	Src	Rous sarcoma oncogene
172	14693	Gnb2	guanine nucleotide binding protein (G protein), beta 2
173	104709	Pik3r6	phosphoinositide-3-kinase regulatory subunit 5
174	224129	Adcy5	adenylate cyclase 5
175	20607	Sstr3	somatostatin receptor 3
176	108079	Prkaa2	protein kinase, AMP-activated, alpha 2 catalytic subunit
177	20182	Rxrb	retinoid X receptor beta
178	18709	Pik3r2	phosphoinositide-3-kinase regulatory subunit 2
179	14816	Grm1	glutamate receptor, metabotropic 1
180	17387	Mmp14	matrix metallopeptidase 14 (membrane-inserted)

181	20606	Sstr2	somatostatin receptor 2
182	271639	Adcy10	adenylate cyclase 10
183	18708	Pik3r1	phosphoinositide-3-kinase regulatory subunit 1
184	12286	Cacna1a	calcium channel, voltage-dependent, P/Q type, alpha 1A subunit
185	268934	Grm4	glutamate receptor, metabotropic 4
186	97064	Wwtr1	WW domain containing transcription regulator 1
187	14219	Ccn2	cellular communication network factor 2
188	12289	Cacna1d	calcium channel, voltage-dependent, L type, alpha 1D subunit
189	13618	Ednrb	endothelin receptor type B
190	12769	Ccr9	chemokine (C-C motif) receptor 9
191	14696	Gnb4	guanine nucleotide binding protein (G protein), beta 4
192	19218	Ptger3	prostaglandin E receptor 3 (subtype EP3)
193	108073	Grm7	glutamate receptor, metabotropic 7
194	80901	Cxcr6	chemokine (C-X-C motif) receptor 6
195	12771	Ccr3	chemokine (C-C motif) receptor 3
196	18795	Plcb1	phospholipase C, beta 1
197	18797	Plcb3	phospholipase C, beta 3
198	241113	Prkag3	protein kinase, AMP-activated, gamma 3 non-catalytic subunit
199	13617	Ednra	endothelin receptor type A
200	14710	Gngt2	guanine nucleotide binding protein (G protein), gamma transducing activity polypeptide 2
201	271844	Pla2g4f	phospholipase A2, group IVF
202	12801	Cnr1	cannabinoid receptor 1 (brain)
203	18830	Pltp	phospholipid transfer protein
204	18166	Npy1r	neuropeptide Y receptor Y1
205	13051	Cx3cr1	chemokine (C-X3-C motif) receptor 1
206	18386	Oprd1	opioid receptor, delta 1
207	14695	Gnb3	guanine nucleotide binding protein (G protein), beta 3
208	12772	Ccr2	chemokine (C-C motif) receptor 2
209	14697	Gnb5	guanine nucleotide binding protein (G protein), beta 5
210	23796	Aplnr	apelin receptor
211	108069	Grm3	glutamate receptor, metabotropic 3
212	11513	Adcy7	adenylate cyclase 7
213	19416	Rasd1	RAS, dexamethasone-induced 1
214	22340	Vegfb	vascular endothelial growth factor B
215	13649	Egfr	epidermal growth factor receptor
216	50780	Rgs3	regulator of G-protein signaling 3
217	13490	Drd3	dopamine receptor D3
218	12287	Cacna1b	calcium channel, voltage-dependent, N type, alpha 1B subunit
219	19094	Mapk11	mitogen-activated protein kinase 11
220	11515	Adcy9	adenylate cyclase 9
221	16524	Kcnj9	potassium inwardly-rectifying channel, subfamily J, member 9
222	14708	Gng7	guanine nucleotide binding protein (G protein), gamma 7
223	12062	Bdkrb2	bradykinin receptor, beta 2
224	14701	Gng12	guanine nucleotide binding protein (G protein), gamma 12
225	17388	Mmp15	matrix metallopeptidase 15
226	18710	Pik3r3	phosphoinositide-3-kinase regulatory subunit 3
227	320207	Pik3r5	phosphoinositide-3-kinase regulatory subunit 5
228	108099	Prkag2	protein kinase, AMP-activated, gamma 2 non-catalytic subunit
229	12765	Cxcr2	chemokine (C-X-C motif) receptor 2
230	12288	Cacna1c	calcium channel, voltage-dependent, L type, alpha 1C subunit
231	78390	Pla2g4d	phospholipase A2, group IVD
232	17389	Mmp16	matrix metallopeptidase 16
233	23948	Mmp17	matrix metallopeptidase 17
234	15564	Htr5b	5-hydroxytryptamine (serotonin) receptor 5B
235	12775	Ccr7	chemokine (C-C motif) receptor 7
236	26414	Mapk10	mitogen-activated protein kinase 10
237	11514	Adcy8	adenylate cyclase 8
238	16519	Kcnj3	potassium inwardly-rectifying channel, subfamily J, member 3
239	18751	Prkcb	protein kinase C, beta
240	20605	Sstr1	somatostatin receptor 1

241	15563	Htr5a	5-hydroxytryptamine (serotonin) receptor 5A
242	20265	Scn1a	sodium channel, voltage-gated, type I, alpha
243	16001	Igf1r	insulin-like growth factor I receptor
244	54393	Gabbr1	gamma-aminobutyric acid (GABA) B receptor, 1
245	26419	Mapk8	mitogen-activated protein kinase 8
246	16653	Kras	Kirsten rat sarcoma viral oncogene homolog
247	232889	Pla2g4c	phospholipase A2, group IVC (cytosolic, calcium-independent)
248	233079	Ffar2	free fatty acid receptor 2
249	15557	Htr1f	5-hydroxytryptamine (serotonin) receptor 1F
250	13609	S1pr1	sphingosine-1-phosphate receptor 1
251	26413	Mapk1	mitogen-activated protein kinase 1
252	12145	Cxcr5	chemokine (C-X-C motif) receptor 5
253	26420	Mapk9	mitogen-activated protein kinase 9
254	17436	Me1	malic enzyme 1, NADP(+)-dependent, cytosolic
255	20183	Rxrg	retinoid X receptor gamma
256	15182	Hdac2	histone deacetylase 2
257	93759	Sirt1	sirtuin 1
258	21828	Thbs4	thrombospondin 4
259	19082	Prkag1	protein kinase, AMP-activated, gamma 1 non-catalytic subunit
260	21826	Thbs2	thrombospondin 2
261	19079	Prkab1	protein kinase, AMP-activated, beta 1 non-catalytic subunit
262	18227	Nr4a2	nuclear receptor subfamily 4, group A, member 2
263	20181	Rxra	retinoid X receptor alpha
264	18033	Nfkb1	nuclear factor of kappa light polypeptide gene enhancer in B cells 1, p105
265	108097	Prkab2	protein kinase, AMP-activated, beta 2 non-catalytic subunit
266	14969	H2-Eb1	histocompatibility 2, class II antigen E beta
267	14961	H2-Ab1	histocompatibility 2, class II antigen A, beta 1
268	64654	Fgf23	fibroblast growth factor 23
269	20663	Sos2	SOS Ras/Rho guanine nucleotide exchange factor 2
270	72780	Rspo3	R-spondin 3
271	14160	Lgr5	leucine rich repeat containing G protein coupled receptor 5
272	113868	Acaa1a	acetyl-Coenzyme A acyltransferase 1A
273	12737	Cldn1	claudin 1
274	55985	Cxcl13	chemokine (C-X-C motif) ligand 13
275	26427	Creb3l1	cAMP responsive element binding protein 3-like 1
276	23957	Nr0b2	nuclear receptor subfamily 0, group B, member 2
277	28250	Slco1a4	solute carrier organic anion transporter family, member 1a4
278	18048	Klk1b4	kallikrein 1-related pepidase b4
279	20111	Rps6ka1	ribosomal protein S6 kinase polypeptide 1
280	53412	Ppp1r3c	protein phosphatase 1, regulatory subunit 3C
281	14784	Grb2	growth factor receptor bound protein 2
282	54123	Irf7	interferon regulatory factor 7
283	22027	Hsp90b1	heat shock protein 90, beta (Grp94), member 1
284	13854	Epn1	epsin 1
285	13038	Ctsk	cathepsin K
286	20411	Sorbs1	sorbin and SH3 domain containing 1
287	14733	Gpc1	glypican 1
288	14960	H2-Aa	histocompatibility 2, class II antigen A, alpha
289	14182	Fgfr1	fibroblast growth factor receptor 1
290	20845	Star	steroidogenic acute regulatory protein
291	14673	Gna12	guanine nucleotide binding protein, alpha 12
292	54140	Avpr1a	arginine vasopressin receptor 1A
293	16476	Jun	jun proto-oncogene
294	17691	Sik1	salt inducible kinase 1
295	18024	Nfe2l2	nuclear factor, erythroid derived 2, like 2
296	103140	Gstt3	glutathione S-transferase, theta 3
297	12311	Calcr	calcitonin receptor
298	16391	Irf9	interferon regulatory factor 9
299	16416	Itgb3	integrin beta 3
300	93672	Il24	interleukin 24
301	15200	Hbegf	heparin-binding EGF-like growth factor

302	68094	Smarcc2	SWI/SNF related, matrix associated, actin dependent regulator of chromatin, subfamily c, member 2
303	208677	Creb3l3	cAMP responsive element binding protein 3-like 3
304	14672	Gna11	guanine nucleotide binding protein, alpha 11
305	192199	Rspo1	R-spondin 1
306	20848	Stat3	signal transducer and activator of transcription 3
307	18712	Pim1	proviral integration site 1
308	13122	Cyp7a1	cytochrome P450, family 7, subfamily a, polypeptide 1
309	239405	Rspo2	R-spondin 2
310	246221	Mpst	mercaptopyruvate sulfurtransferase
311	69372	Mocs3	molybdenum cofactor synthesis 3
312	12978	Csf1r	colony stimulating factor 1 receptor
313	108664	Atp6v1h	ATPase, H+ transporting, lysosomal V1 subunit H
314	14998	H2-DMa	histocompatibility 2, class II, locus DMa
315	16149	Cd74	CD74 antigen (invariant polypeptide of major histocompatibility complex, class II antigen-associated)
316	12915	Atf6b	activating transcription factor 6 beta
317	78284	Creb3l4	cAMP responsive element binding protein 3-like 4
318	12313	Calm1	calmodulin 1
319	12550	Cdh1	cadherin 1
320	16480	Jup	junction plakoglobin
321	23893	Grem2	gremlin 2, DAN family BMP antagonist
322	12156	Bmp2	bone morphogenetic protein 2
323	22627	Ywhae	tyrosine 3-monooxygenase/tryptophan 5-monooxygenase activation protein, epsilon polypeptide
324	11909	Atf2	activating transcription factor 2
325	73181	Nfatc4	nuclear factor of activated T cells, cytoplasmic, calcineurin dependent 4
326	11911	Atf4	activating transcription factor 4
327	12568	Cdk5	cyclin-dependent kinase 5
328	11433	Acp5	acid phosphatase 5, tartrate resistant
329	16000	Igf1	insulin-like growth factor 1
330	19206	Ptch1	patched 1
331	14451	Gas1	growth arrest specific 1
332	208647	Creb3l2	cAMP responsive element binding protein 3-like 2
333	18104	Nqo1	NAD(P)H dehydrogenase, quinone 1
334	18021	Nfatc3	nuclear factor of activated T cells, cytoplasmic, calcineurin dependent 3
335	19046	Ppp1cb	protein phosphatase 1 catalytic subunit beta
336	235320	Zbtb16	zinc finger and BTB domain containing 16
337	13117	Cyp4a10	cytochrome P450, family 4, subfamily a, polypeptide 10
338	20304	Ccl5	chemokine (C-C motif) ligand 5
339	74343	Crtc2	CREB regulated transcription coactivator 2
340	228026	Pdk1	pyruvate dehydrogenase kinase, isoenzyme 1
341	23872	Ets2	E26 avian leukemia oncogene 2, 3' domain
342	231991	Creb5	cAMP responsive element binding protein 5
343	19697	Rela	v-rel reticuloendotheliosis viral oncogene homolog A (avian)
344	12161	Bmp6	bone morphogenetic protein 6
345	13653	Egr1	early growth response 1
346	72930	Ppp2r2b	protein phosphatase 2, regulatory subunit B, beta
347	14999	H2-DMb1	histocompatibility 2, class II, locus Mb1

* HES1: 347 DEGs; *H2-Bl*, *H2-M10.1*, *Myd88* and *Cxcl1* are the four highest top-ranked.

TABLE S7 True KO signaling pathways identified by SPIA from GSE22873

Order	Pathway ID	Pathway Name	Status	pSize	DEGs (%)	pGFdr
**Pathways identified from GSE22873_MvA (KO *Myd88* *vs.* KO *Ager*)**						
1	5170	Human immunodeficiency virus 1 infection	Inhibited	194	85(43.81)	7.65E-47
**2**	**4620**	**Toll-like receptor signaling pathway**	**Inhibited**	**87**	**48(55.17)**	**1.88E-31**
3	5161	Hepatitis B	Inhibited	149	59(39.59)	5.20E-29
4	5142	Chagas disease (American trypanosomiasis)	Inhibited	99	45(45.45)	1.27E-24
5	5169	Epstein-Barr virus infection	Inhibited	185	60(32.43)	2.81E-24
6	5140	Leishmaniasis	Inhibited	66	30(45.45)	1.35E-17
**7**	**4933**	**AGE-RAGE signaling pathway in diabetic complications**	**Activated**	**98**	**36(36.73)**	**2.51E-16**
8	5145	Toxoplasmosis	Inhibited	105	36(34.29)	9.25E-16
9	4621	NOD-like receptor signaling pathway	Inhibited	148	42(28.38)	1.74E-14
10	5152	Tuberculosis	Inhibited	158	43(27.22)	2.14E-14
11	5162	Measles	Inhibited	131	38(29.23)	2.93E-14
**12**	**4010**	**MAPK signaling pathway**	**Activated**	**281**	**58(20.64)**	**2.93E-13**
13	5133	Pertussis	Inhibited	67	26(38.81)	1.47E-12
14	5135	Yersinia infection	Inhibited	116	33(28.45)	1.65E-12
**15**	**5235**	**PD-L1 expression and PD-1 checkpoint pathway in cancer**	**Inhibited**	**84**	**28(33.33)**	**4.36E-12**
16	5168	Herpes simplex virus 1 infection	Inhibited	340	59(17.35)	1.02E-11
17	5164	Influenza A	Inhibited	143	36(25.17)	3.43E-11
18	5143	African trypanosomiasis	Inhibited	31	14(45.16)	7.48E-08
19	5144	Malaria	Inhibited	48	14(29.17)	7.92E-06
20	5132	Salmonella infection	Inhibited	193	31(16.06)	1.11E-05
**21**	**4064**	**NF-kappa B signaling pathway**	**Inhibited**	**92**	**20(21.74)**	**1.53E-05**
22	5134	Legionellosis	Inhibited	51	13(25.49)	4.83E-05
23	5010	Alzheimer disease	Activated	311	32(10.29)	0.044888
24	5150	Staphylococcus aureus infection	Inhibited	76	5(6.58)	0.925368
**Pathways identified from GSE22873_MAvM (KO *Myd88_ Ager* *vs.* KO *Myd88*)**						
1	5170	Human immunodeficiency virus 1 infection	Inhibited	194	29(14.95)	6.67E-24
**2**	5169	Epstein-Barr virus infection	Inhibited	185	23(12.43)	1.30E-16
3	5168	Herpes simplex virus 1 infection	Activated	340	21(6.18)	1.39E-09
4	5161	Hepatitis B	Inhibited	149	15(10.07)	4.15E-09
**5**	**4620**	**Toll-like receptor signaling pathway**	**Inhibited**	**87**	**9(10.34)**	**5.25E-06**
6	5142	Chagas disease (American trypanosomiasis)	Inhibited	99	9(9.09)	2.53E-05
**7**	**4933**	**AGE-RAGE signaling pathway in diabetic complications**	**Inhibited**	**98**	**7(7.14)**	**9.55E-05**
8	5235	PD-L1 expression and PD-1 checkpoint pathway in cancer	Inhibited	84	7(8.33)	0.000642
9	5164	Influenza A	Activated	143	8(5.59)	0.001837
10	5135	Yersinia infection	Activated	116	7(6.03)	0.004237
11	5162	Measles	Inhibited	131	7(5.34)	0.006215
12	5010	Alzheimer disease	Inhibited	311	6(1.93)	0.200910
13	5133	Pertussis	Activated	67	3(4.48)	0.204084
14	5132	Salmonella infection	Inhibited	193	4(2.07)	0.291869
15	5140	Leishmaniasis	Inhibited	66	2(3.03)	0.511102
16	5152	Tuberculosis	Activated	158	3(1.90)	0.642447
17	4621	NOD-like receptor signaling pathway	Inhibited	148	2(1.35)	0.683317
18	5143	African trypanosomiasis	Inhibited	31	1(3.23)	0.689362
19	4010	MAPK signaling pathway	Inhibited	281	4(1.42)	0.763496
20	5144	Malaria	Inhibited	48	1(2.08)	0.799437
21	5134	Legionellosis	Inhibited	51	1(1.96)	0.806001
**Pathways identified from GSE22873_MAvA (KO *Myd88_ Ager* *vs.* KO *Ager*)**						
1	5235	PD-L1 expression and PD-1 checkpoint pathway in cancer	Inhibited	84	16(19.05)	2.12E-12
**2**	5170	Human immunodeficiency virus 1 infection	Activated	194	21(10.82)	3.29E-12
3	4010	MAPK signaling pathway	Inhibited	281	22(7.82)	2.25E-10
4	5161	Hepatitis B	Inhibited	149	16(10.74)	7.47E-09
**5**	5135	Yersinia infection	Inhibited	116	14(12.07)	2.57E-08
6	5142	Chagas disease (American trypanosomiasis)	Inhibited	99	12(12.12)	5.28E-07
**7**	5133	Pertussis	Inhibited	67	10(14.93)	1.30E-06
**8**	**4620**	**Toll-like receptor signaling pathway**	**Inhibited**	**87**	**10(11.49)**	**2.73E-06**
**9**	**4933**	**AGE-RAGE signaling pathway in diabetic complications**	**Inhibited**	**98**	**11(11.22)**	**3.83E-06**
10	5140	Leishmaniasis	Inhibited	66	8(12.12)	1.46E-05
11	4621	NOD-like receptor signaling pathway	Inhibited	148	10(6.76)	0.000322
12	4064	NF-kappa B signaling pathway	Inhibited	92	8(8.69)	0.000322
13	5134	Legionellosis	Inhibited	51	6(11.76)	0.000543
14	5145	Toxoplasmosis	Inhibited	105	8(7.62)	0.000738
15	5162	Measles	Inhibited	131	8(6.11)	0.001070
16	5132	Salmonella infection	Inhibited	193	10(5.18)	0.002743
17	5169	Epstein-Barr virus infection	Inhibited	185	9(4.86)	0.002881
18	5152	Tuberculosis	Inhibited	158	7(4.43)	0.032887
19	5144	Malaria	Inhibited	48	4(8.33)	0.034876
20	5164	Influenza A	Inhibited	143	5(3.49)	0.041907
21	5143	African trypanosomiasis	Inhibited	31	3(9.67)	0.063707

22	5168	Herpes simplex virus 1 infection	Inhibited	340	8(2.35)	0.068476
23	5010	Alzheimer disease	Activated	311	9(2.89)	0.089358

True positive knockout (TPKO) signaling pathway (pGFdr < 0.001 or 0.005) and false negative knockout (FNKO) signaling pathway (pGFdr > 0.005) are distinguishable. Bold pathways are discussed in the main text.

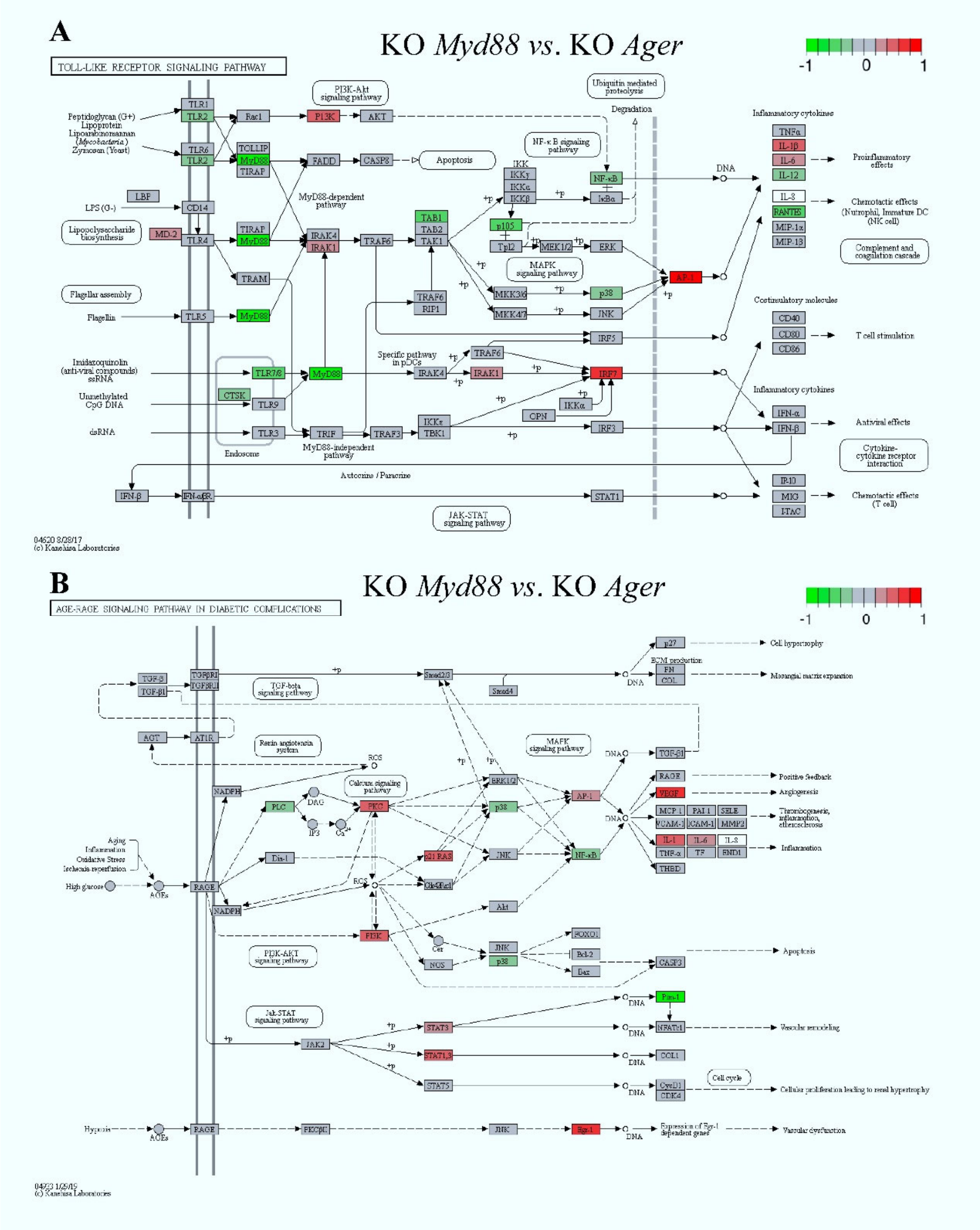

FIGURE S5 Comparison of the rendered target TPKO signaling pathways identified by the SPIA method from GSE22873_MvA (KO *Myd88 vs.* KO *Ager*). Explanations are presented in the main text. (A) The Toll-like receptor signaling pathway (mmu04620). (B) The AGE-RAGE signaling pathway in diabetic complications (mmu04933).
